# Coronaviruses SARS-CoV, MERS-CoV, and SARS-CoV-2 helicase inhibitors: a systematic review of *invitro* studies

**DOI:** 10.1016/j.jve.2023.100327

**Published:** 2023-05-26

**Authors:** Nimer Mehyar

**Affiliations:** a King Saud Bin Abdulaziz University for Health Sciences, Riyadh, Saudi Arabia; b King Abdullah International Medical Research Center, Riyadh, Saudi Arabia; c King Abdulaziz Medical City, Ministry of National Guard-Health Affairs, Riyadh, Saudi Arabia

**Keywords:** Coronavirus, Helicase, Inhibitors, nsp13, In vitro assay, Systematic review

## Abstract

**Introduction:**

The recent outbreak of SARS-CoV-2 has significantly increased the need to find inhibitors that target the essential enzymes for viral replication in host cells. This systematic review was conducted to identify potential inhibitors of SARS-CoV, MERS-CoV, and SARS-CoV-2 helicases that have been tested by *in vitro* methods. Their inhibitory mechanisms are discussed in this review, in addition to their cytotoxic and protective properties.

**Methods:**

The databases PUBMED/MEDLINE, EMBASE, SCOPUS, and Web of Science were searched using different combinations of the keywords “helicase”, “nsp13”, “inhibitors”, “coronaviridae”, “coronaviruses”, “virus replication”, “replication”, and “antagonists and inhibitors".

**Results:**

A total of 6854 articles were identified. Thirty-one were included into this review. These studies reported on the inhibitory effects of 309 compounds on SARS-CoV, MERS-CoV, and SARS-CoV-2 helicase activities measured by *invitro* methods. Helicase inhibitors were categorized according to the type of coronavirus and tested enzymatic activity, nature, approval, inhibition level, cytotoxicity, and viral infection protective effects. These inhibitors are classified according to the site of their interaction with coronavirus helicases into four types: zinc-binding site inhibitors, nucleic acid-binding site inhibitors, nucleotide-binding site inhibitors, and inhibitors with no clear interaction site.

**Conclusion:**

Evidence from *in vitro* studies suggests that helicase inhibitors have a high potential as antiviral agents. Several show good antiviral activity while maintaining moderate cytotoxicity. These inhibitors should be clinically investigated to determine their efficacy in treating coronavirus infections, particularly SARS-CoV-2.

## 1 Introduction

**Fig. 1:**
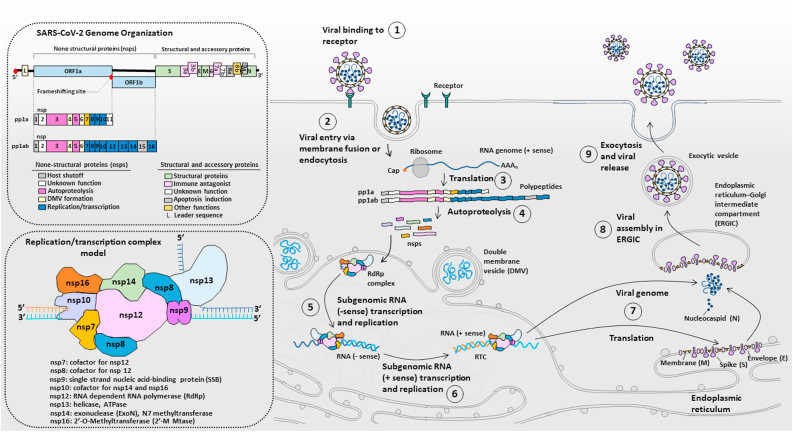
Involvement of nsp13 (helicase) in SARS-CoV-2 replicative cycle. Encoded ORFs and synthesized polypeptides contain nsp13 (upper subset). Structural organization of the replication/transcription complex (lower subset).

Since the SARS-CoV outbreak in 2003 and the MERS-CoV one in 2014, much attention has been devoted to RNA helicases as potential antiviral targets against pathogenic coronaviruses^1^^,^^2^, particularly after the SARS-CoV-2 pandemic in 2019.^3^ The growing interest in coronavirus helicases as a potential target was manifested by the increasing number of studies examining the inhibitory effects of different compounds by in silico, *in vitro*, and *in vivo*-based investigations.^4^ SARS-CoV, MERS-CoV, SARS-CoV, and other pathogenic coronaviruses are members of the Coronaviridae family in the order Nidovirales.^5^ Coronarovirus genomes are made of several open reading frames that encode structural proteins, non-structural proteins (nsps), and several accessory proteins.^6^ The entry of pathogenic coronavirus into host cells is enabled by the interaction of the viral spike protein (S1) with a variety of cellular receptors in the plasma membrane of host cells, like the angiotensin-converting enzyme 2 (ACE2) in SARS-CoV infection and SARS-CoV-2 and the dipeptidyl-peptidase 4 (DPP4) in MERS-CoV infection.^7^ In the post-entry phase, viral genomic RNA, which contains two large open reading frames, ORF1a and ORF1b, is released into the host cell which are then translated into pp1a and pp1ab polypeptides. Cleavage of these peptides by the main viral proteases produces several nsps that are essential for the replication/transcription complexes (RTCs). These are responsible for the viral gene replication, synthesis of new viral proteins, and viral assembly.^8^ Nsp13 plays a central role in RTC formation. It engages the RTC after its release from the polypeptide pp1ab by proteolytic cleavage. In this assembly process, nsp13 interacts with the RNA dependent RNA polymerase (RdRP) nsp12 which stimulates nsp13 activity (Fig. 1).^9^ Nsp13 belongs to the helicase superfamily 1 (SF1) and shares many structural similarities with the eukaryotic Upf1 helicase, which is essential for nonsense-mediated mRNA decay in cells.^10^ Like other members of the SF1 superfamily, nsp13 is a bifunctional enzyme that uses the released energy from nucleoside triphosphate (NTP) hydrolysis to derive dsRNA unwinding. NTP hydrolysis is also required for the RNA capping mechanism.^11^ In addition to dsDNA and/or dsRNA substrate unwinding during viral replication/transcription and RNA capping processes, nsp13 could also be essential for DNA or RNA secondary structure remodeling, nucleic acid-bound protein displacement, and translocation along the double-stranded nucleic acid.^12^

Pathogenic coronaviruses SARS-CoV-2, MERS-CoV, and SARS-CoV have RNA helicases. They share a high degree of sequence similarity (84%–99%) and a close structural organization.^13^ Several domains, critical for helicase activity, are highly conserved in the three pathogenic coronaviruses such as 1A, 2A, 1B, the stalk domain, and zinc-binding domain (ZBD).^14^^,^^15^ Crystallographic fragment screening of the SARS-CoV-2 helicase has revealed several pockets that could be used for structure-based drug design, particularly the nucleotide and nucleic acid-binding sites^16^ (Fig. S1). The energy released by ATP hydrolysis is used by viral RNA helicases to unwind the double-stranded nucleic acid into two single-stranded nucleic acids (ss) in a 5′–3′ direction.^17^ The binding of ATP-Mg2+ and subsequent hydrolysis of the phosphate and release of ADP from the nucleotide-binding site induce nsp13 conformational shift from an activated “closed” state that binds tightly to RNA 3′-end through the 2A domain to an “open” state. The “open” state conformation has a low binding affinity for the 2A domain and high binding affinity for the 1A domain to the RNA. This ATP-hydrolysis-induced shift of binding affinities between the 2A and 1A domains causes an “inchworm"-like sliding of the nsp13 alongside the single-stranded RNA.^16^

Due to the fast spread of the COVID-19 pandemic, a lot of pressure was placed on drug discovery to produce effective and safe antiviral agents.^18^ These efforts were mainly focused on antiviral agents targeting viral entry and the replicative phases in the host cells. As a result, the majority of newly-reported compounds targeted the spike protein interactions with the ACE2 receptor as well as the main proteases and RNA polymerase .^19^^,^^20^ Helicases, on the other hand, have received less attention despite their critical role in the viral life cycle.^3^^,^^4^^,^^21^ Furthermore, rather than the time-consuming process of developing new drugs, a significant part of the research was also devoted to drug repurposing.^22^ Because the coronavirus protein and enzyme assay methods are not fully standardized, expensive, and time-consuming, drug discovery efforts have relied heavily on virtual screening and, to a lesser extent, high throughput screening.^23^ The use of structure-based virtual screening to identify new potential inhibitors was significantly enabled by the rapid release of high-resolution crystal structures of the viral proteins and exponential advancements in bioinformatics and computational modeling.^24^ Virtual screening of the SARS-CoV-2 helicase predicted the binding of several clinically approved drugs to the helicase active site.^25^ Several other compounds and FDA-approved drugs were reported to inhibit the coronavirus helicase activity. Most of these compounds were detected by fluorescence resonance energy transfer (FRET)-based assays.^26^^,^^27^ At the time of this analysis, the RdRp inhibitor, remdesivir, was the only antiviral drug approved by the FDA to treat SARS-CoV-2. None of the reported helicase inhibitors were clinically approved.^28^

Experimentally tested compounds against coronavirus helicases are diverse in their nature and mechanisms of action. In addition, many of these were tested with the helicases of one pathogenic coronavirus, but not all of them. With the large number of potential helicases suggested by virtual screening methods, it became essential to recognize whether an inhibitor had been tested against the enzymatic activity or not. Providing organized information about *in vitro* tested compounds and their properties could benefit the search for new coronavirus antivirals in many ways. In addition to profiling inhibitors according to the virus type, it would help understanding their mechanisms of action. This systematic review aims to summarize the available data on compounds experimentally tested against the activity of the helicases of the main three pathogenic coronaviruses.

## 2 Methods

All protocols and procedures of this review, including the search, study selection, data extraction, and result analysis, were performed in accordance with the recommendations of the Preferred Reporting Items for Systematic Reviews and Meta-Analyses (PRISMA) statement.^29^

### 2.1 Search strategies

*In vitro* studies that identified compounds targeting coronavirus helicase activity were screened using PUBMED/MEDLINE, EMBASE, SCOPUS, and WEB OF SCIENCE databases. During PUBMED/MEDLINE and EMBASE searches, the Medical Subject Headings (MeSH) terms were used to define the descriptors. The keywords “helicase”, “nsp13”, and “inhibitors” were used in combination with the descriptors “coronaviridae”, “coronaviruses”, “virus replication”, “replication”, and “antagonists and inhibitors” using the connector “AND” between them, for example: ((coronaviridae (MeSH Terms)) AND (virus replication (MeSH Terms))) AND (helicase). In the SCOPUS and WEB OF SCIENCE searches, no descriptors were used. Combinations of the keywords: helicase, nsp13, inhibitors, coronavirus, and replication were used with the connector “AND” between terms, for example: ((coronavirus AND replication AND nsp13)). The search was limited to English-language articles published up to January 31, 2022. The forward snowballing method was used to extend the study up to December 1, 2022.

### 2.2 Inclusion and exclusion criteria

Studies that used *in vitro* methods to measure the inhibitory effects of chemicals on the coronavirus helicase were selected for this review. Initially, study titles were screened, and all duplicates, non-English studies, and non-original research were excluded. Abstracts and paper full texts were then analyzed, and studies excluded according to the following criteria: (1) those about chemicals showing inhibitory effects against non-viral helicases; (2) those about chemicals showing inhibitory effects against viral helicases other than coronaviruses; (3) those about chemicals showing inhibitory effects against coronavirus proteins and enzymes other than helicase; and (4) those using computational methods to identify potential inhibitors.

### 2.3 Data selection

Study keywords were searched by two independent researchers on databases with the help of the librarian of the Faculty of Basic Sciences and Health Professions at King Saud bin Abdulaziz University for Health Sciences. Search results were initially screened. Titles, and keywords were analyzed, and a list of pre-selected studies was collected. Abstracts and texts of pre-selected studies were read to confirm compliance with inclusion criteria by two independent researchers, with a final list of selected studies. The degree of agreement between the two researchers was estimated and considered adequate at substantial agreement (k = 0.61–0.8).^30^ In case of disagreement, the two researchers after discussion achieved a conflict resolution by mutual agreement.

### 2.4 Data analysis

Selected studies were fully analyzed. Data of interest, including authorship, structure, names, and CAS number of *in vitro* tested compounds, virus type, nsp13 kinetic parameters, unwinding activity inhibition parameters, ATPase activity inhibition parameters, parameters of effective protection concentration of infected cells (EC50), parameters of uninfected cell cytotoxicity, various assay conditions, interactions of inhibitors with nsp13 binding sites, and evidence of proposed interactions, was reported.

## 3 Results

### 3.1 Study selection

**Fig. 2:**
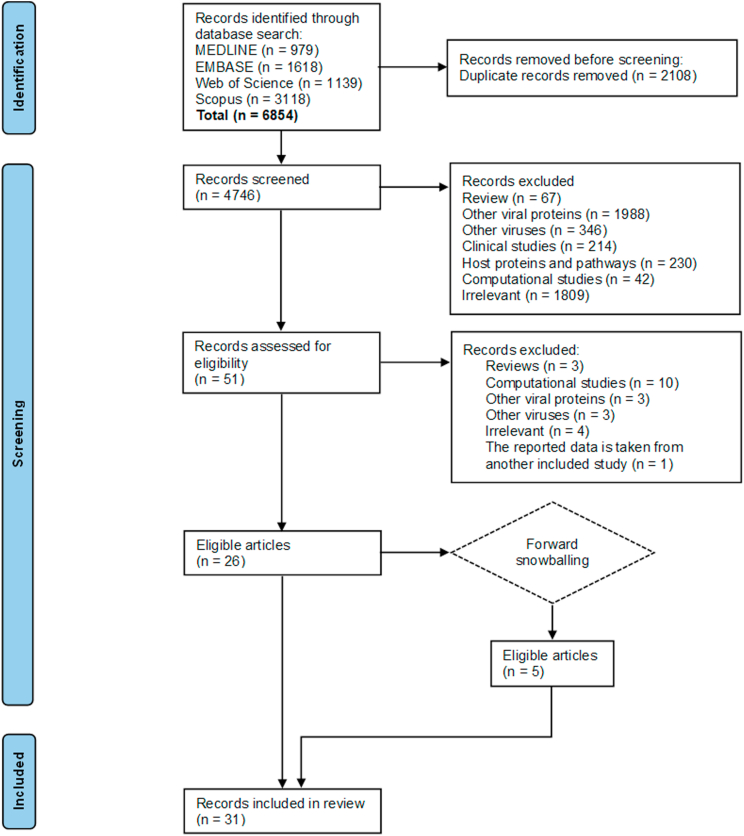
Study selection: PRISMA flow diagram.

A total of 6854 studies (979 from PubMed/MEDLINE, 1618 from EMBASE, 1139 from the Web of Science, and 3118 from Scopus) published between 2004 and 2022 were identified in the initial search of online databases. After removing duplicates, the remaining 4746 articles were used for screening. Titles of these articles were screened in accordance with inclusion criteria. At the end of this screening stage, a total of 4697 articles were excluded. Abstracts of the remaining 50 studies were extensively reviewed by the two researchers. The degree of agreement on the selected papers was substantial (kappa = 0.79). Only one study published before 2004 appeared in the list, and was later excluded due to irrelevance.^25^ There was a conflict over the inclusion of five articles.^26^^,^^31^, ^32^, ^33^, ^34^ Abstracts and full texts of these articles were re-examined by both researchers who agreed that two articles^26^^,^^33^ met the inclusion criteria. The remaining three articles^31^, ^32^, ^33^, ^34^ were eliminated Table S1. At the end of this screening stage, three review articles^32^^,^^35^^,^^36^, nine computational studies^31^^,^^34^^,^^37^, ^38^, ^39^, ^40^, ^41^, ^42^, ^43^, three none-helicase studies^44^, ^45^, ^46^, three non-coronavirus studies^47^, ^48^, ^49^ and four unrelated studies^25^^,^^50^, ^51^, ^52^ were excluded from the analysis. One study was excluded after full review since it was a computation-based.^53^ Another study^54^ was excluded as it did not present any helicase inhibition data but rather shared data from a previous publication, which was already included into the study. The twenty-six eligible studies were used as a starting set for forward snowballing search.^26^^,^^27^^,^^33^^,^^55^, ^56^, ^57^, ^58^, ^59^, ^60^, ^61^, ^62^, ^63^, ^64^, ^65^, ^66^, ^67^, ^68^, ^69^, ^70^, ^71^, ^72^, ^73^, ^74^, ^75^, ^76^, ^77^ As a result, five studies were found to fit the inclusion criteria of this review.^78^, ^79^, ^80^, ^81^, ^82^ These were reviewed by the two researchers who mutually agreed to include them into the review. The final thirty-one studies describing the *in* *vitro* assays of 309 chemical compounds on the unwinding and ATPase activities of SARS-CoV, MERS-CoV, and SARS-CoV-2 helicases were used for the full-text analysis and extraction of data of interest (Fig. 2). Tested but not reported compounds from commercial and in-house screening libraries were not included into this analysis.

### 3.2 Study characteristics

Studies included into this systematic review investigated the inhibitory effects of natural and synthetic compounds on the activity of coronavirus helicases using *in vitro* methods. These were distributed among the three types of pathogenic coronaviruses: sixteen studies tested the inhibition of the SARS-CoV helicase, two the MERS-CoV helicase, and thirteen the SARS-CoV-2 helicase. One study tested SARS-CoV and MERS-CoV helicases, and another one MERS-CoV and SARS-CoV-2 helicases (Table 1, Table 2, Table 3). Twenty-three studies tested the effect of compounds on the unwinding and ATPase activity, six studies the unwinding activity alone, and two studies the ATPase activity only. All studies that measured ATPase activity used a colorimetric assay to measure the released phosphate, except for two that used a luciferase-based assay. The unwinding activity was measured using a variety of substrates and methods. Twenty-one studies measured the fluorescence resonance energy transfer (FRET) produced by labeled dsDNA separation in solution, four the FRET produced by dsDNA and dsRNA separation in solution, three in gel conditions one the FRET of dsDNA and dsRNA separation in gel conditions and one study the separation of radiolabeled dsDNA. Ten studies directly measured the kinetic parameters of the used helicases, while two referred to previous work done to report the kinetic parameters of the same helicases.^83^, ^84^, ^85^ Kinetic parameters of the helicases used in the remaining nineteen studies were not reported. Fourteen studies tested selected inhibitors for the protection of virus-infected cells: eleven used VERO-6 cells for viral infection protection assay, two FRhK-1 cells, one the A549-ACE2 cells, and one study the replicon model cell. Out of thirteen studies, five used qPCR to quantify viral RNA in infected VERO cells after exposure to potential helicase inhibitors. The remaining sixteen studies did not test the viral infection protection properties of their reported inhibitors. As for cytotoxicity, seventeen studies tested selected inhibitor cytotoxic effects. Seven studies looked at the cytotoxicity of VERO-6 cells. One study measured the cytotoxic effects on Caco2 cells and another one on replicon model cells in addition to VERO-6 cells. Several studies used other types of cells to measure the cytotoxic effects of their selected inhibitors: two used FRhK-1, three HS27 cells, three W-38 cells, one MCF10A cells, and one A549-ACE2 cells (Fig. S2).

**Table 1:** *In vitro* inhibition studies of the SARS-CoV helicase.

Reference	Compound	Unwinding Inhibition IC_50_ (μM)^a^	ATPase Inhibition IC_50_ (μM)^b^	Infection Inhibition EC_50_ (μM)^c^	Cytotoxicity CC_50_ (μM)^d^
^55^	HE602	Inhibition at 20 (SDS-PAGE)	6.9	6.0	>50
	VE607	–	>50	–	–
	MP576	–	>50	–	–
^56^	Iodobananin	7.0	0.54	–	–
	Vanillinbananin	2.7	0.68	–	–
	Bananin	3.0	2.3	<10 (FRhK-4)	390 (FRhK-4)
				<10 (RT-qPCR, after 12hr incubation)	
	Eubananin	5.4	2.8	–	–
	Ansabananin	>100	51	–	–
	Adeninonananin	>100	>100	–	–
^57^	Complex 2	<3.0 (SDS-PAGE)	0.5 ± 0.1	–	–
	Complex 1	<3.0 (SDS-PAGE)	0.6 ± 0.1	–	–
	Complex 6	<5.0	IC_50_ < 5.0	–	–
	Complex 7	<5.0	5.0	–	–
	Complex 5b	<5.0	5.0	–	–
	Complex 5a	5.0 ± 0.8	4.7 ± 0.6	–	–
	Complex 3	7.2 ± 0.8	6.4 ± 1.0	–	–
	Complex 4	11.0 ± 1.8	7.4 ± 1.1	–	–
^58^	Ranitidine bismuth citrate (RBC)	0.25–0.75 (SDS-PAGE) 0.6	0.377 ± 0.092 (No RNA) 0.248 ± 0.075 (+ poly-U_24_) 0.350 ± 0.082 (+ poly-T_24_)	5.9 ± 4 (FRhK-4) 50 ± 8 (FRhK-4) 12.5–25 (RT-qPCR, after 24hr incubation)	5000 (FRhK-4)
	Bismuth tricysteine complex	–	75% inhibition at 1	–	–
	Bismuth nitrate	–	75% inhibition at 1	–	–
	Bismuth nitrilotriacetate	–	75% inhibition at 1	–	–
	Bismuth citrate (BC)	–	50% inhibition at 1	–	–
	Bismuth acetohydroxamate	–	80% inhibition at 50	–	–
	Bismuth ethylenediaminetetraacetate	–	No inhibition at 50	–	–
^59^	ES15 aptamers pool	0.0012	≈0.0770 (No poly rU)	–	–
			No activation (+ poly rU)		
^60^	Capped NG8 (3′-inverted thymidine aptamer NG8)	0.0175	**ATPase Activation,**$K_{m}^{App}$ (μM)0.0268 nM	–	–
	Capped NG8 (3′-biotin aptamer NG8)	0.0558	0.0582	–	–
	NG1	0.0877	0.0208	–	–
	NG8	0.0910	0.0054	–	–
	NG3	0.1208	0.0133	–	–
	G8	>1	0.0268	–	–
	G5	>1	0.0582	–	–
	NG2, NG4, NG5, NG6, NG7, NG9, NG10, NG11, NG12, NG13, NG14, G1, G2, G3, G4, G6, G7, G9	No inhibition	No activation	–	–
	Unmodified random sequence	>1	0.1220	–	–
	3′-inverted thymidine random sequence	>1	>1	–	–
	3′-biotin random sequence	>1	>1	–	–
^61^	Derivative 7	5.4 ± 0.1	>50	–	–
	Derivative 8	11.0 ± 0.6	>50	–	–
	Derivative 6	28.7 ± 2.3	>50	–	–
	Derivative 1	39.9 ± 0.5	41.3 ± 2.7	–	–
	Derivative 5	>50	>50	–	–
	Derivative 3	>50	>50	–	–
	Derivative 2	>50	>50	–	–
	Derivative 4	13.6 ± 0.3	24.4 ± 1.0	–	–
^62^	Derivative 4c	2.7 ± 0.1	25.4 ± 1.5	–	–
	Derivative 4a	4.1 ± 0.3	20.9 ± 0.5	–	–
	Derivative 4b	5.2 ± 0.4	>50	–	–
	Derivative 4f	8.1 ± 0.3	42.9 ± 5.4	–	–
	Derivative 3a	8.1 ± 0.3	>50	–	–
	Derivative 4d	9.3 ± 0.4	>50	–	–
	Derivative 4e	15.4 ± 0.8	>50	–	–
	Derivative 3b	>50	>50	–	–
	Derivative 2c	>50	>50	–	–
	Derivative 2b	>50	>50	–	–
	Derivative 2a	>50	>50	–	–
^63^	Derivative 5e	11	4	–	>50 (HS27)
	Derivative 1	11	>50	–	>50 (HS27)
	Derivative 5f	31	23	–	>50 (HS27)
	Derivative 5b	40	10	–	>50 (HS27)
	Derivative 5c	>50	28	–	>50 (HS27)
	Derivative 5g	>50	37	–	>50 (HS27)
	Derivative 4	>50	>50	–	>50 (HS27)
	Derivative 3	>50	>50	–	>50 (HS27)
	Derivative 5d	>50	>50	–	>50 (HS27)
	Derivative 5a	>50	>50	–	>50 (HS27)
^64^	SSYA10-001	5.3 ± 0.40 (SDS-PAGE) 5.7 ± 0.74 (dsRNA, SDS-PAGE) 5.6 ± 0.50 (viral RNA, SDS-PAGE)	No inhibition at 40	8.95 ± 0.86 (Replicon cell, RT-qPCR)	>250 (Replicon cell)
	SSYA10-002	50% inhibition at 20	–	–	–
^65^	SSYA10-001	5.9 (Wild type)	–	∼7 (SARS-CoV)	No effect at 500
		15 (Mutant Y277A)	–	–	–
		50 (Mutant K508A)	–	–	–
^66^	Scutellarein	No inhibition at 10	0.86 ± 0.48	–	No effect at 2 (MCF10A)
	Myricetin	No inhibition at 10	2.71 ± 0.19	–	No effect at 2 (MCF10A)
	Myricitrin	No inhibition at 10	20% inhibition at 10	–	–
	Amentoflavone	No inhibition at 10	20% inhibition at 10	–	–
	Diosmetin-7-O-Glc-Xy	No inhibition at 10	20% inhibition at 10	–	–
	Taraxerol	No inhibition at 10	20% inhibition at 10	–	–
	58 natural products (See supplementary)	No inhibition at 10	No inhibition at 10	–	–
^67^	Baicalein	No inhibition at 10	60% inhibition at 10 0.47 ± 0.09	–	–
	Luteolin	No inhibition at 10	20% inhibition at 10	–	–
	Oroxylin A	No inhibition at 10	20% inhibition at 10	–	–
	Apigenin	No inhibition at 10	No inhibition at 10	–	–
	Baicalin	No inhibition at 10	No inhibition at 10	–	–
	Wogonin	No inhibition at 10	No inhibition at 10	–	–
^68^	EMMDPD	41.6 ± 2.3	8.66 ± 0.26	–	No effect at 80 (WI-38)
^69^	DTPMPA	32.9 ± 1.0	1.19 ± 0.16	–	No effect at 80 (WI-38)
^70^	FSPA	13.2 ± 0.9	2.09 ± 0.3	–	No effect at 40 (WI-38)

a Unwinding inhibition assay: helicase activity is measured by the separation of fluorophore- and quencher-labeled dsDNA in the presence of ATP and MgCl2. The formation of free ssDNA can be monitored by SDS-PAGE separation methods or by measuring the increase in fluorescence signal in solution. The inhibitory effects of tested compounds are usually measured by incubating the enzyme with different concentrations of these compounds prior to the activity assay (for specific details of each article, see Table S4).

b ATPase inhibition assay: ATPase activity is measured by the release of free orthophosphate from ATP in the presence of dsDNA and MgCl2. At the end of the reaction, malachite green dye and ammonium molybdate are added to the reaction mixture. The released phosphate is monitored by the formation of the green complex between Malachite Green, molybdate, and free orthophosphate. The inhibitory effects of tested compounds are usually measured by incubating the enzyme with different concentrations of these compounds prior to the activity assay (for specific details of each article, see Table S4).

c Infection inhibition assay: infection inhibition is measured by monitoring the coronavirus-infected cells cytopathies development over time. The inhibitory effects of tested compounds are usually measured by incubating the virus with different concentrations of these compounds prior to cell infection.

d Cytotoxicity assay: the cytotoxic effects of tested compounds are measured by monitoring the normal cells (uninfected) cytopathies development with time in the presence of different concentrations of these compounds.

**Table 2:** *In vitro* inhibition studies of the MERS-CoV helicase.

Reference	Compound	Helicase Activity IC_50_ (μM)^a^	ATPase Activity IC50 (μM)^b^	Infection Inhibition EC_50_ (μM)^c^	Cytotoxicity CC_50_ (μM)^d^
		**Second substrate: dsRNA**			
^27^	Epirubicin HCl	0.73 ± 0.06	–	–	–
	Doxorubicin HCl	0.73 ± 0.02	–	–	–
	Daunorubicin HCl	1.32 ± 0.09	–	–	–
	Mitoxantrone 2HCl	1.6 ± 0.05	–	–	–
	Idarubicin HCl	1.65 ± 0.20	–	–	–
	Otilonium bromide	19.6 ± 2.3	–	–	–
	Caspofungin acetate	21.2 ± 5.64	–	–	–
	Tolcapone	23.3 ± 1.9	–	–	–
	Sunitinib malate	26.0 ± 1.38	–	–	–
	Ethacridine lactate monohydrate	30.8 ± 2.81	–	–	–
	Masitinib	58.1 ± 10.8	–	–	–
	Bazedoxifene HCl	43.3 ± 0.17	–	–	–
	Ruxolitinib	168 ± 26.5	–	–	–
	Raloxifene HCl	193 ± 19.4	–	–	–
	Diminazene aceturate	502 ± 15.8	–	–	–
^65^	SSYA10-001	–	–	∼25 (MERS-CoV) ∼12 (MHV)	–
^71^	Derivative 16	2.5	0.47	–	–
	Derivative 12	3.0	0.51	–	–
	Derivative 15	4.3	2.73	–	–
	Derivative 11	5.7	3.9	–	–
	Derivative 14	6.3	4.0	–	–
	Derivative 13	9.0	5.3	–	–
	4-Amino-5-hydrazino-4H-1,2,4-triazole-3-thio	12.4	8.9		
	Derivative 10	>100	58	–	–
	Derivative 8	>100	87.64	–	–
	Derivative 1	>100	>100	–	–
	Derivative 2	>100	>100	–	–
	Derivative 3	>100	>100	–	–
	Derivative 4	>100	>100	–	–
	Derivative 5	>100	>100	–	–
	Derivative 6	>100	>100	–	–
	Derivative 7	>100	>100	–	–
	Derivative 9	>100	>100	–	–
^74^	Clofazimine	<50	–	1.48 ± 0.17	–

a See the footnote of Table 1.

b See the footnote of Table 1.

c See the footnote of Table 1.

d See the footnote of Table 1.

**Table 3:** *In vitro* inhibition studies of the SARS-CoV-2 helicase.

Reference	Compound	Unwinding Inhibition, IC_50_ (μM)^a^		ATPase Inhibition IC_50_ (μM)^b^		Infection Inhibition EC_50_ (μM)^c^	Cytotoxicity CC_50_ (μM)^d^
^26^	Epirubicin HCl	0.31 ± 0.03		–		–	–
	Doxorubicin HCl	0.40 ± 0.02		–		–	–
	Daunorubicin HCl	0.46 ± 0.05		–		–	–
	Mitoxantrone 2HCl	0.70 ± 0.07		–		–	–
	Idarubicin HCl	0.72 ± 0.09		–		–	–
	Zafirlukast	16.3 ± 2.27		–		Inhibition at 25 μM 17 (cycle threshold value of RT-qPCR) at 25	No effect at 25 μM
	Masitinib	16.7 ± 1.08		–		–	–
	Ethacridine lactate monohydrate	22.9 ± 7.25		–		–	–
	Sunitinib malate	25.6 ± 1.08		–		–	–
	Diminazene aceturate	95.3 ± 3.00		–		–	–
	Otilonium bromide	214 ± 72		–		–	–
	Montelukast	No inhibition at 25		–		Inhibition at 25 μM 13 (cycle threshold value of RT-qPCR) at 25	No effect at 25 μM
	Ruxolitinib	No inhibition at 100		–		–	–
	Raloxifene HCl	No inhibition at 100		–		–	–
	Caspofungin acetate	No inhibition at 100		–		–	–
	Tolcapone	No inhibition at 100		–		–	–
	Bazedoxifene HCl	No inhibition at 100		–			
	Darunavir	No inhibition at 100		–		–	–
	Mezlocillin sodium	No inhibition at 100		–		–	–
	Bosentan hydrate	No inhibition at 100		–		–	–
	Lapatinib	No inhibition at 100		–		–	–
	Sivelestat sodium tetrahydrate	No inhibition at 100		–		–	–
	Omipaslisib (GSK458)	No inhibition at 100		–		–	–
	Tianeptine	No inhibition at 100		–		–	–
	Pazopanib	No inhibition at 100		–		–	–
^33^	Lumacaftor	–		0.3		–	–
	Cepharanthine	–		0.4		–	–
	Emend	–		No inhibition at 1000		–	–
	Nilotinib	–		No inhibition at 1000		–	–
	Irinotecan	–		No inhibition at 1000		–	–
	Enjuvia	–		No inhibition at 1000		–	–
	Cefoperazone	–		No inhibition at 1000		–	–
	Dihydroergotamine	–		No inhibition at 1000		–	–
	Zelboraf	–		No inhibition at 1000		–	–
^72^	RBC	**Second substrate: dsRNA**					
		∼0.90		∼1.2		–	–
	Colloidal bismuth subcitrate (CBS)	∼2.5		∼2.5		–	–
	BC	Much less than RBC and CBS		∼25% inhibition at 10		–	–
^73^	RBC	0.74 ± 0.13		0.69 ± 0.12		2.3 ± 0.5	2243 ± 43 (VERO) 2486 ± 65 (Caco2)
	CBS	1.24 ± 0.02		1.88 ± 0.12		4.6 ± 0.4	3254 ± 21 (VERO) 3740 ± 125 (Caco2)
	Bi (TPyP)	2.64 ± 0.16		4.68 ± 1.39		7.5 ± 0.9	>400 (VERO) > 400 (Caco2)
	Bi (TPP)	3.69 ± 0.26		2.39 ± 0.02		3.9 ± 1.2	>400 (VERO) > 400 (Caco2)
^74^	Clofazimine	<10 (dsDNA) < 10 (dsRNA)		–		0.31	–
^75^	Ebselen	–		0.299		50% inhibition at 25	–
	Disulfiram	–		0.411		30% inhibition at 50	–
^76^	Xanthorrhizol	No inhibition at 10		–		1.28	No effect at 20 μM
^77^		**Assay condition (C)**^e^		–		–	
		**No Tween-20**	**0.02% Tween-20**				
	NF 023	0.67	0.64				
	Surmain	0.74	1.6	–		9.9	Inhibition at 1 × 10^5^–3 × 10^5^ μM
	Navitoclax	0.74	>250	–		–	
	Adapalene	1.3	5.5	–		–	
	Avasimibe	1.3	17	–		–	
	Gossypol	1.3	31	–		–	–
	Shikonin	1.4	78	–		14	Inhibition at 1 × 10^5^–3 × 10^5^ μM
	Zafirlukast	1.5	50	–		–	–
	TCID	1.9	15	–		–	–
	Cintirorgon	2.1	41	–		–	–
	Idasanutlin	2.3	11	–		–	–
	GW-7647	2.3	25	–		–	–
	Linoleic acid	4.3	36	–		–	–
	Oleic acid	14	117	–		–	–
	Fenretinide	7.3	>250	**-**		–	–
	FPA-124	8.5	8.4	–		–	–
		**Assay conditions**					
		**(A)**^e^**No Tween-20**	**(B)**^e^**0.02% Tween-20**				
	PDK1/Akt/Flt dual pathway inhibitor	1.1	0.90				
	Evans blue	1.2	1.9				
	ABT-737	1.2	>250	–		–	–
	PPNDS	2.0	1.6	–		–	–
	TW-37	2.8	109	–		–	–
	RO8994	2.9	>250	–		–	–
	A-385358	3.3	>250	–		80.5	No effect at 300 μM
	CARM1-IN-1	4.2	126	–		–	–
	Adomeglivant	5.3	103	–		–	–
	Diphenyl Blue	5.6	4.7	–		–	–
	Elaidic acid	8.6	>250	–		–	–
	Eicosapentaenoic acid	14	69	–		–	–
	Venetoclax/ABT-199	25	>250	–		–	–
	Linifanib/ABT-869	26	230	–		–	–
	Ceftiofur HCl	>250	>250	–		–	–
	SB-366791	>250	>250	–		–	–
	Leucovorin Calcium	>250	>250	–		–	–
		**Assay condition (D)**^e^					
		**No Tween-20**	**0.02% Tween-20**				
	SSYA10-001	7.5	28				
	Hypocrellin A	7.5	28				
	TAK875	12	>250				
	Elvitegravir	19	72	–		–	–
	Myricetin	22	>250	–		–	–
	NVP-BVU-972	24	125	–		–	–
	Eplerenone	75	>250	–		–	–
	Fluocinolone Acetonide	>250	No inhibition	–		31.5	No effect at 300 μM
	Omapatrilat	>250	No inhibition	–		–	–
	ADL5859	>250	No inhibition	–		–	–
	Nedocromil	No inhibition	No inhibition	–		–	–
	Lifitegrast	No inhibition	No inhibition	–		–	–
	SKF 89145	No inhibition	No inhibition	–		–	–
	Mitoxantrone	Quenching	Quenching	–		–	–
	6-Hydroxy-DL-DOPA	Quenching	Quenching	–		–	–
	Doxorubicin	Artifact	Artifact	–		–	–
^78^		**(-) BSA/TCEP**	**(+) BSA/TCEP**	**(-) BSA/TCEP**	**(+) BSA/TCEP**		
	Myricetin	0.41 ± 0.11	19.9 ± 2.3	>30	>30	>100	>100
	Quercetin	0.53 ± 0.13	10.2 ± 1.4	>30	>30	>100	>100
	Flavanone	0.52 ± 0.24	6.48 ± 0.53	>30	>30	>100	>100
	Kaempferol	0.76 ± 0.16	19.0 ± 2.1	>30	>30	>100	>100
	Licoflavone C	1.34 ± 0.31	9.9 ± 0.5	24.6 ± 3.8	24.6 ± 3.8	>100	>100
	Flavanone-7-O-glucoside	2.88 ± 0.88	70.6 ± 3.2	>30	>30	>100	>100
	Baicalein	2.90 ± 1.0	10.2 ± 1.1	>30	>30	>100	>100
	Diosmetin	10.6 ± 5.5	57.8 ± 1.2	>30	>30	>100	>100
	Prunetin	11.5 ± 1.7	>100	>30	>30	>100	>100
	Wogonin	24.9 ± 5.5	74.9 ± 8.3	>30	>30	–	–
	Dihydromyricetin	25.6 ± 7.7	>100	>30	>30	–	–
	Catechin	>30	–	>30	–	–	–
	Apigenin-7-O-glucoside	>30	–	>30	–	–	–
	Kaempferol-3-O-rutinoside	>30	–	>30	–	–	–
	Luteoline-4-O-glucoside	>30	–	>30	–	–	–
	Luteoline-7-O-glucoside	>30	–	>30	–	–	–
	Quercetin-3-O-β-glucoside	>30	–	>30	–	–	–
	Rutin	>30	–	>30	–	–	–
	Gallic acid	>30	–	>30	–	–	–
	Resveratrol	>30	–	>30	–	–	–
	Ferulic acid	>30	–	>30	–	–	–
	SSYA10-001	0.05 ± 0.02	1.73 ± 0.34	>3	>3	–	–
	GC376	>100	–	>100	–	0.28 ± 0.04	>100
^79^	SSYA10-001	0.046 ± 0.015		>3		–	–
	6g	0.42 ± 0.23		>30		8.8 ± 5.6	>100
	7k	1.41 ± 0.64		>30		2.7 ± 1.9	23.8 ± 5.7
	6f	>30		>30		7.9 ± 5.6	>100
	9j	>30		>30		5.9 ± 2.4	>100
	F243	No inhibition		No inhibition		–	–
^80^	C1	–		**(+) ssDNA** 6 ± 0.5	**(-) ssDNA** No inhibition	–	–
	C5	–		27 ± 1	33 ± 2	–	–
	C3	–		32 ± 2	>400	–	–
	C2	–		42 ± 3	No inhibition	–	–
	C6	–		50 ± 6	55 ± 3	–	–
	C4	–		57 ± 3	240 ± 40	–	–
	C7	–		115 ± 10	215 ± 20	–	–
	C8	–		330 ± 30	210 ± 20	–	–
^81^	Punicalagin	0.43 (WT) Two-fold reduction (E319A, E375A)		0.44 (WT) -		47 (A549-ACE2) 33 (VERO)	347 (A549-ACE2) 196 (VERO)
	Rhodiosin	0.48		–		–	–
	Punicalin	0.54		0.63		–	–
	Dyngo-4a	0.63		–		–	–
	Tannic acid	1.25		–		–	–
	(-)-Gallocatechin gallate	1.34		–		–	–
	Silver sulfadiazine	2.11		–		–	–
	MC-Val-Cit-PABC-PNP	2.45		–		–	–
	Ellagic acid	2.8		Weak inhibition		–	–
	Zinc Orotate	4.12		–		–	–
	UMI-77	4.38		–		–	–
	Katacine	5.98		–		–	–
	Rosmanol	8.93		–		–	–
^82^	SSYA10-001	2.3 ± 0.7		–		–	–
	PF-03715455	3.02 ± 0.21		9.26 ± 1.93		–	–
	Licoflavone C	8.7 ± 1.3		20.9 ± 0.8		–	–
	PF-00610355	22.4 ± 1.3		>30		–	–
	Ceftaroline fosamil	>30		>30		–	–
	NADH	>30		>30		–	–
	Polydatin	>30		>30		–	–

a See the footnote of Table 1.

b See the footnote of Table 1.

c See the footnote of Table 1.

d See the footnote of Table 1.

e Assay conditions: A (180 nM dsDNA, 0.1 mM ATP, 1.5 nM nsp13), B (180 nM dsRNA, 2 mM ATP, 1 nM nsp13), C (180 nM dsDNA, 0.1 mM ATP, 0.5 nM), D (180 nM dsRNA 0.1 mM ATP, 1 nM nsp13).

### 3.3 Helicase kinetic properties

Only ten studies reported the kinetic parameters of the enzymes used in the assays.^26^^,^^27^^,^^56^^,^^64^^,^^72^^,^^73^^,^^77^, ^78^, ^79^, ^80^ These studies estimated the Michaelis-Menten constants (*k*_cat_) and turnover numbers (*K*_m_) of the ATP and double-stranded nucleic acid substrates of the tested helicases. Kinetic parameters of the double-stranded nucleic acid are estimated by measuring the rate of Forster resonance energy transfer (FRET) signal increase as a result of the separation of fluorophore-labeled double-stranded nucleic acids (Table S2). MERS-CoV and SARS-CoV-2 helicases were reported to similarly unwind dsDNA and dsRNA.^26^^,^^27^^,^^77^ In addition, the unwinding activity of the SARS-CoV and SARS-CoV-2 helicases was found to depend on the presence of a single-stranded overhanging sequence at the 5’ end.^64^^,^^72^ Kinetic parameters of the ATP substrate were estimated either by measuring the rate of phosphate release with different inhibitor concentrations^55^, ^56^, ^57^, ^58^, ^59^, ^60^, ^61^, ^62^^,^^64^, ^65^, ^66^, ^67^, ^68^, ^69^, ^70^, ^71^, ^72^, ^73^^,^^75^^,^^79^ (Table S2). Several studies have also estimated ATP substrate kinetic parameters by measuring the rate of FRET signal increase as a result of labeled double-strand nucleic acid separation.^26^^,^^27^^,^^64^^,^^77^^,^^78^ In the presence of single-stranded polynucleotides, ATPase activity of SARS-CoV and SARS-CoV-2 helicases surged, and the ATP substrate kinetic parameters changed significantly.^56^^,^^78^ One study showed that SARS-CoV-2 can hydrolyze any of the four natural nucleoside triphosphates (NTPs), with the greatest preference for ATP. This study also showed a preference of the ATPase activity of the SARS-CoV-2 helicase for Mg^2+^ over other divalent metallic ions. However, high Mg^2+^ concentrations were found to be inhibitory. Similar divalent ion preferences were observed for SARS-CoV-2 helicase unwinding activity.^72^

### 3.4 Tested compounds characteristics

**Fig. 3:**
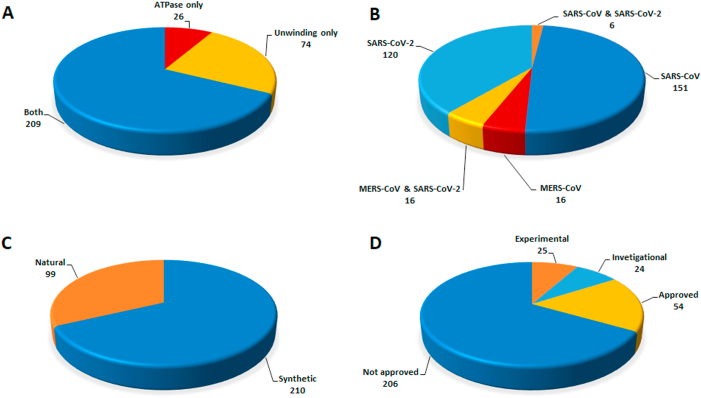
Classes of tested compounds by: (A) enzymatic activity, (B) virus, (C) nature of compound, and (D) phase of clinical approval.

**Fig. 4:**
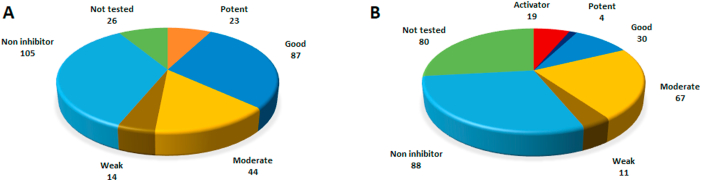
Inhibition potency of tested compounds by: (A) unwinding activity and (B) and ATPase activity.

**Fig. 5:**
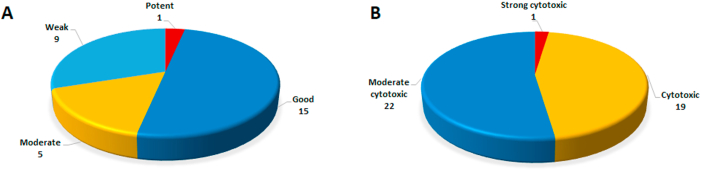
Cellular activities of potent unwinding and ATPase inhibitors: (A) antiviral activity and (B) cytotoxicity.

The inhibitory effect of the reported compounds was mostly measured against two types of coronavirus helicases involving unwinding and the ATPase activity. A lesser number were tested against a single activity (Table 1, Table 2, Table 3). Reported compounds were mainly tested against SARS-CoV and SARS-CoV-2 helicases, and fewer compounds against MERS-CoV helicase A limited number of compounds were tested against more coronavirus helicases (Table 1, Table 2, Table 3). Two thirds of the tested compounds were synthetic, while the remaining ones were natural products (Fig. 3A, Table S5). According to the DrugBank database, most tested compounds were either not approved or had not gone through approval procedures.^86^ Few of these compounds were approved pharmaceuticals or the subject of ongoing experimental and clinical investigations (Fig. 3B). Using the previously established criteria for categorizing the *in vitro* anti-plasmodial activity of extracts and compounds^87^, the IC_50_ of tested compounds were categorized into five ranges: potent inhibitors (IC50 < 1 μM), good inhibitors (1 ≤ IC50 < 20 μM), moderate inhibitors (20 ≤ IC50 < 100 μM), weak inhibitors (100 ≤ IC50 < 200 μM), and non-inhibitors (IC50 > 200 μM). The majority of were either weak inhibitors or non-inhibitors. This was the case for both unwinding and ATPase. Aptamers are a unique class of tested compounds. They work as unwinding activity inhibitors; however, they activate ATPase activity (Fig. 4A,B). The efficiency of infection inhibition and cytotoxicity were categorized in accordance with previously published ranges of herbal extract effects on hepatoma cell lines.^88^ Nineteen helicase inhibitors were selected for VERO cell infection inhibition and cytotoxicity tests. Seventeen inhibitors showed moderate cytotoxic effects. The cytotoxicity of the remaining inhibitors was classified as toxic since they were only tested at a low concentration (Fig. 4, Fig. 5B).

### 3.5 Tested coronavirus helicases

#### 3.5.1 Compo*unds tested against SARS*-CoV helicase

Several studies have investigated the effect of previously identified antiviral compounds on the SARS-CoV helicase activities.^56^, ^57^, ^58^^,^^61^, ^62^, ^63^ Adamantane derivatives, including vanillinbananin, bananin, eubananin, and iodobananin, inhibited unwinding and ATPase activities of the SARS-CoV helicase at low molar concentrations. Ansabananin and adeninonananin did not inhibit either of these two activities. Bananin inhibited both activities in a non-competitive manner, indicating possible allosteric interferences with the nucleotide and nucleic acid binding sites Table S3. Furthermore, bananin inhibited VERO cell infection at lower concentrations that were not cytotoxic to cells.^56^ Fifteen bismuth ion-based compounds were tested against the SARA-CoA helicase^57^^,^^58^ (Table 1). Ranitidine bismuth citrate (RBC), effectively inhibited the unwinding and ATPase activities of SARS-CoV at low micromolar concentrations. The IC_50_ of RBC inhibition of the ATPase activity of SARS-CoV was not affected by the presence or type of the second substrate, the oligonucleotide. There was little variation between the IC_50_ of RBC inhibition measured by SDS-PAGE-based and that measured by FRET-based assays. RBC inhibited FRhK-4 cell infection with SARS-CoV and reduced viral RNA in infected cells in a concentration- and time-dependent manner. The compound RBC cytotoxicity of FRhK-4 cells was relatively low. Compared to RBC, other bismuth-based compounds, including the bismuth tricysteine complex, bismuth nitrate, and bismuth nitrilotriacetate, showed less inhibitory effects against SARS-CoV ATPase activity. Bismuth acetohydroxamate inhibited the ATPase activity of SARS-CoV at relatively high concentrations, and bismuth ethylenediaminetetraacetate did not inhibit ATPase activity. None of these compounds, except RBC, were tested against the unwinding activity of the SARS-CoV helicase.^58^ Derivatives of bismuth complexes containing N- and O-based ligands were found to be effective inhibitors of SARS-CoV helicase unwinding and ATPase activities. At sub-molar concentrations, Complexes 1 and 2 inhibited the ATPase activity. None of these derivatives were tested against SARS-CoV infected cells.^57^ Aryl diketoacid derivatives selectively inhibited the unwinding activity of the SARS-CoV helicase with no effect on ATPase activity. These derivatives consist of a central diketoacid core attached to an arylmethyl group. The most potent inhibition was produced by derivatives with the substitutions R2 = R4 = H and R3 = NHCH2-phenol or R3 = NHCH2 (4-Cl-phenol).^61^ Dihydroxychromones, a naturally occurring bioisostere of aryl diketoacid, also inhibited SARS-CoV helicase activity. Derivatives with substitutions R^1^ = R^2^ = H and R^3^ = 3-CN-Benzyl or R^3^ = 4-Cl-Benzyl were the most potent inhibitors.^62^ In contrast to the dihydroxychromone and aryl diketoacid derivatives reported in the previous study, the 2,6-Bis-arylmethyloxy-5-hydroxychromone derivatives, which consist of a dihydroxychromone attached to two arylmethyl groups, inhibited the SARS-coV helicase unwinding and ATPase activities. Derivatives with iodo substitutes in the R group exhibited the most potent inhibition.^63^

A second group of studies have used high-throughput methods to screen compound libraries for SARS-CoV helicase activities.^55^^,^^64^, ^65^, ^66^, ^67^, ^68^, ^69^, ^70^ Many synthetic compounds were identified as SARS-CoV helicase inhibitors. The synthetic compound HE602 inhibited the SAR-CoV helicase unwinding activity and polynucleotide-stimulated ATPase activity at a micromolar concentration. It also protected VERO cells against SARS-CoV infection at micromolar concentrations. However, its cytotoxic effects were not reported.^55^ The compounds EMMDPD, DTPMPA, and FSPA inhibited the unwinding and ATPase activities of the SARS-CoV helicase; however, their inhibitory effect was more potent against the ATPase activity. They also showed no cytotoxic effects on WI-38 cells at concentrations up to 40–80 μM.^68^, ^69^, ^70^ The inhibitors SSYA10-002 and SSYA10-002 were also identified as a result of high-throughput screening against the SARS-CoV unwinding activity. Kinetic studies and molecular docking were both used to predict the binding site of the inhibitory compound SSYA10-001 showing that SSYA10-001 inhibited the unwinding activity of the SARS-CoV helicase in a non-competitive manner versus both substrates, dsDNA and ATP. However, it had no inhibitory effect on the ATPase activity^64^ (Table S3). Virtual prediction of the binding pocket followed by virtual docking positioned SSYA10-001 within a specific putative binding site with possible interactions with several residues, including Y277, R507, and K508. Moreover, mutating Y277 and K508 residues to alanine alleviated the SSYA10-001 inhibitory effect.^65^ High throughput screening of natural compounds led to the identification of several ATPase inhibitors of the SARS-CoV helicase, including scutellarein, myricetin, myricitrin, amentoflavone, diosmetin-7-O-Glc-Xy, and taraxerol. Only scutellarein and myricetin were potent inhibitors. Docking of scutellarein and myricetin within the nucleotide binding site of the SARS-CoV helicase revealed that they fitted very well within the site. None of the reported natural products inhibited the unwinding activity, even at 10 μM concentrations.^66^ In another study, baicalein and, to a lesser extent luteolin and oroxylin A, inhibited the ATPase activity of the SARS-CoV helicase but did not inhibit the unwinding activity, even at 10 μM concentrations^67^

A third group of studies used the systematic evolution of ligands by exponential enrichment (SELEX) to identify RNA ligands (aptamers) that can potentially bind to the SARS-CoV helicase with high specificity and thus inhibit its activity^59^^,^^60^ (Table 1). Fifteen rounds of SELEX cycles from a random library of 40-nt RNA oligonucleotides identified six aptamers as SARS-CoV helicase binders. These aptamers contain an AG-rich conserved sequence, mostly residing in a loop motif. In the absence of the second substrate mimic, a pool of these aptamers inhibited the unwinding activity of the SARS-CoV helicase by 85% with an IC50 at nanomolar concentrations which was significantly alleviated when the second substrate mimic was added to the reaction. In the second study, two types of aptamers with distinct secondary structures were shown to bind to the SARS-CoV helicase: the G-quadruplex and the non-G-quadruplex aptamers. Only the non-G-quadruplex aptamers significantly inhibited the unwinding activity of the SARS-CoV helicase at nanomolar concentrations. Capping the 3′-end of the aptamer with biotin or inverted thymidine increased the stability of the aptamers and enhanced their inhibition potency. The non-G-quadruplex aptamers activated the ATPase activity, as shown by significantly decreased apparent Michaelis-Menten constants (Table 1. To explain the simultaneous inhibition of unwinding activity and activation of ATPase activity, it is suggested that these aptamers bind to the nucleic acid binding site, causing the enzyme to be “locked” in a high ATPase turnover conformation^60^ which is supported by the fact that the ES15 aptamer loses its ability to activate the ATPase activity in the presence of the pol (rU) substrate mimic. Under these conditions, the nucleic acid binding site is occupied by the substrate mimic and the enzyme is not “locked” in a high ATPase activity conformation^59^

#### 3.5.2 Compounds tested against the MERS-CoV helicase

Triazole derivatives were found to inhibit the ATPase and unwinding activities of MERS-CoV helicase^71^ (Table 2). Derivatives with cyclopentenyl moiety were the most effective inhibitors of both enzyme activities. High-throughput screening of a library of FDA-approved compounds revealed several potential inhibitors of the unwinding activity of the MERS-CoV helicase.^27^ Many potential inhibitors, including epirubicin HCl, doxorubicin HCl, daunorubicin HCl, mitoxantrone 2HCl, and idarubicin HCl, were found to dock well in the nucleotide binding site of the MERS-CoV helicase. Their inhibitory effect could be attributed to their interaction with several residues, including Q404, R442, R443, and R567, which are essential for the stabilization of a SO_4_^2−^ ion located within the nucleotide binding pocket of the enzyme.^14^ Other weaker inhibitors of the unwinding activity of the MERS-CoV helicase were identified by high-throughput screening methods. These include ethacridine lactate monohydrate, otilonium bromide, tolcapone, and diminazene aceturate.^27^ In another screening study, clofazimine was found to inhibit the unwinding activity of MERS-CoV helicase and inhibit VERO cell infection by MERS-CoV.^74^ Virtual prediction of binding sites followed by docking of possible inhibitors was also used to show that the compound SSYA10-001 could possibly interact with a specific putative binding site within the MERS-CoV helicase. Virtual docking showed that SSYA10-001 interacts with residues Y277, S507, and K508 of the MERS-CoV helicase, which are the corresponding residues of Y277, R507, and K508 in the SARS-CoV helicase.^65^

#### 3.5.3 Compounds tested against the SARS-CoV-2 helicase

Bismuth ion-based compounds were also tested against the ATPase and unwinding activities of the SARS-CoV-2 helicase^72^^,^^73^ (Table 3). At low micromolar concentrations, RBC inhibited both activities. RBC increased the apparent Michaelis-Menten constants of the nucleic acid and ATP substrates, however, it did not affect the maximum velocity of either of the two activities. The data was fitted into a competitive inhibition model, and inhibition constants were calculated (Table S3).^73^ When a bismuth-bound SARS-CoV-2 helicase was supplemented with 50 molar equivalents of Zn^2+^, only 6% of ATPase and 13% of unwinding activities were regained, indicating that the inhibition is irreversible since Zn^2+^ ions are unable to compete with Bi^3+^ ions out of ZBD. RBC inhibited VERO and Caco2 cell infections with SRAS-CoV-2. RBC showed low cytotoxic effects on both types of cells. Similarly, colloidal bismuth subcitrate (CBS) inhibited both activities of the SARS-CoV-2 helicase, but to a lesser extent than RBC.^72^^,^^73^ CBS protected the SARS-CoV-2-infected VERO and Caco2 cells at low concentrations while causing little toxicity. In the same study, the porphyrin-based bismuth derivatives Bi(TPyP) and Bi(TPP) were found to be effective inhibitors of SARS-CoV-2 helicase two activities. At low micromolar concentrations, Bi(TPyP) and Bi(TPP) effectively protected VERO and Caco2 cells from infection; however, their toxicity was higher than that of RBC and CBS.^73^ Bismuth citrate was discovered to be a weak inhibitor of SARS-CoV-2 helicase activity.^72^^,^^73^ Recently, it was reported that Zn^2+^-ejecting agents, ebselen and disulfiram, can effectively inhibit the ATPase activity of the SARS-CoV-2 helicase at sub-micromolar concentrations. At a concentration of 25 M, both compounds inhibited 50% of VERO cell infection. However, when used in combination with 5 μM remdesivir, ebselen and disulfiram protection were raised to 99% and 91%, respectively^75^ In another recent study, several 2- phenylquinoline derivatives were found to inhibit unwinding and ATPase activities of SARS-CoV-2 helicase. Few of these compounds exhibited good antiviral properties when tested by VERO cells models.^79^

High-throughput screening of natural compounds identified several flavonoids as inhibitors of the unwinding activity of the SARS-CoV-2 helicase. Licoflavone C behaved as a non-competitive inhibitor of unwinding and ATPase activities with respect to dsDNA and ATP. Flavanone and kaempferol also showed non-competitive inhibition of unwinding activity with respect to ATP (Table S3). Docking of these flavonoids within the vicinityof the SARS-CoV-2 helicase showed that licoflavone C docked well at nucleic acid and nucleotide binding sites. Other flavonoids were more effective at docking at the nucleic acid binding site. Flavonoid inhibition was alleviated by the addition of BSA/TCEP, which could be explained by the non-specific and competitive binding of inhibitors to BSA or by the increased stability of the enzyme. The improved kinetic properties of the enzyme in the presence of BSA/TCEP support the second possibility.^78^ Another high throughput screening study showed that several suramin-related compounds, including suramin, NF023, Evans blue, PPNDS, and diphenyl blue, were identified by high-throughput screening methods as potent inhibitors of the unwinding activity of the SARS-CoV-2 helicase. The PDK1/Akt/Flt dual pathway inhibitor and FPA-124 were also identified as potent inhibitors. In addition, many other compounds were identified as good inhibitors of the enzyme. However, their inhibitory effects were significantly abolished with the use of detergents. The authors concluded that the inhibitory effect of these compounds was due to an aggregation effect rather than true inhibition. This conclusion was supported by the fact that the inhibitory effect previously mentioned was not altered by detergent addition. The same study has investigated the cytotoxic and infection-protective effects of suramin, FPA-124, SSYA10-001, and myricetin. All of these molecules have shown good infection protection potency while maintaining a low cytotoxic activity on VERO cells.^77^ High-throughput screening of kinase inhibitors has found several inhibitors of the SARS-CoV-2 helicase. Two of these inhibitors, C1 and C2 inhibit the ssDNA activated SARS-CoV-2 helicase. The remaining derivatives inhibit the ATPase activity in the absence of ssDNA at various levels. The antiviral and cytotoxic activity of these compounds has not been reported.^80^

Another approach of high throughput screenings was also used to identify SARS-CoV-2 helicase inhibitors. Compound libraries were tested for their SARS-CoV-2 replication inhibitory effects. Positive hits were then tested against the helicase activity. Screening of FDA-approved drug library identified clofazimine as a SARS-CoV-2 replication inhibitor. Clofazimine is shown to inhibit SARS-CoV-2 unwinding activity and also VERO cell infection.^74^ In a similar approach, xanthorrhizol was shown to protect VERO cells from SARS-CoV-2 infection with low toxicity, however, when tested against the helicase, it caused no inhibition of the unwinding activity. Its effect on ATPase was not tested.^76^ Previously identified MERS-CoV helicase FDA-approved inhibitors were found to similarly inhibit the unwinding activity of SARS-CoV-2 helicase, including epirubicin HCl, doxorubicin HCl, daunorubicin HCl, mitoxantrone 2HCl, idarubicin HCl, and ethacridine lactate monohydrate. However, several MERS-CoV helicase inhibitors were not inhibitory to the SARS-CoV-2 helicase, including otilonium bromide, tolcapone, and diminazene aceturate.^26^^,^^27^

Virtual screening of FDA-approved compounds containing a sulfate moiety docked within the nucleotide-binding site of SARS-CoV-2 helicase has identified several compounds, including zafirlukast, montelukast, darunavir, mezlocillin sodium, bosentan hydrate, lapatinib, sivelestat sodium tetrahydrate, omipaslisib (GSK458), tianeptine, and pazopanib, as potential inhibitors of the enzyme. A FRET-based assay confirmed the inhibitory effect of zafirlukast, a leukotriene receptor antagonist used to treat chronic asthma. The remaining compounds did not inhibit the enzyme. The docking score of montelukast, another leukotriene receptor antagonist, was low. However, montelukast did not inhibit the enzyme. Zafilrukast inhibited VERO cell infection with SARS-CoV-2 at 25 μM as indicated by the increased number of threshold cycles to propagate viral RNA inside VERO cells. No cytotoxic effects of zafirlukast were observed at this concentration.^26^ In another virtual screening study, lumacaftor and cepharanthine were identified as possible binders of the nucleotide binding site of the SARS-CoV-2 helicase. Lumacaftor and cepharanthine inhibited the ATPase activity of SARS-CoV-2; however, they were not tested against the unwinding activity.^33^ A third virtual screening of FDA-approved and bioactive compounds (TopScience) lead to the identification of several potential SARS-CoV-2 helicase inhibitors including: punicalagin, rhodiosin, dyngo-4a, tannic acid, (-)-gallocatechin gallate, silver sulfadiazine, MC-Val-Cit-PABC-PNP, zinc orotate, UMI-77, katacine, and rosmanol. Punicalagin and its analogs punicalin and ellagic acid showed the most inhibitory effects against unwinding activity. Punicalagin and punicalin inhibited the ATPase activity potently, however, ellagic acid did not. Punicalagin showed good antiviral activity while maintaining low cytotoxicity toward A549-ACE2 and VERO cells.^81^ Another recent virtual screening study of the FDA-approved drug library identified five unwinding and ATPase inhibitors of the SARS-CoV-2 helicase. These compounds belong to various chemical groups including the beta-lactam derivative ceftaroline fosamil, the glicoside derivative polydatin, the purine analog NADH, the acetamide derivative PF-00610355, and the triazole derivative PF-03715455. PF-03715455 showed the most inhibitory effect on unwinding and ATPase activities. The antiviral activities of these compound were not tested.^82^

## 4 Discussion

This review is the first systematic one that has collected and combined all the compounds that were tested against the helicases of SARS-CoV, MERS-CoV, and SARS-CoV-2 by *in vitro* methods and provides a comprehensive and reliable source for future drug discovery efforts. Drug repurposing studies can significantly benefit from knowing whether a drug has been tested against the coronavirus helicase or not. On the other hand, drug design studies can benefit from mapping the possible inhibitory interactions between the enzyme binding sites and various inhibitors. Many of the reported inhibitors can be a good starting point for the development of more potent inhibitors. Based on their inhibition potency, VERO cell protection, and VERO cell cytotoxicity, a list of helicase inhibitors with potential antiviral properties was compiled. Many on this list could potentially be used for further animal models and clinical studies.

### 4.1 Screening strategies

In response to the COVID-19 pandemic, drug discovery efforts were focused on targeting essential viral replication proteins and enzymes. Enzymes including 3-chymotrypsin-like protease (3CLpro) and RNA-dependent RNA polymerase (RdRp) were targeted by new inhibitors. These could be potentially used to prevent SARS-CoV-2 infection and alleviate symptoms.^89^ Other enzymes, despite their essential roles in viral replication, like papain-like protease (PLpro), exoribonuclease (nsp14) and helicase (nsp13), have received relatively less attention as possible targets.^90^ From the first SARS-CoV outbreak in 2003 up to the 2019 SARS-CoV-2 pandemic, few drug discovery studies were designed to target the coronavirus RNA helicase (nsp13), an essential component of the replication-transcription complex (RTC), responsible for the viral gene replication process. A significant number of these studies were virtual. However, only a few used *in vitro* assay methods to directly test the compound inhibitory effects. While virtual screening is easy, fast, and cost-effective, it is inherently limited by the computational capabilities of the program used.^91^ Therefore, virtual studies cannot replace *in vitro* and *in vivo* assays but can be used as a complementary method that speeds up the discovery process.^92^ The objective of this systematic review was to evaluate the inhibitory effects of the compounds tested by *in vitro* methods against coronavirus helicase activities.

The inclusion and exclusion criteria were defined and applied to the outcome of the initial search of the online databases. A final list of 29 studies was used for the data extraction. Included studies were published between 2004 and 2022. Dates of publication were associated with the three major outbreaks of pathogenic coronaviruses. This association clearly shows how the drug discovery research community actively responded to these events, which eventually led to the discovery of several novel inhibitors. These studies examined the inhibitory effects of 309 compounds on helicase activities of one or more of the three major pathogenic coronaviruses. Most of the reported compounds were directly identified by *in vitro* high-throughput screening of commercial or in-house compound libraries. Several compounds were first predicted by virtual screening as possible binders, and then their inhibitory effects were confirmed by *in vitro* assays. Other compounds were previously characterized inhibitors of non-coronavirus helicases or other viral enzymes that were tested against coronavirus helicase activities. A special group were aptamer-based inhibitors that were isolated by the SELEX method and tested against helicase activities. Several inhibitors showed good antiviral activity and low toxicity characteristics, which strongly underlines their potential. Despite these findings, no clinical trials were done to investigate these inhibitors as therapeutic antiviral agents, particularly in the period post-SARS-CoV and MERS-CoV epidemics. The lack of interest in moving forward with these coronavirus helicase inhibitors to clinical phases could be attributed to the view of these outbreaks as short-duration epidemics with limited financial incentives that cannot compensate for the high cost of drug development.^93^ The latest COVID-19 pandemic dramatically changed this view towards coronavirus outbreaks, which are expected to continue to threaten the human population for longer periods of time.

### 4.2 Helicase assays conditions

The inhibitory effects of the compounds studied on helicase were measured using one of three assays: ATP hydrolysis, gel electrophoresis, or FRET-based solution assay. Despite its relatively high running cost^94^, most studies in this review used the FRET-based solution assay as their principal screening method. The most significant benefit is that it is a continuous assay that measures the separation of dsDNA or dsRNA into single, separated strands in real time. Generally, continuous assays are usually more sensitive and accurate than end-point assays.^95^ Separation of dsDNA or dsRNA after helicase treatment can also be directly monitored by electrophoresis after exposure to the enzyme; however, this assay is an end-point assay with limited capacity to test a large number of compounds. This could explain why fewer studies in this review adapted gel electrophoresis as a screening method. The ATPase assay is an end-point colorimetric assay that measures the released inorganic phosphate as a result of ATP hydrolysis. Even though it is an end-point assay and does not measure the unwinding activity directly and is not necessarily selective for inorganic phosphate in the presence of labile organic phosphate compounds, many studies in this review have screened their compounds against the ATPase activity since it is adaptable to high throughput screening methods and less expensive compared to the FRET-based assay.^96^ However, the majority of these studies used FRET-based assays to confirm the inhibitory effects of potential compounds, as it is best to measure both activities under identical conditions to ensure reproducibility.^94^

The included studies in this review showed considerable variation in the reported values. The kinetic parameters of the ATP substrate determined by the ATP hydrolysis or ATP luciferase-dependent assay had a wider variation range than those determined by the unwinding assay. The variations of V_max_ for the ATP substrate were high, whether measured by ATP hydrolysis or unwinding activity. It is worth noting that many studies reported their V_max_ values as arbitrary unit changes per time rather than changes in concentration per time, which makes it difficult to compare with other studies. In a similar fashion, the kinetic parameters of dsDNA also varied between different studies. Some studies reported their V_max_ values in arbitrary units. Many of these variations can be attributed to the variations in assay conditions, including enzyme concentration and type, double strand substrate length and type, ATP concentration, and buffer conditions. The variations of kinetic parameters obtained by ATP hydrolysis and luciferase-based assays were generally wider compared to those obtained by unwinding assays. This could be explained since both ATP assays are end-point assays and highly affected by assay conditions (Tables S2 and S4). Despite the variations in kinetic parameters of helicases, other properties were consistent with previously published properties of these enzymes. The preference of SARS-CoV-2 for the ATP substrate over other nucleotides is consistent with other published studies showing that SARS-CoV helicase can hydrolyze any of the eight natural NTPs and dNTPs, with a preference for ATP or dATP as demonstrated by the ratios.^83^^,^^84^ Similar preferences were reported for the MERS-CoV helicase.^97^ The SARS-CoV-2 helicase dependence on divalent ions is confirmed by published studies. The stimulation of SARS-CoV and SARS-CoV-2 ATPase by single-stranded polynucleotides is also supported.^97^^,^^98^ Some studies in this review, as previously mentioned, have reported the ability of MERS-CoV and SARS-CoV-2 helicases to unwind dsRNA and dsDNA equivalently. Several previously published studies have reported a similar behavior of the SARS-CoV.^83^, ^84^, ^85^ The unwinding activity of the SARS-CoV helicase depends on the presence of a single-stranded overhanging sequence at the 5′ end. This indicates that the unwinding activity moves with a 5′ to 3’ polarity.^83^^,^^85^ The SARS-CoV helicase unwinds the nucleic acid in a discrete, stepwise fashion, with an estimate of about 9.5 base pairs (bp) being unwound in each step. With approximately 30 steps taking place each second, the catalytic rate of the reaction is estimated at 231 unwound bp per second. Additionally, it was found that SARS-CoV RNA-dependent RNA polymerase (nsp12) can increase the step size to about 17.1 bp and thus enhance the catalytic rate to 538 unwound bp per second.^85^

### 4.3 Inhibitory interactions

Helicase inhibitors can be divided into several categories according to the site of their interaction: zinc-binding site inhibitors, nucleic acid binding site inhibitors, and nucleotide binding site inhibitors. A fourth category can be added for compounds that inhibit the enzyme but provide no clear evidence of where they bind onto the enzyme. Inhibitors that have been shown to interact with more than one binding site can be classified into several categories.

#### 4.3.1 Zinc-binding site interactions

Structural studies have shown that the metal-binding domain of the SARS-CoV helicase contains three cysteine-rich zinc fingers. It has been suggested that bound Zn^2+^ ions are essential for the coronavirus helicase activity, and that their release could possibly affect the enzyme's functionality.^15^ The addition of Zn^2+^-ejecting agents like ebselen and disulfiram to the SARS-CoV-2 helicase resulted in a decrease in enzymatic activity and an increase of freely released Zn^2+^ ions, as indicated by the Zn^2+^-selective fluorescent indicator.^75^ In addition to Zn^2+^-ejecting agents, helicases could also be inhibited by cation-releasing compounds that can remove Zn^2+^ ions from the zinc-binding domain (ZBD) and thus inhibit the enzyme. Bismuth ion Bi^3+^ has previously been shown to form strong interactions with cystine thiolates in metallothionein (MT), copper ion Cu^2+^, and zinc ion Zn^2+^-binding proteins. The Bi^3+^ ion binding to metallothionein releases the originally bound ions and consequently impairs protein function^99^, ^100^, ^101^ The replacement of Zn^2+^ ions on cystine thiolates in the metal-binding domain in coronavirus helicases by Bi^3+^ ions is suggested as the possible mechanism of the inhibitory effect of the discovered bismuth ion-based inhibitors. This cation exchange mechanism is supported by titration experiments, which indicate that Zn^2+^ and Bi^3+^ ions bind to the ZBD of SARS-CoV with the same stoichiometry. When one Zn^2+^ ion-loaded ZBD was titrated with the chelating agent 4-(2-pyridylazo)resorcinol (PAR), a total of 2.8 ± 0.2 bound Zn2+ ions were released from the ZBD. This amount was estimated as the total bound Zn^2+^ per ZBD. An equivalent concentration of 2.8 ± 0.3 bound Bi^3+^ ions was released when one Bi^3+^ ion-loaded ZBD was titrated with the same chelating agent.^58^ The Bi^3+^ ion released from RBC can bind to the SARS-CoV-2 helicase with a *K*_d_ of 1.38 ± 0.05 μM as shown by titration of Zn^2+^ ion-stripped SARS-CoV-2 helicase with RBC. Equilibrium dialysis of Zn^2+^ ion-loaded ZBD followed by inductively coupled plasma mass spectrometry (ICP-MS) showed that ZBD is bound to nearly 3.46 molar equivalents of Zn^2+^ions. Titration experiments of ZBD with RBC followed by equilibrium dialysis and ICP-MS showed that a total of 2.9 molar equivalents of Zn^2+^ ions are released and replaced by 2.73 molar equivalents of Bi^3+^ ions.^72^ Apparently, the displacement of Zn^2+^ ions by Bi^3+^ ions depends on the coordination environment of the Bi^3+^ ion within the bismuth-based complexes. Compounds with an internal coordination that slowly release Bi^3+^ have a weaker ability to replace the Zn^2+^ bound to the SARS-CoV helicase and have a lower inhibition potency.^57^

Based on previous evidence of aryl diketo acids (ADK) inhibiting several viral enzymes like the HIV-1 integrase and hepatitis C RNA-dependent RNA polymerase (RdRp) by extracting metals away from the metal binding sites, it has been suggested that ADK could inhibit SARS-CoV helicase by removing the essential Zn^2+^ ions from the ZBD in a similar fashion to bismuth complexes.^61^ Unlike bismuth complexes, ADKs only inhibited the SARS-CoV helicase unwinding activity and had no or a minor effect on ATPase activity. This behavior made it difficult to conclude that the ADK is mimicking the bismuth complex's action. It was shown that derivatives with substitutions in the meta position of the aryl group caused the most potent inhibition of the unwinding activity. To further investigate the interaction of the ADK compounds with the SARS-CoV helicase, they extended their search by looking into the impact of changes of the ADK's inhibitory effect. Dihydroxychromone derivatives were investigated, and it was found that, similar to the original ADKs, they inhibited the unwinding activity of the SARS-CoV helicase, however, not the ATPase activity. Dihydroxychromone derivatives with a free catechol group and an arylmethyl group on the opposite side caused the most potent inhibition of the unwinding activity.^62^ Similarly, the 2,6-bis-arylmethyloxy-5-hydroxychromone derivatives of the ADK were only inhibiting the unwinding activity of the SARS-CoV helicase. The most potent inhibition was observed in derivatives with 2-arylmethyloxy moiety substituents.^63^

#### 4.3.2 Nucleic acid-binding site interactions

The ATPase function of the SARS-CoV helicase is activated by ES15 aptamers pooled in the absence of the RNA substrate. This implies that the aptamer binds as a substrate at the nucleic acid binding site, effectively locking the enzyme at the structural conformation of high ATP hydrolysis turnover. This is confirmed by the observation of the loss of this activation when aptamers were added to the enzyme in the presence of the RNA substrate. In this conformation, the nucleic acid-binding site is occupied by the RNA rather than the aptamer. Unwinding activity, on the other hand, is only inhibited by aptamers in the presence of the RNA substrate.^59^^,^^60^ Capping the potent inhibitors with a 3′-inverted thymidine or a 3′-biotin aptamer to protect aptamers from nuclease attack did not affect their inhibitory activity.^60^ Virtual docking models of several flavonoids, particularly licoflavone C, myricetin, and quercetin, indicate the presence of strong interactions between them and residues C309 and R560 in the nucleotide- binding site and residues H290, R442, and K569 in the nucleic acid-binding site of the SARS-CoV-2 helicase. These flavonoids, except licoflavone C, inhibited the unwinding activity of the SARS-CoV-2 without affecting the ATPase activity. This suggests that these flavonoids directly or indirectly interfere with the binding of the dsDNA substrate to the nucleic acid-binding site. Licoflavone C, on the other hand, inhibited both activities in a non-competitive manner. Combined with docking evidence, it is suggested that licoflavone could possibly bind to the nucleic acid-binding site, as shown by docking models, and cause indirect conformational changes in the nucleotide-binding site, as suggested by its non-competitive behavior. It is suggested that the binding of licoflavone C to the nucleic acid-binding site is strong and not affected by the binding of the dsDNA substrate to the same site, as indicated by similar inhibition levels of unwinding activity in the case of preincubating nsp13 for 10 min with licoflavone C or preincubating with the dsDNA substrate. On the other hand, inhibition by quercetin was significantly alleviated when the helicase was pre-incubated with dsDNA, which suggests that it has a less tight binding compared to licoflavone C.^78^ The inhibition of the unwinding activity of SARS-CoV-2 helicase by the heavily negative-charged suramin and suramin-based compounds, including NF023, Evans blue, PPNDS, and diphenyl blue, suggests that they inhibit the helicase by binding to a positively charged pocket within the helicase. Previous evidence of suramin inhibition of the unwinding activity of the NS3 protein of the dengue virus^102^, in addition to the structural evidence of suramin binding to positively charged nucleic acid-binding pockets of enzymes like cullin-RING E3 ubiquitin ligases^103^ and norovirus RdRp^104^^,^^105^ suggest that suramin is likely to bind to the nucleic acid-binding site of the SARS-CoV-2 helicase. Nevertheless, in the absence of enough data, the possibility of suramin binding to the nucleotide-binding site cannot be completely excluded. A recent study of 2- phenylquinoline derivatives has revealed that some of these derivatives are portent inhibitors of the unwinding activity of the SARS-CoV-2 helicase, but showed a weak effect on the ATPase activity. These results suggest that these inhibitors are directly or indirectly interfering with the binding of the nucleic acid substrate.^79^

#### 4.3.3 Nucleotide-binding site interactions

Various approaches were used in different studies to provide evidence of the interaction between the inhibitor of interest and the nucleotide-binding site. The compound HE602 is shown to inhibit the ATPase activity of SARS-CoV, which is pre-activated by dT24 oligo. The same compound did not inhibit the unwinding activity of the enzyme, which suggests an interference with nucleotide binding rather than nucleic acid-binding.^55^ Similarly, banananins inhibited the ATPase activity of the SARS-CoV helicase when the enzyme was pre-activated by an dT24 oligo, however, they inhibited the unwinding activity in a less potent manner. This inhibition mode, plus the evidence of bananins inhibiting the ATPase activity in a non-competitive manner with respect to ATP and dT24, suggests that bananins are indirectly affecting the nucleotide- rather than the nucleic acid-binding site.^56^ Previous crystal structures of MERS-CoV has revealed the presence of sulfate, which serves as a precipitant in crystallization conditions, in the nucleotide-binding site pocket. By interacting with residues Q404, R442, R443, and R567, the sulfate moiety mimicked the interactions of the phosphate moiety of NTPs in the nucleotide-binding site.^14^ *In vitro* screening against the unwinding activity of the MERS-CoV helicase followed by virtual docking of the most potent inhibitors to the nucleotide-binding site has revealed that these inhibitors interact with one or more of the residues essential for phosphate moiety stabilization.^27^ Virtual docking of FDA-approved compounds that contain a sulfate moiety within the nucleotide-binding site of the SARS-CoV-2 helicase has identified zafirlukast as an inhibitor of the unwinding activity. The sulfate moiety of zafirlukast is shown to interact with two essential residues for phosphate moiety stabilization, R443 and the R567.^26^ It is important to point out that this line of evidence indicates possible strong interactions between the different inhibitors and the nucleotide-binding site; however, it does not exclude the possibility of the binding of these molecules to the nucleic acid-binding site or any other binding site on the enzyme, especially in the absence of docking experiments of these compounds within these sites or competitive inhibition evidence. Therefore, their classification as nucleotide-binding site inhibitors is loosely based on their strong interactions with residues required for the phosphate moiety stabilization, that is most likely present in the nucleotide-binding site rather than the nucleic acid-binding site. In a recent study, several kinase inhibitors (C3–C8) were found to inhibit the ssDNA-stimulated and unstimulated ATPase activity of the SARS-CoV-2 helicase.^80^ The ability of these compounds to inhibit the stimulated and unstimulated ATPase activity implies that they interfere with the binding of the nucleotide substrate. Two additional kinase inhibitors, C1 and C2, inhibited the ssDNA-stimulated ATPase activity of the SARS-CoV-2 helicase only. In the presence of the nucleic acid substrate in the nucleic acid-binding site, the interference of these inhibitors with nucleotide substrate binding is even more pronounced. However, in the absence of inhibition mechanism studies, unwinding activity measurement, or docking evidence this assumption remains open for further investigation.

Despite their structural similarities, flavonoids, a group of natural compounds made of a basic skeleton with varying attached functional groups^106^, seem to interact with the coronavirus helicases differently. As previously discussed, some flavonoids, including myricetin, possibly interact with the nucleic acid- binding site of SARS-CoV-2, thus inhibiting its unwinding activity but with no effect on the ATPase activity.^78^ However, in a different study, scutellarein and myricetin were found to inhibit the ATPase activity of the SARS-CoV helicase but not the unwinding activity. This suggests that they interact with the nucleotide-binding site rather than the nucleic acid-binding site. This conclusion was confirmed by a three-dimensional model that showed interactions between myricetin and several residues essential for NTP binding, including N265, Y269, and R443.^66^ It is worth mentioning that these studies have tested the inhibitory effects of myricetin against different helicase types and used different dsDNA substrates, however, the effect of these conditions on the inhibition model remains unclear and needs further investigation. Baicalein, also a flavonoid, inhibited the SARS-CoV helicase in a similar mode to scutellarein and myricetin, i.e., inhibition of the ATPase activity without affecting the unwinding activity. Despite the lack of docking evidence, it was suggested that baicalein binds to the nucleotide-binding site based on the structural similarities between baicalein and myricetin.^67^.

Punicalagin is a potent SARS-CoV-2 helicase inhibitor that binds directly to the nucleotide-binding site.^81^ A point mutation of the essential residues Glu-319 and Glu-375 to Ala dccreased punicalagin inhibition by two fold. Virtual docking showed that punicalagin docks at the interface between the 1A and 2A domains of nsp13 and interacts with several residues at the nucleotide-binding site. The affinity of punicalagin binding to the SARS-CoV-2 helicase was measured by surface plasmon resonance assay, and the K_d_ was determined at 21.62 nM. Despite the evidence that punicalagin prevents the formation of the helicase/DNA complex, virtual docking has revealed no overlap of punicalagin and the ssRNA/DNA- binding sites on the SARS-CoV-2 helicase. The authors have concluded that punicalagin prevents DNA binding allosterically.

#### 4.3.4 Unique binding sites interactions

Another group of studies have used solution experiments and virtual docking to show the binding of triazole derivatives to a unique binding site other than the known ones. Using pocket prediction programs followed by virtual docking, the SSYA10-001 compound was found to interact with several comparable residues that form a unique pocket in the SARS-CoV, MERS-CoV, and MHV helicases. This was further confirmed by the mutating residues Y277, R507, and K508 in the MERS-CoV helicase, which led to the knockout of SSYA10-001 inhibition. The non-competitive behavior of SSYA10-001 with respect to ATP and dsDNA also indicates that the inhibitor does not bind to either of the two binding sites but rather indirectly causes conformational changes that affect their function.^64^^,^^65^ Virtual docking of other triazole derivatives in the active site of the MERS-CoV helicase, despite their structural similarity to SSYA10-001, has revealed that they bind to a different site. They form strong interactions with the T159, T7, T171, and R163 residues in the 1B domain 71. Triazole derivatives seem to interfere with domain 1B, which with domains 1A and 2A plays an essential role in the stabilization of the single-stranded nucleic acid in the active site.^14^, ^15^, ^16^

#### 4.3.5 Indeterminate interactions

Several strong and most of the weak inhibitors reported in this review were tested against only one of the two activities of the coronavirus helicase.^26^^,^^27^^,^^33^^,^^54^^,^^63^^,^^74^, ^75^, ^76^, ^77^ This lack of data creates a gap in our understanding of how these inhibitors interact with the coronavirus helicases, which makes it difficult to predict their binding sites. This gap becomes even wider in the case of weak inhibitors. Although some of these studies did not provide sufficient evidence for where these inhibitors bind, they did provide information on where they do not bind in some cases. High docking scores of many of the tested weak inhibitors compared to strong ones in the nucleotide- binding site of MERS-CoV and SARS-CoV-2 helicases has indicated a lower possibility of binding of these molecules to the nucleotide-binding site.^26^^,^^27^ Obviously, the preceding remark does not rule out the possibility of these inhibitors binding to any of the defined binding sites, including the docked site, or other undefined sites on the enzyme. In another study, the triazole derivative PF-03715455 was shown to inhibit the ATPase and unwinding activities of SARS-CoV-2. Molecular docking analysis has indicated the ability of this inhibitor to bind to the nucleotide-binding site and the nucleic acid- binding site with close docking values (−6.42 kcal/mol and −6.82 kcal/mol, respectively). This does not rule out any of the binding sites or both as possible site of interaction. Similar conclusion can be drawn about the other reported inhibitors in this study.^82^

One study has raised the possibility of identifying false positive helicase inhibitors due to the formation of colloidal aggregations in the assay solution and consequently the binding of the helicase to these aggregates rather than a real inhibitory effect.^77^ Small molecules tend to aggregate in colloids when the concentration of these compounds exceeds their specific critical aggregation concentration (CAC), typically in the low-to-mid micromolar range. Each colloid has a diameter of several hundred nanometers and consists of approximately 10^8^ molecules. In contrast to precipitation, the colloidal concentration is very low in the femtomolar magnitude and thus cannot be seen visually. Proteins are strongly adsorbed to the surfaces of these colloids. This non-specific, yet strong, binding usually impairs the functionality of these proteins.^107^^,^^108^ In the previously mentioned study, many of the identified inhibitors of the unwinding activity of the SARS-CoV-2 helicase, such as myricetin, zafirlukast, and SSYA10-001, lost their inhibitory effect when tested in the presence of Triton-X-100, an anti-aggregation detergent. The detergent effect indicates that the inhibition of these molecules is likely due to the colloid interaction with helicases rather than a real inhibitory effect. However, due to the lack of exact estimation of the CAC of each of these compounds and of experimental evidence of colloid formation at the used concentration, such as dynamic light scattering or other methods used to identify colloidal aggregates, the authors have stressed that these compounds could be real inhibitors.^77^ Furthermore, the effect of detergents on the enzyme activity was not fully investigated, which makes it difficult to determine whether the loss of inhibition was due to dissolved colloid or the detergent interaction with the helicase itself. Another study has discussed the intercalation of the double-stranded nucleic acid substrate as a reason for the apparent inhibition of MERS-CoV and SARS-CoV-2 helicases by some compounds, including doxorubicin, epirubicin, daunorubicin, mitoxantrone, and idarubicin.^26^, ^27^ This argument is supported by the fact that the reported binding constants of these compounds to DNA are in agreement with their measured IC_50_.^89^, ^90^, ^91^, ^92^

### 4.4 Inhibitor cytotoxic and viral infection protection properties

**Fig. 6:**
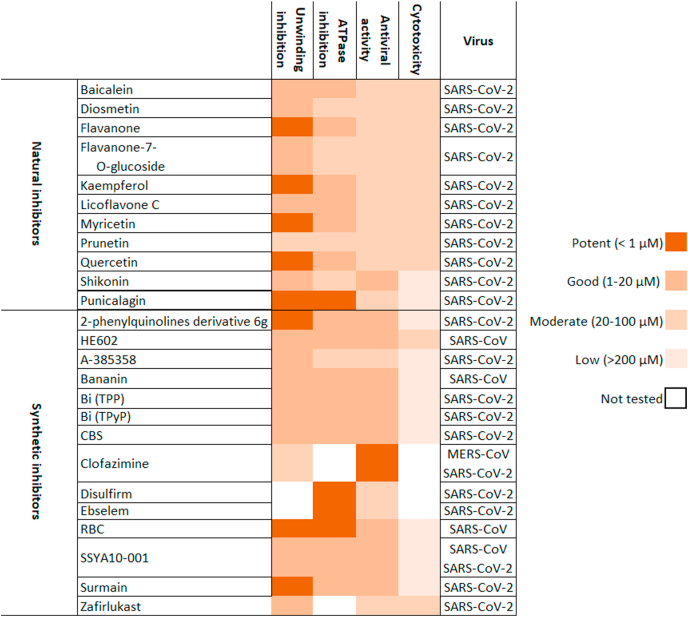
Heat map of List coronavirus helicase inhibitors with potential antiviral properties.

Most of the studies included in this review have investigated the cytotoxic and antiviral effects of potential coronavirus helicase inhibitors. Determining the cytotoxic and antiviral properties of the selected inhibitor is crucial for assessing whether it should undergo further clinical testing. Some studies did not measure the cytotoxic activity of the selected inhibitors since many are clinically approved and their cytotoxic activity is already known; however, these studies did not measure the antiviral activity of these compounds either. In one study, the antiviral effect was investigated using a SARS-CoV RNA replicon system.^64^ The RNA replicon system is an effective and safe system to investigate the viral replicative process, however, the poor stability of the RNA^108^ and the inability of the system to report all the interactions of the infection cycle^109^ make it a less reliable screening method compared to the VERO cell assay. The lack of cytotoxicity and antiviral activity data for some compounds has prevented further development of these compounds. A heat map of natural and synthetic compounds that have a complete dataset that includes enzymatic inhibition, cytotoxic effect, and antiviral activity could enable us to judge the suitability of these compounds as antiviral agents (Fig. 6). This heat map shows that synthetic inhibitors have lower cytotoxicity effects and better antiviral activity compared to natural inhibitors. These compounds could be advanced into further pre-clinical and clinical testing phases. None of these compounds has been investigated in a clinical setting, except for zafirlukast. In a recent publication, the efficacy of zafirlukast in hospitalized adult patients with moderate COVID-19 symptoms who were not admitted to an intensive care unit was assessed. The compound did not significantly improve symptom resolution. Because of the small sample size was, its apparent lack of efficacy as treatment for COVID-19 symptoms should be further tested with a larger sample size.^110^ The binding of these compounds to other sites could also be analyzed, and these compounds could be modified in accordance with their interactions with binding sites to improve their inhibitory properties.

### 4.5 Limitations

Although this systematic review has tried to be exhaustive, the probability of overlooking some tested compounds cannot be excluded due to the significant number of studies published since the SARS-CoV-2 outbreak. Compounds reported in non-English publications could be skipped in this review. In a recently published review paper,^111^ some of the cited references reported a few compounds tested against the SARS-CoV-2 helicase; however, these studies did not appear in our list of articles . This could be attributed to the fact that their titles and abstracts did not fit the inclusion criteria of this current systematic review. It is worth mentioning that most of the reported compounds in this group of excluded papers are already reported in other papers that are already included in the current review. Tested but not reported compounds could also limit the comprehensive nature of this review. Many of these compounds are items in large compound libraries that have been screened and found to inhibit the tested enzymes. One can argue that since these libraries have been identified, the included compounds have also been identified, and are indirectly included in this review. For that reason, the tested libraries are presented because of the comprehensive nature of this review (Table S4). The inconsistencies in the included study reporting of their findings limited the current review ability to fully compare the reported inhibitors. Many studies only reported an inhibitory effect against one of the helicase enzymatic activities, not both. On the other hand, many of these studies did not report enough solution or structural data to allow a better understanding of the mechanism of inhibition of the reported inhibitor and its possible interaction with the enzyme binding site. Several studies did not measure the cytotoxic or VERO protection parameters of the reported inhibitors. This limited the ability of this review to accurately conclude which inhibitors have better potential as antiviral agents. This became even more complicated since some studies have used different cell lines to evaluate cytotoxicity and viral infection protection.

## 5 Conclusions

This systematic review has shown *in vitro* screening of 309 compounds against the activities of SARS-CoV, MERS-CoV, and SARS-CoV-2 helicases. This study emphasizes the essential rule of *in vitro* methods despite their limitations when compared to fast and cost-effective virtual screening methods. It stresses the importance of using both virtual and *in vitro s*tudies as complementary approaches rather than as alternatives. Despite the differences in chemical properties of tested compounds, assay conditions, and enzyme properties, the structural and functional similarities of the three coronavirus helicase enzymes has allowed the classification of inhibitors according to the site of their interaction with the enzyme. This classification is useful for future structure-based drug design. Cytotoxicity and viral infection protection has enabled the compilation of a list of compounds that are shown to have moderate cytotoxic effects and good antiviral properties. These compounds could be good candidates for further clinical investigations.

## Declaration of competing interest

The authors declare that they have no known competing financial interests or personal relationships that could have appeared to influence the work reported in this paper.

## Data Availability

Data will be made available on request.
