# Supplementary material for: Coronaviruses SARS-CoV, MERS-CoV, and SARS-CoV-2 helicase inhibitors: a systematic review of invitro studies
Source: J Virus Erad. 2023 May 26;9(2):100327. doi: 10.1016/j.jve.2023.100327 (PMC10214743; doi:10.1016/j.jve.2023.100327)
Supplement: Multimedia component 1 [file mmc1.pdf]

Supplementary Material

Coronaviruses SARS-CoV, MERS-CoV, and SARS-CoV-2 helicase inhibitors: a systematic review of in vitro studies

Nimer Mehyar

Table S1: Inclusion and exclusion criteria

Reference	Exclusion criteria										Inclusion		
	Computational study		Non-coronavirus study		Non-helicase study		Review article		Irrelevant study				
	Coder 1	Coder 1	Coder 1	Coder 2	Coder 1	Coder 2	Coder 1	Coder 2	Coder 1	Coder 2	Coder 1	Coder 2	After revision
[25]									✓	✓	**No**	**No**	
[26]		✓									Yes	**No**	Yes
[27]											Yes	Yes	
[31]	✓										**No**	Yes	**No**
[32]							✓				**No**	Yes	**No**
[33]	✓										**No**	Yes	Yes
[34]	✓										**No**	Yes	**No**
[35]							✓	✓			**No**	**No**	
[36]							✓	✓			**No**	**No**	
[37]	✓	✓									**No**	**No**	
[38]	✓	✓									**No**	**No**	
[39]	✓	✓									**No**	**No**	
[40]	✓	✓									**No**	**No**	
[41]	✓	✓									**No**	**No**	
[42]	✓	✓									**No**	**No**	
[43]	✓	✓									**No**	**No**	
[44]					✓	✓					**No**	**No**	
[45]					✓	✓					**No**	**No**	
[46]					✓	✓					**No**	**No**	
[47]			✓	✓							**No**	**No**	
[48]			✓	✓							**No**	**No**	
[49]			✓	✓							**No**	**No**	
[50]									✓	✓	**No**	**No**	
[51]									✓	✓	**No**	**No**	
[52]									✓	✓	**No**	**No**	

[53]											Yes	Yes	Excluded after full text revision
[54]											Yes	Yes	Excluded after full text revision
[55]											Yes	Yes	
[56]											Yes	Yes	
[57]											Yes	Yes	
[58]											Yes	Yes	
[59]											Yes	Yes	
[60]											Yes	Yes	
[61]											Yes	Yes	
[62]											Yes	Yes	
[63]											Yes	Yes	
[64]											Yes	Yes	
[65]											Yes	Yes	
[66]											Yes	Yes	
[67]											Yes	Yes	
[68]											Yes	Yes	
[69]											Yes	Yes	
[70]											Yes	Yes	
[71]											Yes	Yes	
[72]											Yes	Yes	
[73]											Yes	Yes	
[74]											Yes	Yes	
[75]											Yes	Yes	
[76]											Yes	Yes	
[77]											Yes	Yes	
[78]											Yes	Yes	
[79]											Yes	Yes	
[80]											Yes	Yes	
[81]											Yes	Yes	
[82]											Yes	Yes	

Table S2: Kinetic properties of the helicases used in inhibition studies

Reference	Virus	Substrate/cofactor	Activity	[Nsp13]	K_m	V_{max}
[26]	SARS-CoV-2	dsDNA	Unwinding	50 nM	59 ± 10 nM	6.3 ± 3.0 nM min^{-1}
		ATP	Unwinding		2.0 ± 0.13 mM	3.3 ± 0.10 nM min^{-1}
[27]	MERS-CoV	dsDNA	Unwinding	50 nM	69.86 ± 11.02 nM	35.86 ± 2.607 nM min^{-1}
		dsRNA	Unwinding		59.64 ± 5.940 nM	53.97 ± 2.358 nM min^{-1}
		ATP (dsDNA sec. sub.)	Unwinding		1.425 ± 0.3599 mM	2.381 ± 0.2443 nM min^{-1}
		ATP (dsRNA sec. sub.)	Unwinding		1.649 ± 0.4700 mM	2.839 ± 0.3599 nM min^{-1}
[56]	SARS-CoV	Oligo. dT_{24}	ATPase	0.064 µg/µL	(~ 50-100 nM)	(~ 0.4 - 0.6 a.u.)
		ATP	ATPase		(~ 0.1-1 mM) Figure (b,c)	(~ 1.0 - 5.0 a.u.) Figure (b,c)
[64]	SARS-CoV	dsDNA	Unwinding	50 nM	(~ 90 nM)	(~ 10000 a.u. min^{-1})
		ATP	Unwinding		(~ 0.2 mM) Figure 6 (a,b)	(~ 40000 a.u. min^{-1}) Figure 6 (a,b)
[72]	SARS-CoV-2	dsDNA 5’ tailed	Unwinding	10 pmol	Active	
		dsDNA 3’ tailed			Non-active	
		dsDNA blunt			Non-active	
		Mg^{2+}, Mn^{2+}, Zn^{2+}, Ca^{2+}	Unwinding		Activity at 2mM: $Mg^{2+} > Mn^{2+}$ Zn^{2+}, Ca^{2+} (non-active) Mg^{2+} inhibitory at > 2mM	
		ATP, GTP, UTP, CTP	ATPase		Activity: ATP > GTP > UTP > CTP	
		Mg^{2+}, Mn^{2+}, Zn^{2+}, Ca^{2+}	ATPase		Activity at 2mM: $Mg^{2+} > Mn^{2+} > Zn^{2+} > Ca^{2+}$ Mg^{2+} inhibitory at > 2mM	
[73]	SARS-CoV-2	dsDNA	Unwinding	10 nM	42.21 nM	20.53 ± 1.56 $nMmin^{-1}$
		ATP	ATPase	2 nM	5.51 mM	42.05 ± 2.78 $mMmin^{-1}$
[74]	SARS-CoV-2	dsDNA	Unwinding	1 nM	2.6 µM	(> 1500 a.u. min^{-1})
		dsRNA	Unwinding		1.0 µM	(> 300 a.u. min^{-1})
		ATP (dsDNA sec. sub.)	Unwinding		0.11 mM	(~ 1000 a.u. min^{-1})
		ATP (dsRNA sec. sub.)	Unwinding		0.13 mM	(~ 1200 a.u. min^{-1}) Figure 2 (a,b,c,d)
[75]	SARS-CoV-2	Oligo poly A (activator)	ATPase	1 nM	0.022 ± 0.004 mM	-
		dsDNA	Unwinding		1.22 ± 0.29 µM	(> 1.2 pM min^{-1})
		ATP	Unwinding		0.47 ± 0.06 mM	(~ 1.2 pM min^{-1})
		ATP	ATPase		0.043 ± 0.012 mM	(~ 0.12 nM min^{-1}) Figure 1 (b,c,d)
		dsDNA (+ BSA/TCEP)	Unwinding		1.68 ± 0.60 µM	(> 1.5 pM min^{-1})

		ATP (+ BSA/TCEP)	Unwinding		0.122 ± 0.016 mM	(~ 0.8 pM min^{-1})
		ATP (+ BSA/TCEP)	ATPase		(~ 0.025 mM)	(~ 0.065 nM min^{-1})
[80]	SARS-CoV-2	ATP (no ssDNA)	ATPase	500 pM	210 ± 20 nM	0.42 nM min^{-1}
		ATP	ATPase		2800 ± 1000 nM	5.6 nM min^{-1}
		ssDNA			3.3 ± 0.2	0.007 nM min^{-1}
[81]	SARS-CoV-2	ATP	Unwinding	50 nM	FRET increases with ATP in dose dependent manner	-

Values between parenthesis: extrapolated from the mentioned figure in the original publication

Table S3: Inhibition kinetic parameters of certain inhibitors

**Reference**	**Virus**	**Inhibitor**	**Substrate**	**Activity**	K_m^{App}	V_{max}^{App}	K_I	**Type of Inhibition**
[56]	SARS-CoV	Bananin	Oligo. dT_{24}	ATPase	(~ 0.25-0.4 mM)	(~ 0.5-1.0 a.u. min^{-1})	*~ 0.6-2.3 μM*	Non-competitive
		Bananin	ATP	ATPase	(~ 40-110 mM)	(~ 0.2-0.3 a.u. min^{-1})	*~ 2.3 μM*	Non-competitive
					Figure 3 (a,b,c,d)	Figure 3 (a,b,c,d)		
[64]	SARS-CoV	SSYA10-001	dsDNA	Unwinding	(~ 0.2 mM)	(~ 20000 a.u. min^{-1})	*~ 20 μM*	Non-competitive
		SSYA10-001	ATP	Unwinding	(~ 90 mM)	(~ 4000 a.u. min^{-1})	*~ 20 μM*	Non-competitive
					Figure 6 (a,b)	Figure 6 (a,b)		
[73]	SARS-CoV-2	RBC	dsDNA	Unwinding	91.77 nM	-	0.39 ± 0.07 μM	Competitive
		RBC	ATP	ATPase	12.74 mM	-	0.97 ± 0.11 μM	Competitive
[78]	SARS-CoV-2	Licoflavone C	ATP	Unwinding	(~ 0.4 mM)	(~ 0.05 pM min^{-1})	*~ 2 μM*	Non-competitive
		Licoflavone C	ATP	ATPase	(~ 0.08 mM)	(~ 0.0286 pM min^{-1})	~ 0.2 μM	Non-competitive
		Kampferol	ATP	Unwinding	(~ 0.8 mM)	(~ 1.0 pM min^{-1})	*~ 0.8 μM*	Non-competitive
		Flavanone	ATP	Unwinding	(~ 0.8 mM)	(~ 0.667 pM min^{-1})	*~ 1.4 μM*	Non-competitive
					Figure 6 (a,b,c,d)	Figure 6 (a,b,c,d)		
[81]	SARS-CoV-2	Punicalagin	ATP	ATPase	-	-	Figure 5b	Competitive

Values between parenthesis: extrapolated from the figure in publication

Italic: calculated K_I

K_I for non-competitive calculated by the equation [I]/(α'-1), where $\alpha' = V_{max} / V_{max}^{App}$ and [I] is the highest inhibitor concentration. It is assumed that inhibitor binds to E and ES complex with same affinity (α'= α, and $K_I = K'_I$).

K_I for competitive calculated by the equation [I]/(α-1), where $\alpha = K_m / K_m^{App}$ and [I] is the highest inhibitor concentration. It is assumed that $V_{max} = V_{max}^{App}$ and $K_m^{App} > K_m$ (α> 1).

Actual Vmax values are taken from table (S3).

Table S4: Purification and enzymatic assay conditions

Study	Virus	Assay	Buffer	Volume	Nsp13	Substrate 1	Substrate 2	Screening Library	Plasmid
[22]	SARS-CoV-2	Phosphate release	[23]						pET-52b
		FRET-based							
[27]	MERS-CoV	Phosphate release	20 mM HEPES, pH 7.5, 20 mM potassium glutamate, 1 mM DTT, 0.005% Triton X-100, 10 mM MgCl2	30 µl	20 nM	0.25 mM ATP	250 nM dsDNA	-	pET-52b
		FRET-based	20 mM HEPES, 20 mM NaCl, 5 mM MgCl2, 0.01% BSA, 2 mM DTT, 5% glycerol, 2 µM trap, unlabelled single-stranded	100 µl	50 nM	0.5 mM ATP	75 nM fluorescein- and black hole quencher-labelled dsDNA	Tocriscreen Library of Marketed FDA-Approved Compounds)	
[33]	SARS-CoV-2	Phosphate release	25 mM HEPES (pH 7.5), 50 mM NaCl, 5 mM MgCl2, and 1 mM DTT	20 µL	150 nM	0.25 mM ATP	-	Compounds suggested by virtual screening	pET28a
[55]	SARS-CoV	Phosphate release	50 mM Tris-HCl, pH 6.6, and 5 mM MgCl2	50 µL	0.2–0.7 pmol	1mM ATP	25µ g/ml poly(U)	Compounds suggested by screening ChemBridge Corporation library against viral activity	pET32a
		FRET-based (SDS-PAGE)	20 mM Hepes, pH 7.4, 5 mM magnesium acetate, 2 mM DTT, 0.1 mg/ml bovine serum albumin, 10% glycerol)	60 µL	6 pmol	2.5 mM ATP	30 fM [γ-32P] dsDNA		
[56]	SARS-CoV	Phosphate release	50 mM Tris-HCl, pH 6.8, and 5 mM MgCl2, and 0.1 mg/mL BSA	-	3.2 ng	2 mM ATP	200 nM dT_{24}	In house synthesized bananin derivatives	pET28a
		FRET-based	50 mM Tris-HCl, pH 6.8, and 5 mM MgCl2, and 0.1 mg/mL BSA	-	2 nM	0.5 mM ATP	5 nM dsDNA		
[57]	SARS-CoV	Phosphate release	50 mM Tris-HCl, pH 6.8, and 5 mM MgCl2	50µL	0.7 pmol	1 mM ATP	25 µg/ml poly(U)	In house synthesized bismuth complexes	pET28a
		FRET-based (SDS-PAGE)	20 mM Hepes, pH 7.4, 5 mM magnesium acetate, 2 mM TT, 0.1 mg/ml BSA, and 10% glycerol	-	0.3 pmol	2.5 mM ATP	600 fM [γ-32P] dsDNA		
[58]	SARS-CoV	Phosphate release	[53]						
		FRET-based							
[59]	SARS-CoV	Phosphate release	50 mM Tris-HCl buffer (pH 6.8), 50 mM NaCl, and 5 mM MgCl2	50µL	0.1 µM	0.5 mM ATP	100 nM Poly (rU)	In house synthesized aptamers	pHelA12
		FRET-based	20 mM HEPES (pH 7.4), 0.1 M NaCl, and 2.5 mM MgCl2,	50µL	20 nM	2 mM ATP	100 nM dsDNA		
[60]	SARS-CoV	Phosphate release	50 mM Tris-HCl, pH 6.6, and 5 mM MgCl2	50µL	0.7 pmol	1mM ATP	25 µg/ml poly(U)	In house synthesized aptamers	pHelA12

		FRET-based (SDS-PAGE)	20 mM Hepes, pH 7.4, 5 mM magnesium acetate, 2 mM DTT, 0.1 mg/ml BSA, and 10% glycerol	100µL	6 pmol	2.5 mM ATP	30 fM [γ-32P] dsDNA		
[61]	SARS-CoV	Phosphate release	[59]					In house synthesized aryl diketoacid derivatives	pET28a
		FRET-based							
[62]	SARS-CoV	Phosphate release	[59]					In house synthesized dihydroxychromone derivatives	pET28a
		FRET-based							
[63]	SARS-CoV	Phosphate release	50 mM Tris/HCl (pH 6.6), 100 mM NaCl, 10 mM MgCl2	100 µl	400 nM	4 mM ATP	4 nM circular ssDNA	In house synthesized 2,6-Bis-arylmethyloxy-5-hydroxychromone derivatives	pET28a
		FRET-based	20 mM HEPES (pH 7.4), 1 mM MgCl2, and 5 mM DTT	100 µl	150 nM	9 mM ATP	20 nM dsDNA		
[64]	SARS-CoV	Phosphate release (SDS-PAGE)	20 mM HEPES, 20 mM NaCl, 0.01% BSA, 2 mM DTT, 5% glycerol, 5 mM MgCl2	-	50 nM	5 µM [γ-^{32}P] ATP	100 nM dsDNA	Maybridge HitFinder	pET28a
		FRET-based (SDS-PAGE)	20 mM HEPES, 20 mM NaCl, 0.01% BSA, 2 mM DTT, 5% glycerol, 5 mM MgCl2	-	50 nM	2 mM ATP	100 nM dsDNA		
		FRET-based	20 mM HEPES, 20 mM NaCl, 0.01% BSA, 2 mM DTT, 5% glycerol, 5 mM MgCl2	50 µl	50 nM	0.5 mM ATP	2 µM dsDNA		
[65]	MER-CoV	Phosphate release	[60]						
		FRET-based							
[66]	SARS-CoV	Phosphate release	[59]					Natural compounds	pET28a
		FRET-based							
[67]	SARS-CoV	Phosphate release	[59]					Natural product	pET28a
		FRET-based							
[68]	SARS-CoV	Phosphate release	[65]						
		FRET-based							
[69]	SARS-CoV	Phosphate release	[65]						
		FRET-based							
[70]	SARS-CoV	Phosphate release	50 mM Tris-HCl (pH 6.6), 100 mM NaCl, and 10 mM MgCl2	50 µl	400 nM	4 mM ATP	4 nM circular ssDNA	In house compounds	pET28a
		FRET-based	20 mM HEPES (pH 7.4), 1 mM MgCl2, and 5 mM DTT	100 µl	150 nM	9 mM ATP	20 nM		
[71]	MER-CoV	Phosphate release	50 mM Tris-HCl (pH 6.8), 5 mM MgCl2, 0.1 mg/mL BSA	50 µL.	3.2 ng	1 mM ATP	20 nM	In house compounds	pET28a
		FRET-based	30 mM Tris–HCl (pH 7.5), 5 mM MgCl2, 0.075% Triton X-100, 0.05% sodium azide, and 250 nM capture strand	200 µl	2–20 nM	1 mM ATP	20 nM		

[72]	SARS-CoV-2	Phosphate release	25 mM HEPES-KOH (pH 8.0), 50 mM NaCl, 1 mM MgCl2, and 1 mM DTT	20 µL	10 pmol	1 mM ATP	-	Commercial compounds	pMAL-c2X
		FRET-based	50 mM HEPES–KOH (pH 8.0), 2 mM $MgCl_2$, 100 mM $NaCl_2$ and 20 U RNasin	-	20 pmol	5 mmol/L ATP	0.1 pM dsDNA		
[73]	SARS-CoV-2	Phosphate release	20mM Tris-HCl, pH6.8, 5mM MgCl2, and 2mM TCEP	50µl	2nM	0.5 mM ATP	0.04 $mgml^{-1}$ poly(U)	Commercial compounds	pET28
		FRET-based	20mM Tris-HCl buffer, pH7.4, 150mM NaCl, 0.1$mgml^{-1}$ BSA, 5mM MgCl2, 5mM TCEP, and 5% glycerol	50µl	10nM	1 mM ATP	5nM dsDNA		
[74]	MER-CoV SARS-CoV-2	FRET-based	20 mM Tris-HCl buffer, pH 7.4, 150 mM NaCl, 0.1 mg/ml BSA, 5 mM MgCl2, 5 mM TCEP, and 5% glycerol	100 µL	10 nM	0.5 mM ATP	5 nM dsDNA	Clofazimine	pET28
[75]	SARS-CoV-2	Phosphate release	33 mM Tris-HCl (pH 8.0) and 5 mM MgCl2	100 µL	2.5 µM	5 mM ATP	0.25 µM dT_{24}	Disulfiram and Ebselen	pET28
[76]	SARS-CoV-2	FRET-based	50 mM Tris-HCl, pH 7.5, and 0.075% Triton X-100	100 µL	80 nM	1 mM ATP	100 nM dsDNA	Natural product	pTrcHisB
[77]	SARS-CoV-2	FRET-based	20 mM HEPES pH 7.6, 20 mM NaCl, 5 mM MgCl2, 1 mM DTT and 0.1 mg/ml BSA	20 µL	6 nM	200 µM ATP	360 nM dsDNA	NA	biGBac vector pLIB
[78]	SARS-CoV-2	Phosphate release	20 mM Tris–HCl, pH 7.2, 50 mM NaCl, 2 mM and MgCl2	25 µL	25 nM	400 µM ATP	-	In house library of flavonoids	pNIC-ZB
		FRET-based	20 mM Tris–HCl, pH 7.2, 50 mM NaCl, 2 µM Hel Capture oligo, and 5 mM MgCl2	40 µL	1 nM	1 mM ATP	750 nM dsDNA		
[79]	SARS-CoV-2	ATP luciferase	50 mM HEPES, pH 7.5, 5% glycerol, 5 mM magnesium acetate, 5 mM DTT, and 0.01% BSA	14 µL	0.1 nM	2.5 µM ATP	3.5 nM ssDNA	ChemDiv Kinase Library	pFBOH-MHL
[80]	SARS-CoV-2	ATP luciferase	50 mM HEPES pH 7.2, 150 mM NaCl, 10 mM MgCl2 and 0.5% Tween 20	20 µL	50 nM	10 µM ATP	-	FDA-approved drugs or bioactive compounds (TopSicence)	pET-28a
		FRET-based	50 mM HEPES, pH 7.2, 20 mM NaCl, 625 nM single strand trap, 0.1mg/mL BSA, and 4 mM MgCl2	20 µL	50 nM	2 mM ATP	250 nM dsDNA		
[81]	SARS-CoV-2	Phosphate release	20 mM Tris–HCl, pH 7.2, 50 mM NaCl, 2 mM MgCl2, 5% DMSO	40 µL	25 nM	400 µM ATP	-	In house compounds	Enzyme donated
		FRET-based	0 mM Tris–HCl, pH 7.2, 50 mM NaCl, 2 µM Hel Capture	40 µL	1 nM	1 mM ATP	750 nM dsDNA		

			oligo, 5 mM MgCl2, 5% DMSO						
[82]	SARS-CoV-2	Phosphate release	20 mM Tris–HCl pH 7.2, 50 mM NaCl, 2 mM MgCl2, 10 µg/mL of BSA, 180 µM TCEP, 5% DMSO	25 µL	25 nM	400 µM ATP	-	In house compounds	pNIC-ZB
		FRET-based	20 mM Tris–HCl pH 7.2. 50 mM NaCl, 2 mM MgCl2, 10 µg/mL of BSA, 180 µM TCEP, 2 µM Hel Capture oligo, 5% DMSO	25 µL	1 nM	1 mM ATP	750 nM dsDNA		

```
MERS-CoV      AVGSCVVCHSQTSLRCGTCIRRPFLCCKCCYDHVIATPHKMVLSVSPYVCNAPGCGVSDV   60
SARS-CoV      AVGACVLCNSQTSLRCGACIRRPFLCCKCCYDHVISTSHKLVLSVNPYVCNAPGCDVTDV   60
SARS-CoV-2    AVGACVLCNSQTSLRCGACIRRPFLCCKCCYDHVISTSHKLVLSVNPYVCNAPGCDVTDV   60
                                    ZBD
MERS-CoV      TKLYLGGMSYFCVDHRPVCSFPLCANGLVFGLYKNMCTGSPSIVEFNRLATCDWTESGDY   120
SARS-CoV      TQLYLGGMSYYCKSHKPPISFPLCANGQVFGLYKNTCVGSDNVTDFNAIATCDWTNAGDY   120
SARS-CoV-2    TQLYLGGMSYYCKSHKPPISFPLCANGQVFGLYKNTCVGSDNVTDFNAIATCDWTNAGDY   120
                                                       Stalk
MERS-CoV      TLANTTTEPLKLFAAETLRATEEASKQSYAIATIKEIVGERQLLLVWEAGKSKPPLNRNY   180
SARS-CoV      ILANTCTERLKLFAAETLKATEETFKLSYGIATVREVLSDRELHLSWEVGKPRPPLNRNY   180
SARS-CoV-2    ILANTCTERLKLFAAETLKATEETFKLSYGIATVREVLSDRELHLSWEVGKPRPPLNRNY   180
                                                        1B
MERS-CoV      VFTGYHITKNSKVQLGEYIFERIDYSDAVSYKSSTTYKLTVGDIFVLTSHSVATLTAPTI   240
SARS-CoV      VFTGYRVTKNSKVQIGEYTFEKGDYGDAVVYRGTTTYKLNVGDYFVLTSHTVMPLSAPTL   240
SARS-CoV-2    VFTGYRVTKNSKVQIGEYTFEKGDYGDAVVYRGTTTYKLNVGDYFVLTSHTVMPLSAPTL   240

MERS-CoV      VNQERYVKITGLYPTITVPEEFASHVANFQKSGYSKYVTVQGPPGTGKSHFAIGLAIYYP   300
SARS-CoV      VPQEHYVRITGLYPTLNISDEFSSNVANYQKVGMQKYSTLQGPPGTGKSHFAIGLALYYP   300
SARS-CoV-2    VPQEHYVRITGLYPTLNISDEFSSNVANYQKVGMQKYSTLQGPPGTGKSHFAIGLALYYP   300
                                                  Rec1A
MERS-CoV      TARVVYTACSHAAVDALCEKAFKYLNIAKCSRIIPAKARVECYDRFKVNETNSQYLFSTI   360
SARS-CoV      SARIVYTACSHAAVDALCEKALKYLPIDKCSRIIPARARVECFDKFKVNSTLEQYVFCTV   360
SARS-CoV-2    SARIVYTACSHAAVDALCEKALKYLPIDKCSRIIPARARVECFDKFKVNSTLEQYVFCTV   360

MERS-CoV      NALPETSADILVVDEVSMCTNYDLSIINARIKAKHIVYVGDPAQLPAPRTLLTRGTLEPE   420
SARS-CoV      NALPETTADIVVFDEISMATNYDLSVVNARLRAKHYVYIGDPAQLPAPRTLLTKGTLEPE   420
SARS-CoV-2    NALPETTADIVVFDEISMATNYDLSVVNARLRAKHYVYIGDPAQLPAPRTLLTKGTLEPE   420

MERS-CoV      NFNSVTRLMCNLGPDIFLSMCYRCPKEIVSTVSALVYNNKLLAKKELSGQCFKILYKGNV   480
SARS-CoV      YFNSVCRLMKTIGPDMFLGTCRRCPAEIVDTVSALVYDNKLKAHKDKSAQCFKMFYKGVI   480
SARS-CoV-2    YFNSVCRLMKTIGPDMFLGTCRRCPAEIVDTVSALVYDNKLKAHKDKSAQCFKMFYKGVI   480
                                                  Rec2A
MERS-CoV      THDASSAINRPQLTFVKNFITANPAWSKAVFISPYNSQNAVSRSMLGLTTQTVDSSQGSE   540
SARS-CoV      THDVSSAINRPQIGVVREFLTRNPAWRKAVFISPYNSQNAVASKILGLPTQTVDSSQGSE   540
SARS-CoV-2    THDVSSAINRPQIGVVREFLTRNPAWRKAVFISPYNSQNAVASKILGLPTQTVDSSQGSE   540

MERS-CoV      YQYVIFCQTADTAHANNINRFNVAITRAQKGILCVMTSQALFESLEFTELSFTNYKLQ--   598
SARS-CoV      YDYVIFTQTTETAHSCNVNRFNVAITRAKIGILCIMSDRDLYDKLQFTSLEIPRRNVATL   600
SARS-CoV-2    YDYVIFTQTTETAHSCNVNRFNVAITRAKVGILCIMSDRDLYDKLQFTSLEIPRRNVATL   600

MERS-CoV      -   598
SARS-CoV      Q   601
SARS-CoV-2    Q   601
```

Figure S1: Multiple sequence alignment of **YP_009725308.1**, (SARS-CoV-2 nsp13), **NP_828870.1** (SARS-CoV nsp13), and **YP_009047224.1** (MERS-CoV nsp13) was generated with the Clustal Omega program (McWilliam et al., 2013). The domains of SARS-CoV-2 were defined and underlined as follows: ZBD (red), stalk (yellow), 1B, Rec1A (magenta), and Rec2A (blue) (Domains and McCullagh, 2021). The nucleotide binding site residues (blue) and nucleic acid binding site (magenta) were mapped as previously established (Newman et al., 2021). The putative binding site in MERS-CoV is also mapped as previously published (Adedeji et al., 2014).

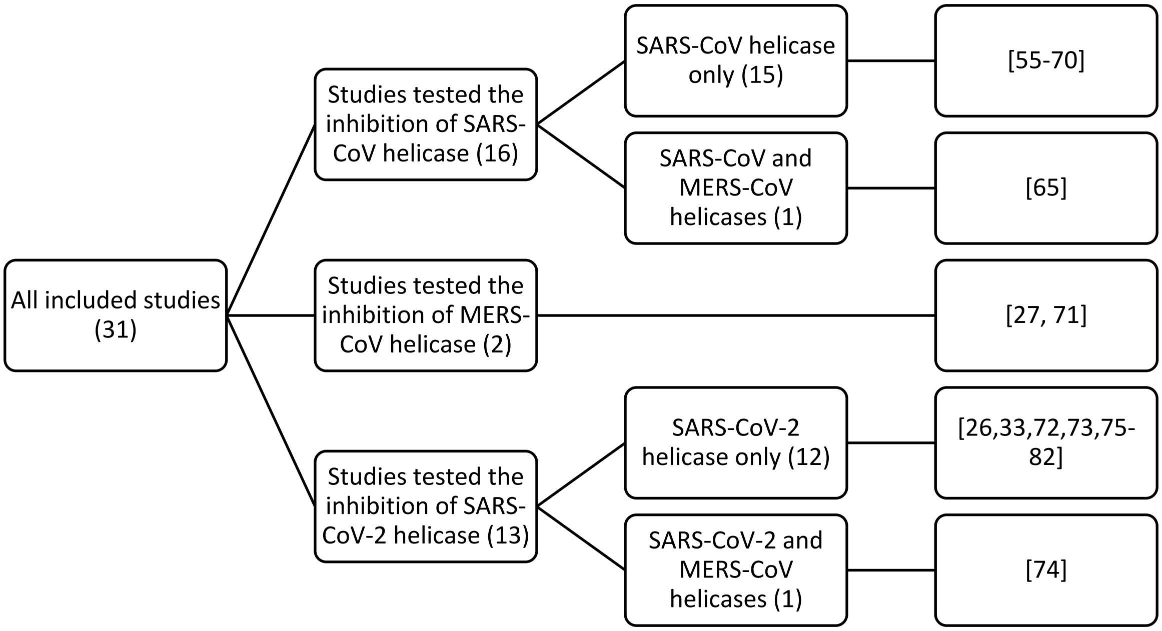
All included studies (31)
Studies tested the inhibition of SARS-CoV helicase (16)
Studies tested the inhibition of MERS-CoV helicase (2)
Studies tested the inhibition of SARS-CoV-2 helicase (13)
SARS-CoV helicase only (15)
SARS-CoV and MERS-CoV helicases (1)
SARS-CoV-2 helicase only (12)
SARS-CoV-2 and MERS-CoV helicases (1)
[55-70]
[65]
[27, 71]
[26,33,72,73,75-82]
[74]

A

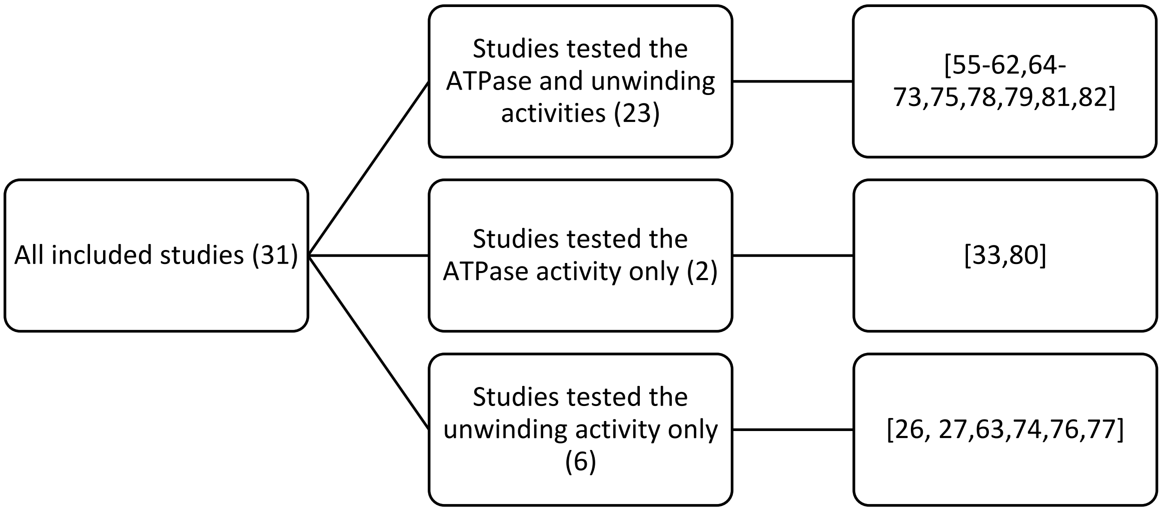
All included studies (31)
Studies tested the ATPase and unwinding activities (23)
Studies tested the ATPase activity only (2)
Studies tested the unwinding activity only (6)
[55-62,64-73,75,78,79,81,82]
[33,80]
[26, 27,63,74,76,77]

B

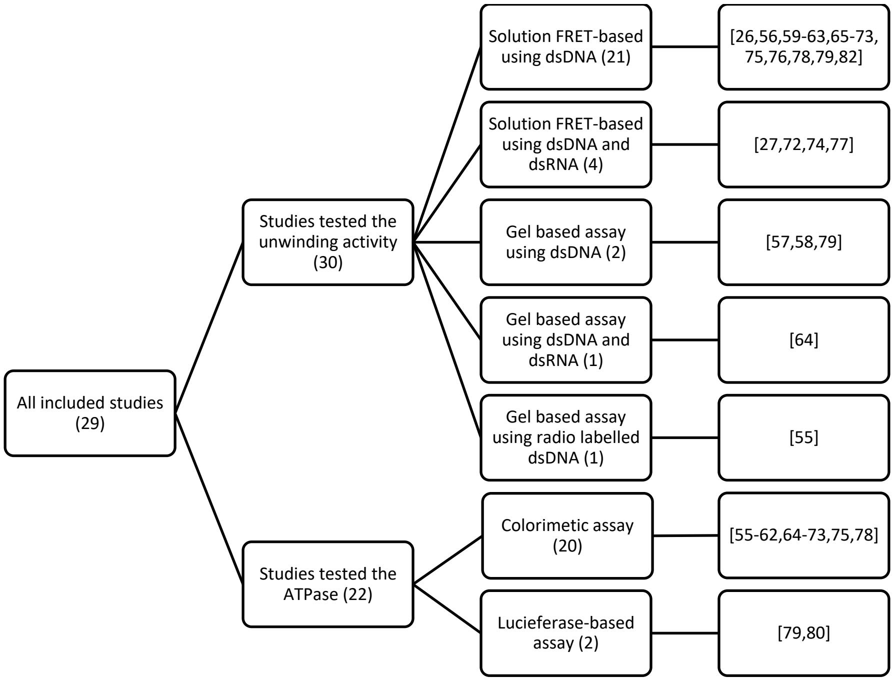
All included studies (29)
Studies tested the unwinding activity (30)
Studies tested the ATPase (22)
Solution FRET-based using dsDNA (21)
Solution FRET-based using dsDNA and dsRNA (4)
Gel based assay using dsDNA (2)
Gel based assay using dsDNA and dsRNA (1)
Gel based assay using radio labelled dsDNA (1)
Colorimetic assay (20)
Lucieferase-based assay (2)
[26,56,59-63,65-73, 75,76,78,79,82]
[27,72,74,77]
[57,58,79]
[64]
[55]
[55-62,64-73,75,78]
[79,80]

C

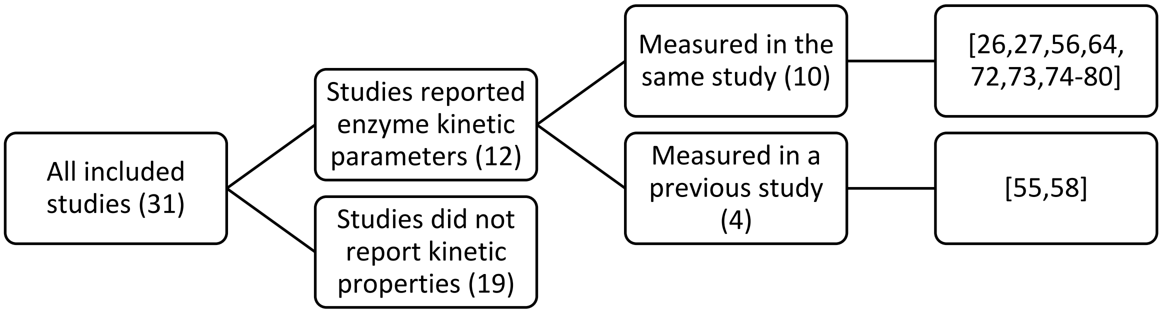
All included studies (31)
Studies reported enzyme kinetic parameters (12)
Studies did not report kinetic properties (19)
Measured in the same study (10)
Measured in a previous study (4)
[26,27,56,64, 72,73,74-80]
[55,58]

D

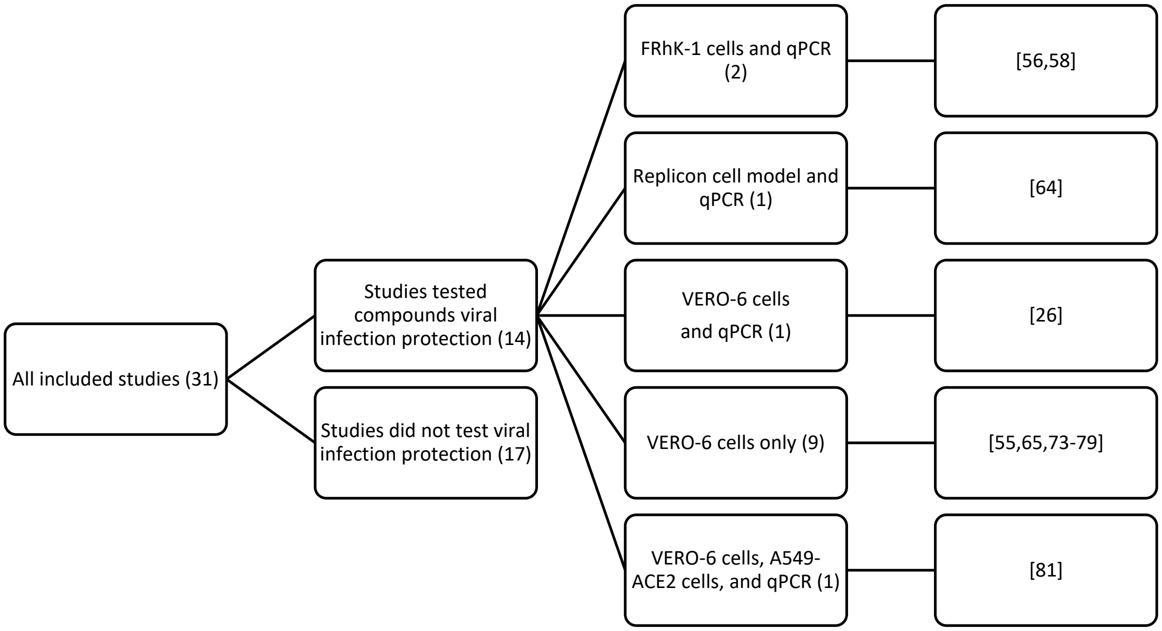

E

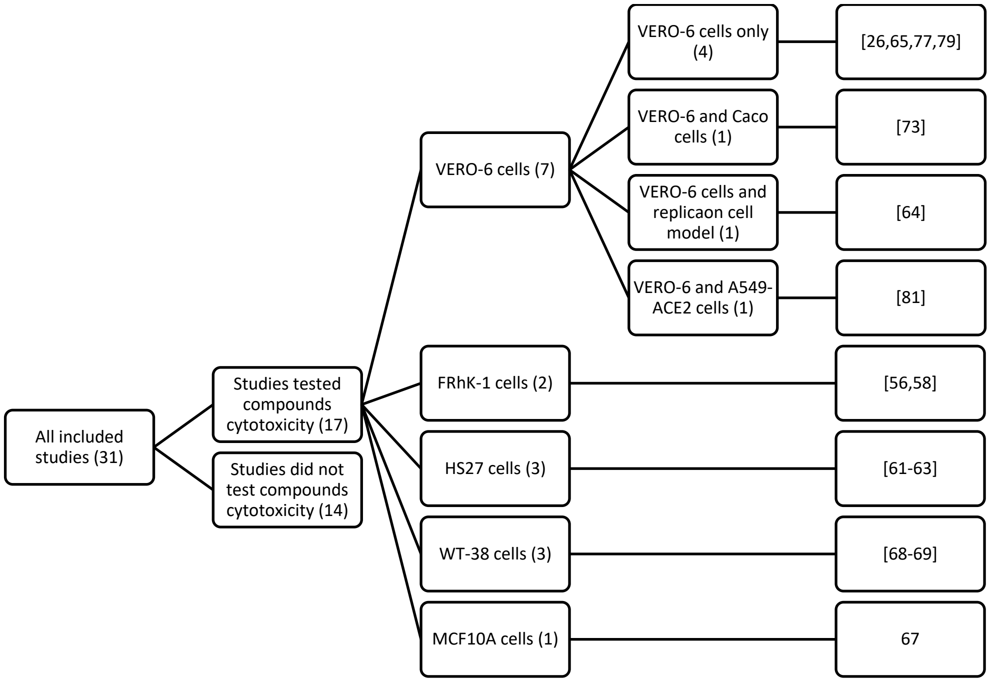

F

Figure S2: Study characteristics based on tested virus (A), tested enzymatic activity (B), type of assay (C), reported kinetic parameters (D), measured viral infection protection properties (E), and cytotoxicity (F)

References:

Adedeji, A. O., Singh, K., Kassim, A., Coleman, C. M., Elliott, R., Weiss, S. R., Frieman, M. B., Sarafianos, S. G. 2014. Evaluation of SSYA10-001 as a replication inhibitor of severe acute respiratory syndrome, mouse hepatitis, and Middle East respiratory syndrome coronaviruses. Antimicrobial agents and chemotherapy, 58, 4894–4898. https://doi.org/10.1128/AAC.02994-14

Domains Weber, R., McCullagh, M. 2021. Role of ATP in the RNA Translocation Mechanism of SARS-CoV-2 NSP13 Helicase. The journal of physical chemistry. B, 125, 8787–8796. https://doi.org/10.1021/acs.jpcb.1c04528

McWilliam, H., Li, W., Uludag, M., Squizzato, S., Park, Y. M., Buso, N., Cowley, A. P., Lopez, R. 2013. Analysis Tool Web Services from the EMBL-EBI. Nucleic acids research, 41(Web Server issue), W597–W600. https://doi.org/10.1093/nar/gkt376

Newman, J. A., Douangamath, A., Yadzani, S., Yosaatmadja, Y., Aimon, A., Brandão-Neto, J., Dunnett, L., Gorrie-Stone, T., Skyner, R., Fearon, D., Schapira, M., von Delft, F., Gileadi, O. 2021. Structure, mechanism and crystallographic fragment screening of the SARS-CoV-2 NSP13 helicase. Nature communications, 12, 4848. https://doi.org/10.1038/s41467-021-25166-6
