# Supplementary material for: Coronaviruses SARS-CoV, MERS-CoV, and SARS-CoV-2 helicase inhibitors: a systematic review of invitro studies
Source: J Virus Erad. 2023 May 26;9(2):100327. doi: 10.1016/j.jve.2023.100327 (PMC10214743; doi:10.1016/j.jve.2023.100327)
Supplement: Multimedia component 2 [file mmc2.pdf]

Table S5: Names, CAS number and chemical structures of tested compounds

Publication	Compound		
Adedeji et al. (2012a) [64]<br><br>Adedeji et al. (2014) [65]	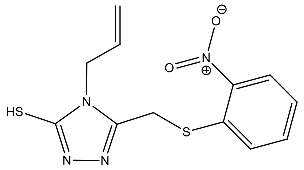<br>SSYA10-001<br>675104-49-1	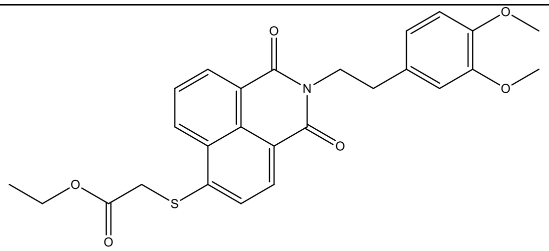<br>SSYA10-002<br>280140-63-8	
Chen et al. (2021) [75]	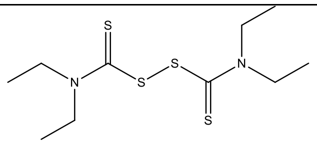<br>Disulfiram<br>97-77-8	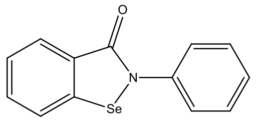<br>Ebselen<br>60940-34-3	
Cho et al. (2015) [68]	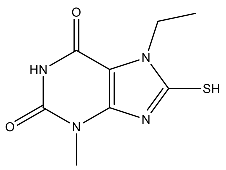<br>7-ethyl-8-mercapto-3-methyl-3,7-dihydro-1H-purine-2,6-dione<br>(EMMDPD)<br>126118-56-7		
Corona et al. (2022) [78]	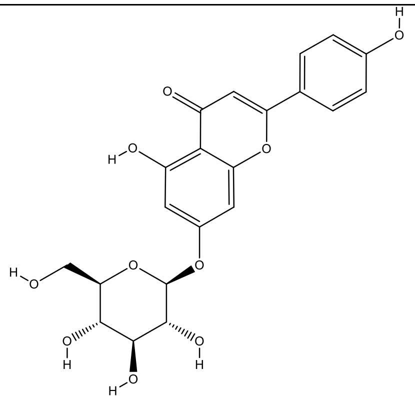<br>Apigenin-7-O-glucoside<br>578-74-5	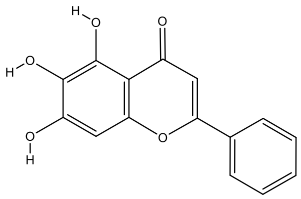<br>Baicalein<br>491-67-8	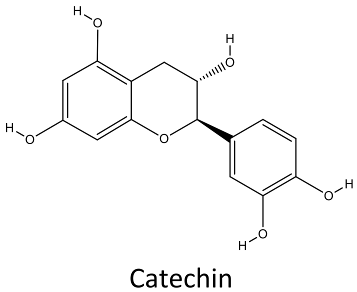<br>Catechin<br>154-23-4
	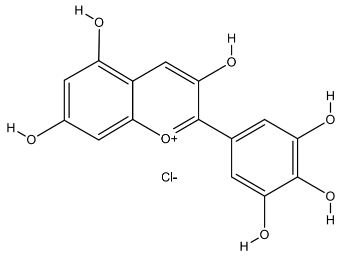<br>Dihydromyricetin	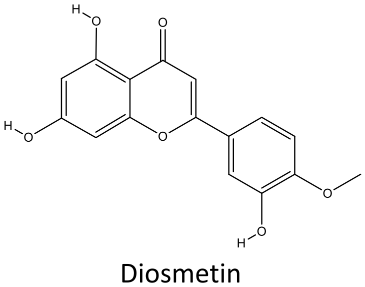<br>Diosmetin	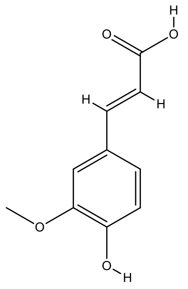

27200-12-0

520-34-3

Ferulic acid
1135-24-6

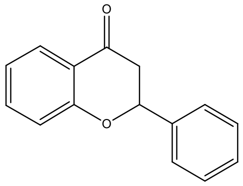

Flavanone
487-26-3

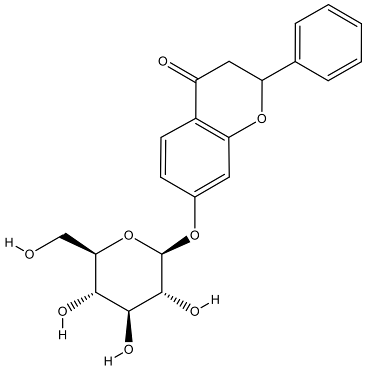

Flavanone-7-O-glucoside
(No CAS)

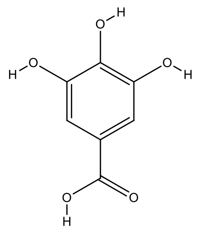

Gallic acid
149-91-7

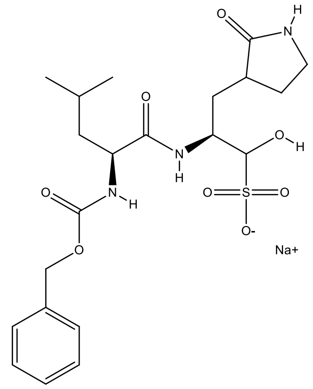

GC376
1416992-39-6

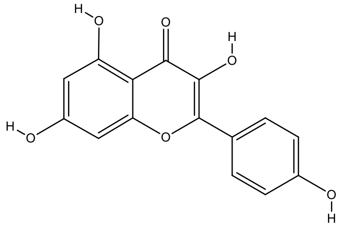

Kaempferol
520-18-3

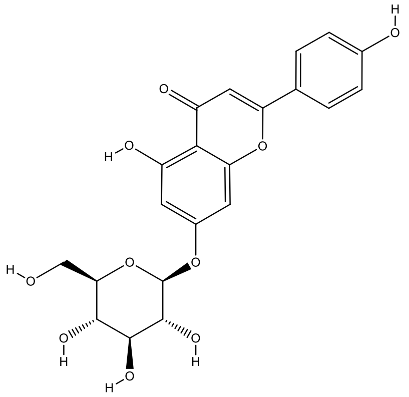

Kaempferol-3-O-rutinoside
17650-84-9

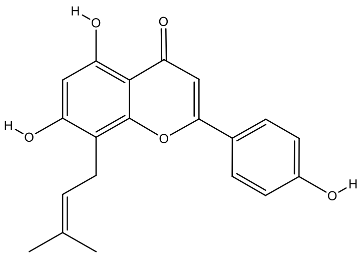

Licoflavone C
72357-31-4

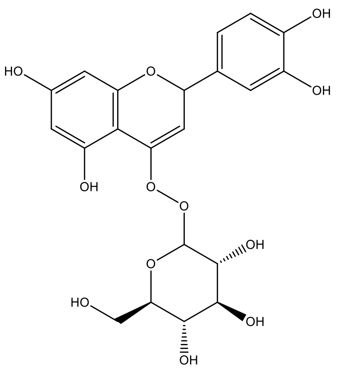

Luteoline-4’-O-glucoside
6920-38-3

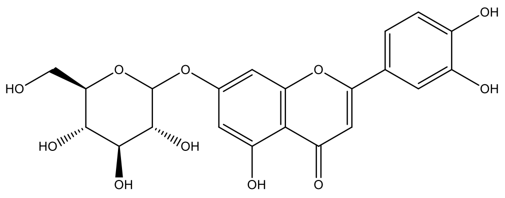

Luteoline-7-O-glucoside
5373-11-5

Myricetin
See Zeng et al. (2021)

	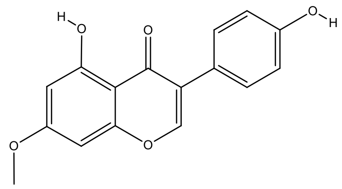<br>Prunetin<br>552-59-0<br>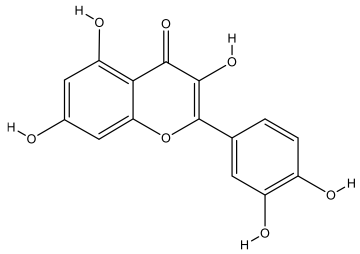<br>Quercetin<br>117-39-5<br>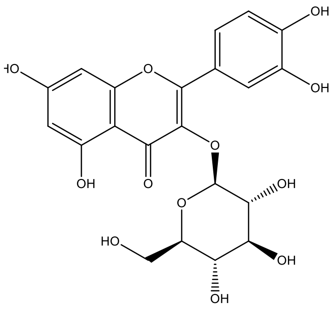<br>Quercetin-3-O-β-glucoside<br>482-35-9<br>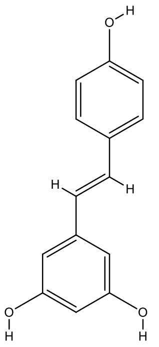<br>Resveratrol<br>501-36-0<br>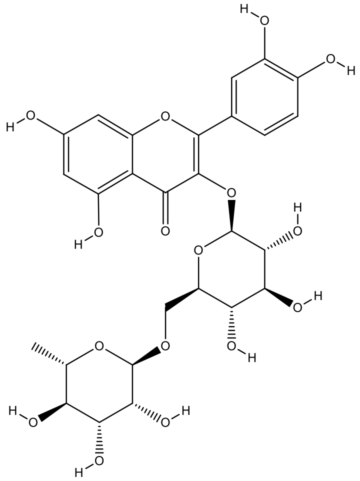<br>Rutin<br>153-18-4<br>SSYA10-001<br>See Adedeji et al. (2012)<br>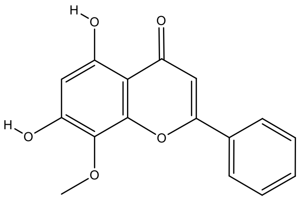<br>Wogonin<br>632-85-9
Jang et al. (2008) [59]	Aptamer ES15-1:<br>5’GAUAAUACGACUCACUAUAGGGUUCACUGCAGACUUGACGAAGCUUGCAGAAAAGGGGGAAGAAGAGGGUGAUUCAGGCGAGAGAAUGGAUCCACAUCUACGAAUUC3’<br><br>Aptamer ES15-2:<br>5’GAUAAUAGCACUCACUAUAGGGUUCACUGCAGACUUGACGAAGCUUCAGGGAGGAAAGGGGGAACCGACUCAAGAACUGUAGAGGGAAUGGAUCCACAUCUACGAAUUC3’<br><br>Aptamer ES15-3:<br>5’GAUAAUAGCACUCACUAUAGGGUUCACUGCAGACUUGACGAAGCUGGGCGGUCAAAGGAGAAGAAGAAAGAGAGAGCCCAGGGAAAUGGAUCCACAUCAAGGAAUC3’<br><br>Aptamer ES15-4:<br>5’GAUAAUAGCACUCACUAUAGGGUUCACUGCAGACUUGACGAAGCUUGGAGGGAAAAGGGGAAGCGGAAAGGUUAAGGAUGCGGAGGAAUGGAUCCACAUCUACGAAUUC3’<br><br>Aptamer ES15-5:<br>5’GAUAAUAGCACUCACUAUAGGGUUCACUGCAGACUUGACGAAGCUUGGUUAGGGGGAAAGGGGACCAGGUUCGCAGGAAAGCAGAGAAUGGAUCCACAUCUACGAAUUC3’<br><br>Aptamer ES15-6:<br>5’GAUAAUAGCACUCACUAUAGGGUUCACUGCAGACUUGACGAAGCUUGGAAGGGAGAGCGGGAACAAGGAGAAAGAGAAGGGGAAUCCAAUGGAUCCAUSCUACGAAUUC3’

	Underlined sequence: conserved motifs mostly reside at the loop region
Kao et. al. (2004) [55]	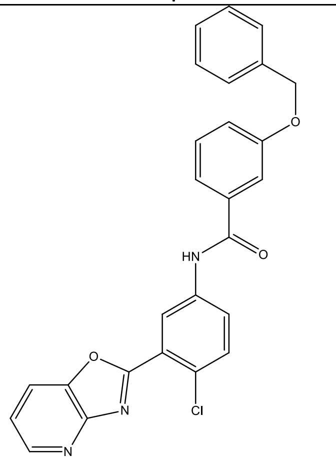<br>HE602<br>807367-44-8<br>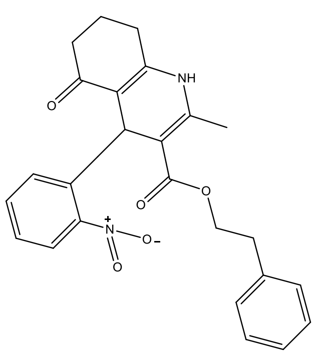<br>MP576<br>313244-76-7<br>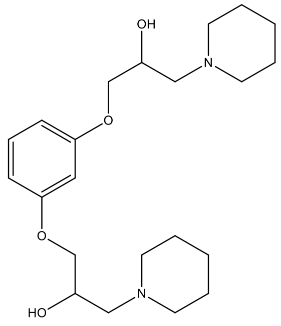<br>VE607<br>100434-29-5
Keum et al., (2013) [67]	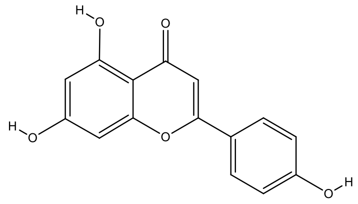<br>Apigenin<br>520-36-5<br>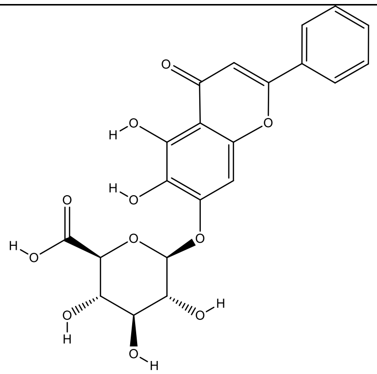<br>Baicalin<br>21967-41-9<br>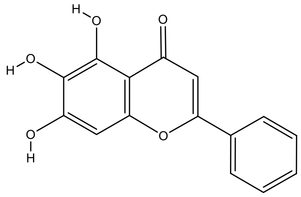<br>Baicalein<br>See Corona et. al. (2022)
	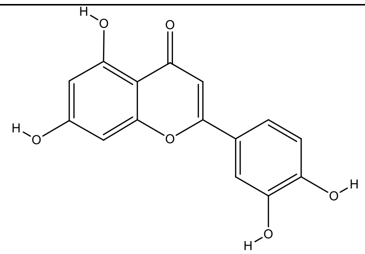<br>Luteolin<br>491-70-3<br>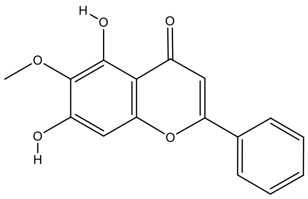<br>Oroxylin A<br>480-11-5<br>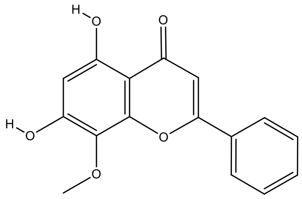<br>Wogonin<br>See Corona et. al. (2022)
Kim et al. (2011) [63]	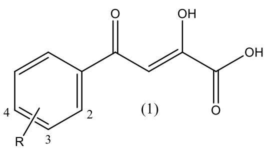<br>(1)<br>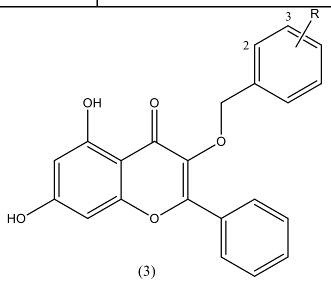<br>(3)

	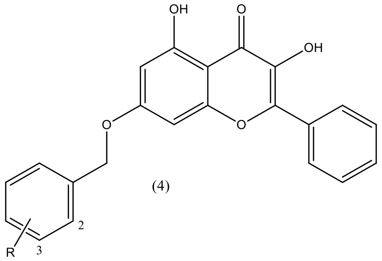 (4) 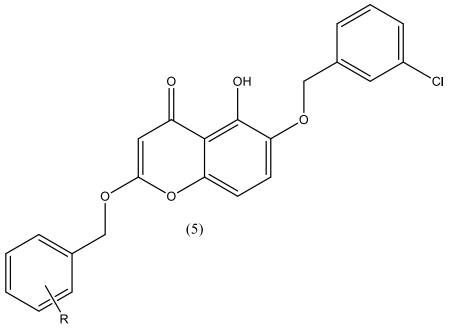 (5)		
	Derivative 1	1149590-14-6	R = 3-(4-chlorobenzyl-amino)
	Derivative 3	1236113-95-3	R = 3-NO_2
	Derivative 4	1160162-45-7	R = 3-CN
	Derivative 5a	1349177-50-9	R = H
	Derivative 5b	1349177-52-1	R = 3-Cl
	Derivative 5c	1349177-54-3	R = 4-Cl
	Derivative 5d	1349177-56-5	R = 3,5-di-Cl
	Derivative 5e	1349177-58-7	R = 3-I
	Derivative 5f	1349177-60-1	R = 4-I
	Derivative 5g	1349177-62-3	R = 3-CN
Kim et al. (2021) [76]	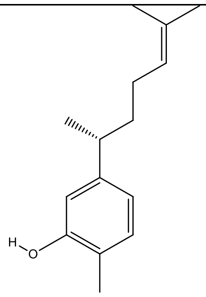 Xanthorrhizol	30199-26-9	
Lee et al. (2009a) [61]	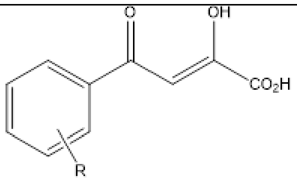		
	Derivative 1	251965-92-1	R = 4-OCH_2Ph
	Derivative 2	1149590-12-4	R = 4-OCH_2(4-ClPh)
	Derivative 3	251965-92-1	R = 3-OCH_2Ph
	Derivative 4	251965-93-2	R = 3-OCH_2(4-ClPh)
	Derivative 5	251966-04-8	R = 2-OCH_2Ph
	Derivative 6	1149590-13-5	R = 2-OCH_2(4-ClPh)
	Derivative 7	868616-28-8	R = 3-$NHCH_2$Ph
	Derivative 8	1149590-14-6	R = 3-$NHCH_2$(4-ClPh)

Lee et al. (2009b) [62]	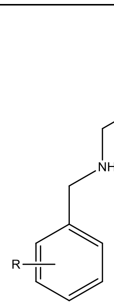<br>(2)<br>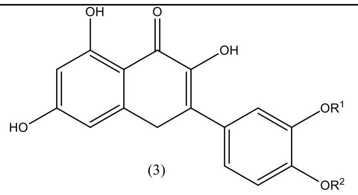<br>(3)<br>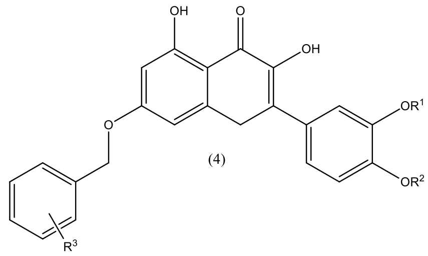<br>(4)<br>Derivative 2a 1187652-35-2 R = 4-Cl<br>Derivative 2b 1187652-33-0 R = 3-Cl<br>Derivative 2c 1187652-34-1 R = 3-CN<br>Derivative 3a 117-39-5 $R^1 = R^2 = H$<br>Derivative 3b 718627-91-9 $R^1 = R^2 = CH_2$<br>Derivative 4a 1187652-35-2 $R^1 = R^2 = H$, R^3 = 4-Cl<br>Derivative 4b 1187652-36-3 $R^1 = R^2 = H$, R^3 = 3-Cl<br>Derivative 4c 1187652-37 $R^1 = R^2 = H$, R^3 = 3-CN<br>Derivative 4d 1187652-38-5 $R^1 = R^2 = CH_2$, R^3 = 4-Cl<br>Derivative 4e 1187652-39-6 $R^1 = R^2 = CH_2$, R^3 = 3-Cl<br>Derivative 4f 1187652-40-9 $R^1 = R^2 = CH_2$, R^3 = 3-CN
Lee et al. (2017) [70]	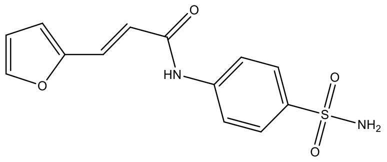<br>(E)-3-(furan-2-yl)-N-(4-sulfamoylphenyl) acrylamide<br>(FSPA)<br>2482787-66-4
Lee et. al. (2016) [69]	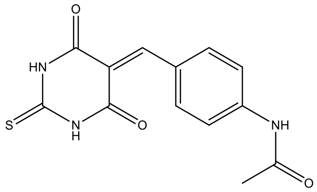<br>*N*-(4-((4,6-dioxo-2-thioxotetrahydropyrimidin-5(*2H*)-ylidene)methyl)phenyl)acetamide<br>(DTPMPA)<br>260410-16-0

Lu et. al. (2022) [81]	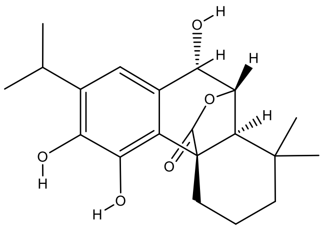<br>Rosmanol<br>80225-53-2<br>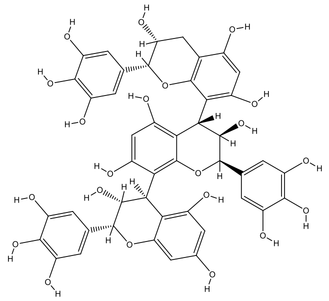<br>Katacine<br>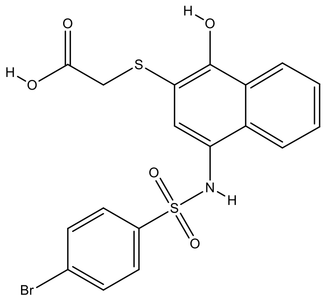<br>UMI-77<br>518303-20-3<br>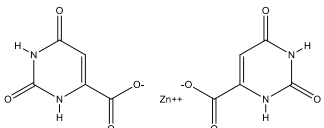<br>Zinc orotate<br>68399-76-8<br>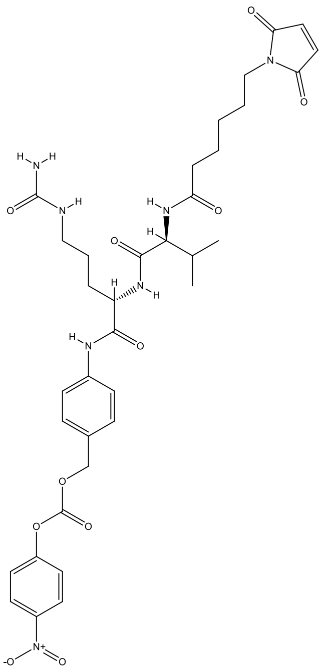<br>Mc-Val-Cit-PABC-PNP<br>159857-81-5<br>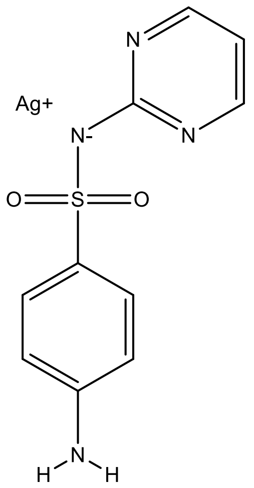<br>Silver sulfadiazine<br>22199-08-2

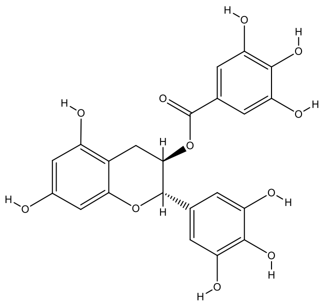

(-)-Gallocatechin gallate
4233-96-9

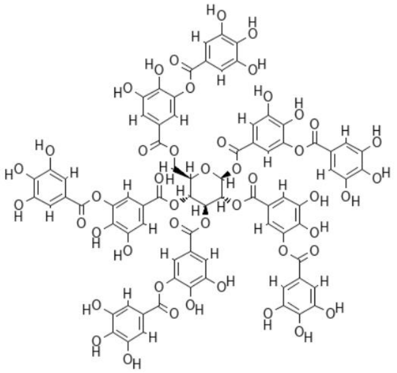

Tannic acid
1401-55-4

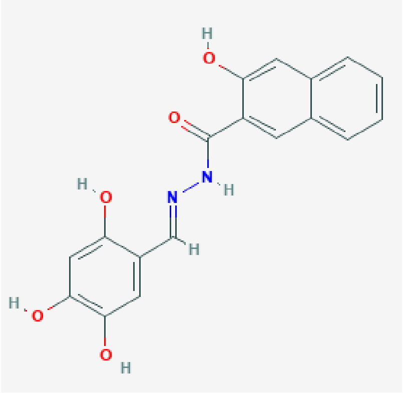

Dyngo-4a
1256493-34-1

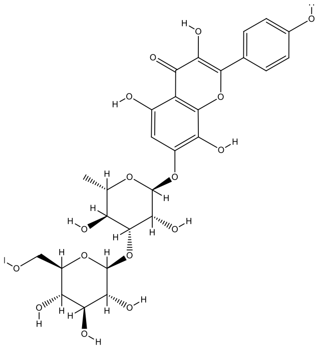

Rhodiosin
86831-54-1

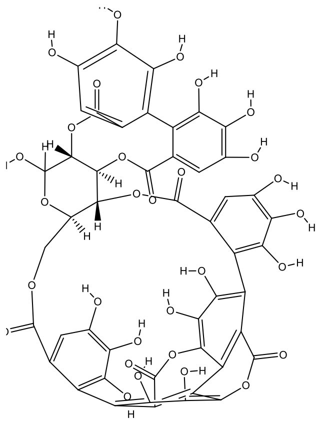

Punicalagin
65995-63-3

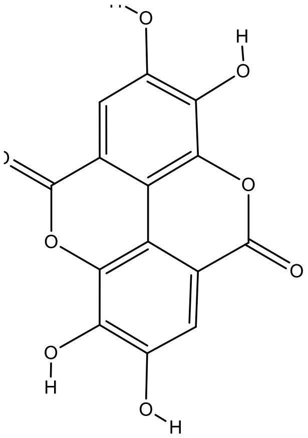

Ellagic acid
476-66-4

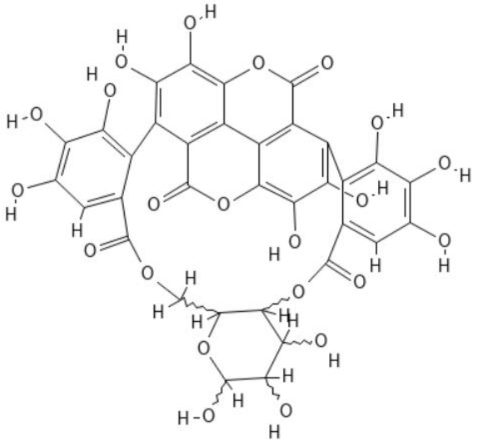

Punicalin
65995-64-4

Mehyar et al. (2021a) [26]<br>Mehyar et al. (2021b) [27]	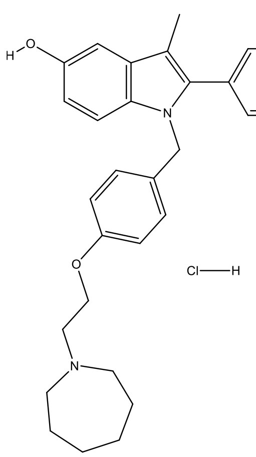<br>Bazedoxifene HCl<br>198480-56-7<br>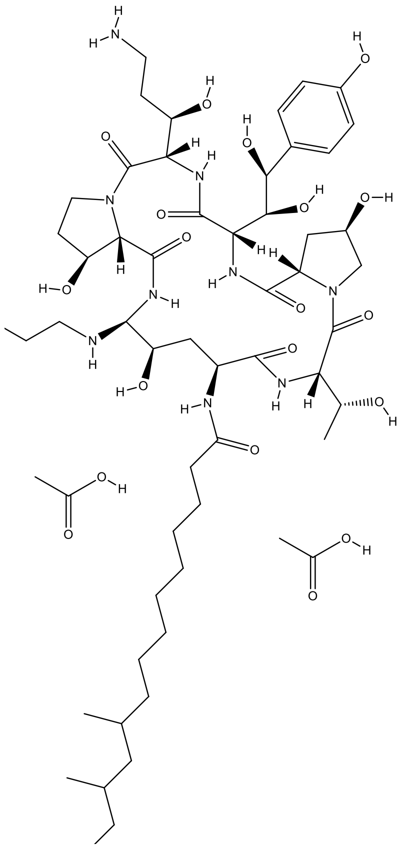<br>Caspofungin acetate<br>179463-17-3<br>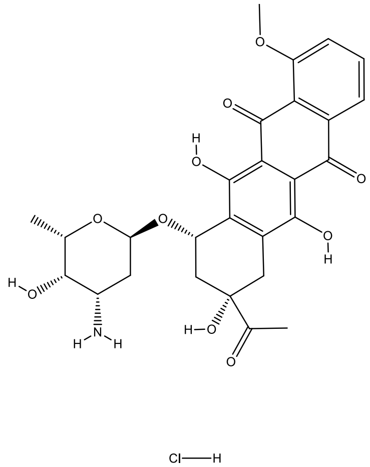<br>Daunorubicin HCl<br>20830-81-3<br>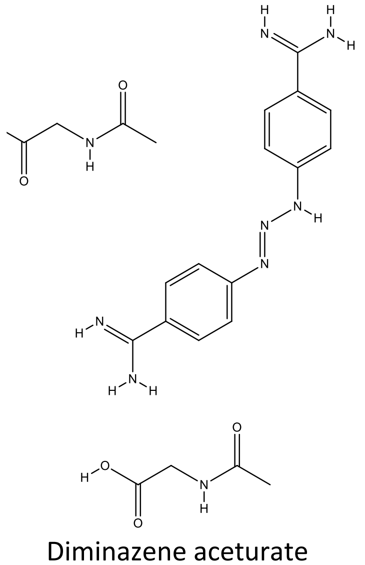<br>Diminazene aceturate<br>908-54-3<br>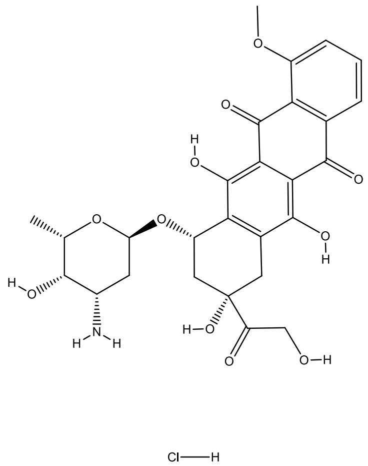<br>Doxorubicin HCl<br>23214-92-8<br>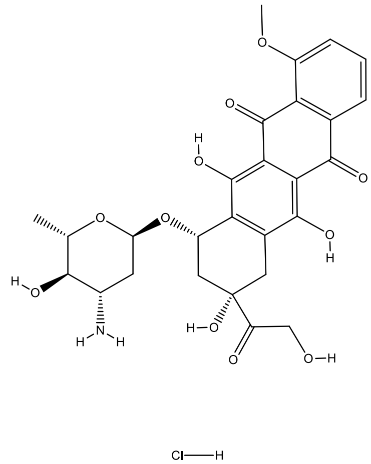<br>Epirubicin HCl<br>56420-45-2

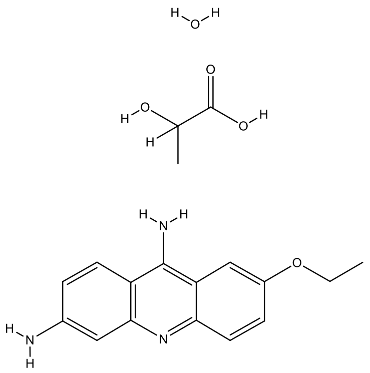

Ethacridine lactate monohydrate
6402-23-9

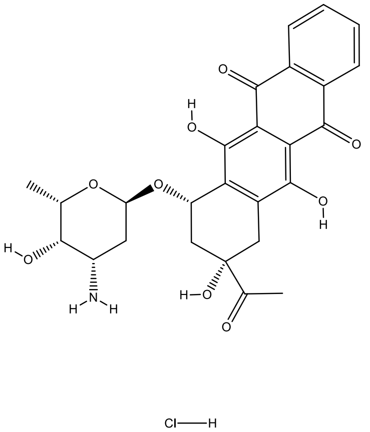

Idarubicin HCl
58957-92-9

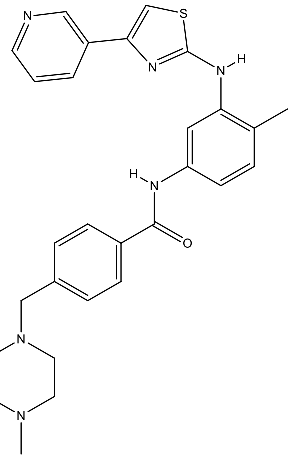

Masitinib
790299-79-5

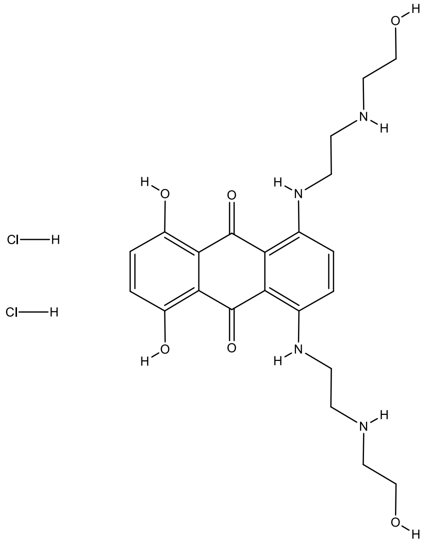

Mitoxantrone 2HCl
65271-80-9

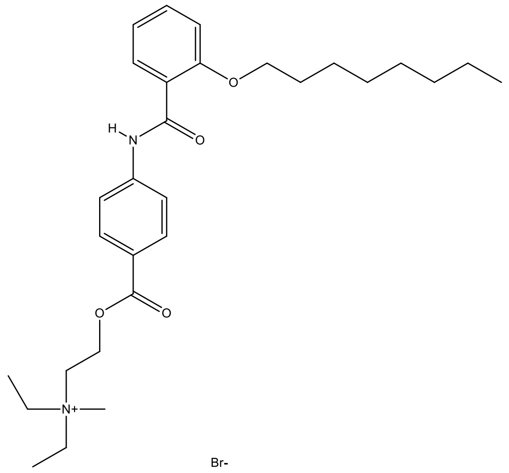

Otilonium bromide
26095-59-0

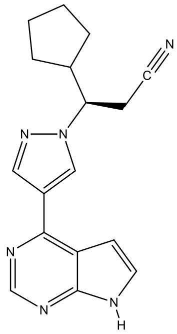

Ruxolitinib
941678-49-5

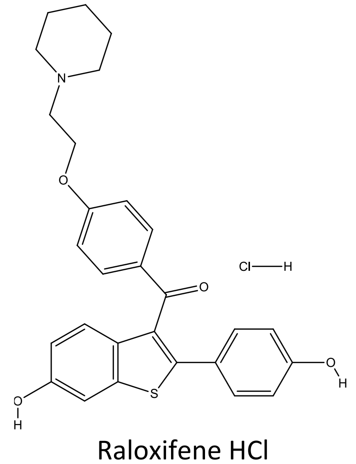

Raloxifene HCl
82640-04-8

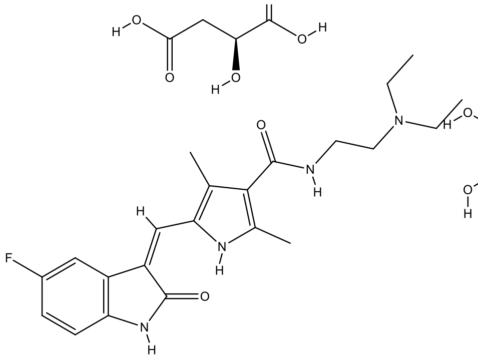

Sunitinib malate
341031-54-7

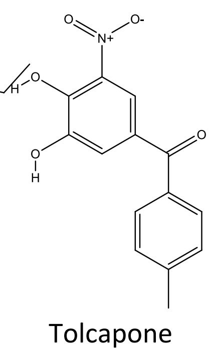

Tolcapone
134308-13-7

Mehyar et al. (2021b) [27]	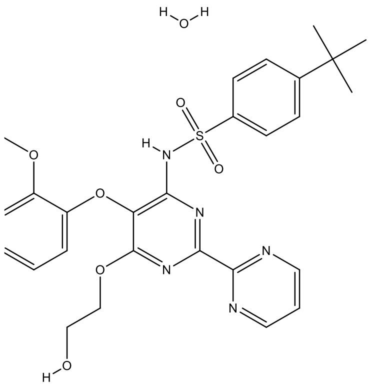<br>Bostenian hydrate<br>157212-55-0<br>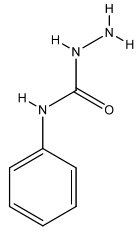<br>Chondroitin sulphate<br>537-47-3<br>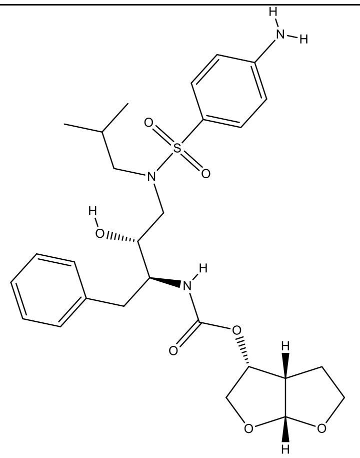<br>Darunavir<br>206361-99-1<br>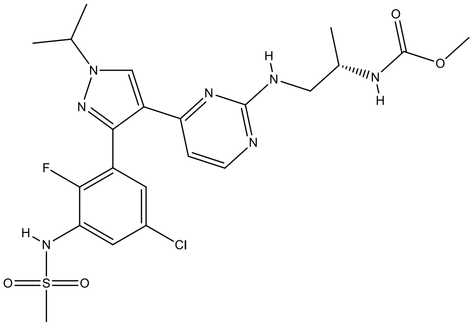<br>Encorafenib<br>1269440-17-6<br>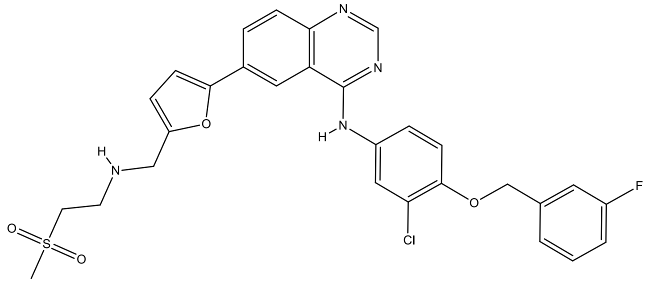<br>Lapatinib<br>231277-92-2<br>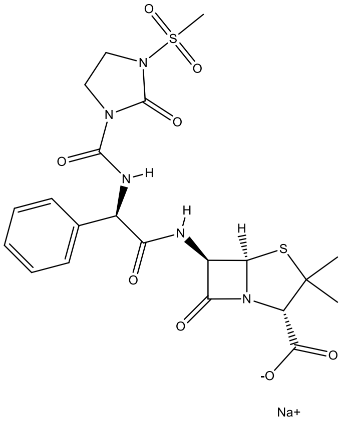<br>Mezlocillin sodium<br>59798-30-0<br>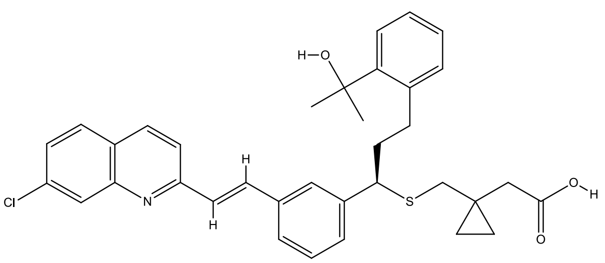<br>Montelukast<br>158966-92-8

<table>
<tr>
<td></td>
<td colspan="2">
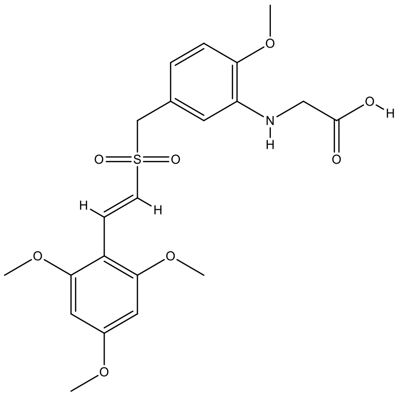

Rigosertib
592542-59-1

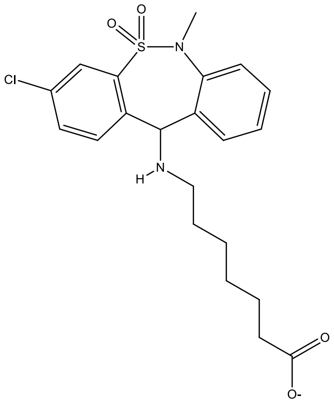

Tianeptine sodium
30123-17-2

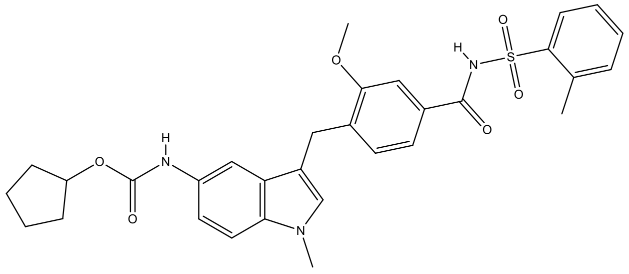

Zafirlukast
107753-78-6
</td>
</tr>
<tr>
<td>Nizi et. al., 2022 [79]</td>
<td>
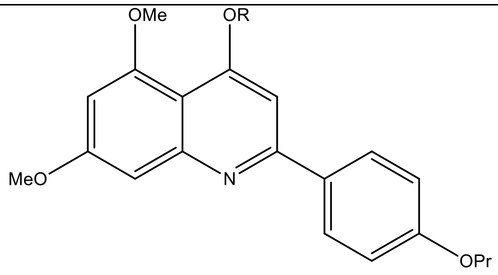

6f
R =

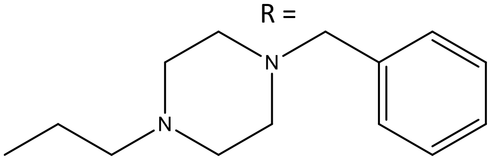

6g
R =

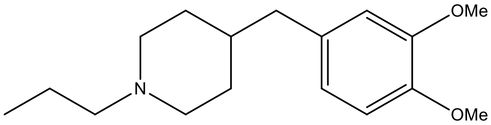

</td>
<td>
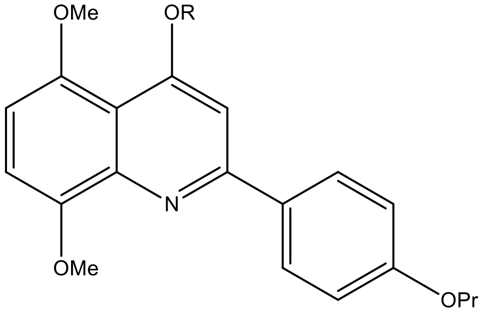

7k
R =

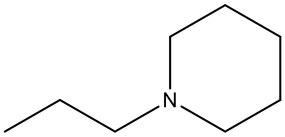

</td>
</tr>
</table>

	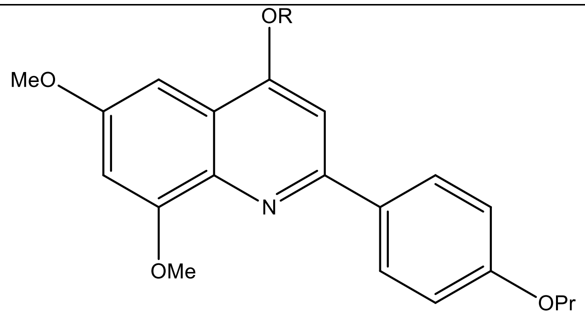<br>9j<br>R =<br>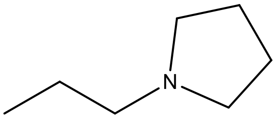
Romeo et. al. (2022) [82]	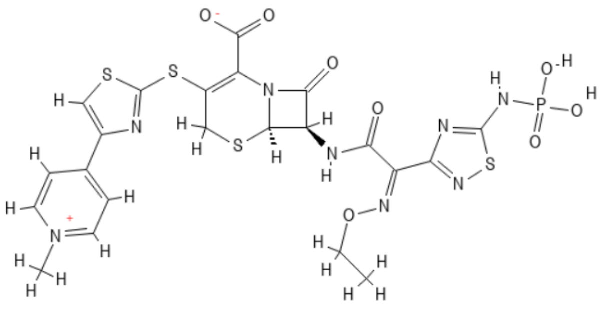<br>Ceftaroline fosamil<br>229016-73-3<br>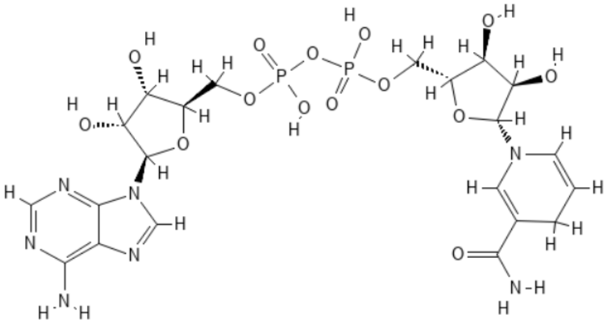<br>NADH<br>58-68-4<br>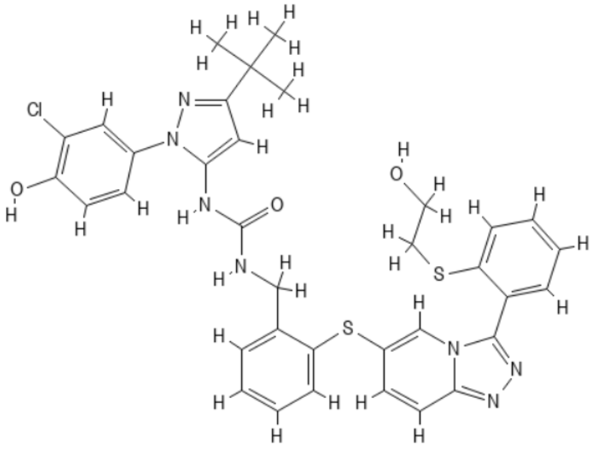<br>PF-03715455<br>1056164-52-3<br>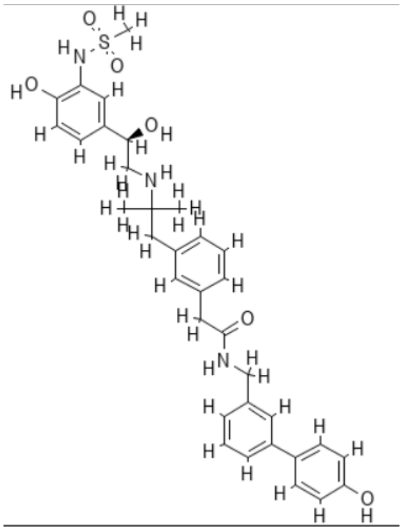<br>PF-00610355<br>862541-45-5

	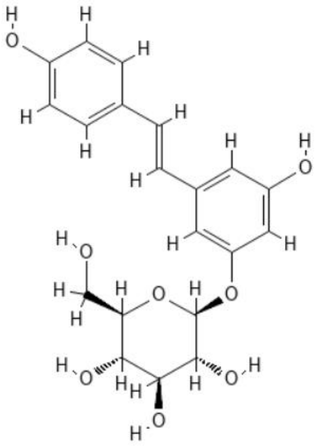<br>Polydatin<br>27208-80-6	
Shu et al. (2020) [72]	Bismuth citrate (BC): See Yang et al. (2007a)<br>Colloidal bismuth subcitrate: See Yang et al. (2007a)<br>Ranitidine bismuth citrate: See Yang et al. (2007a)	
Shum and Tanner (2008) [60]	Aptamer NG1	--------GTGTGAGGGTGAG-ATGTGTGTG TATTTGTC-----
	Aptamer NG2	--------GTGTTTACGTGCC-ATATTTGTGTGGTGGTT----
	Aptamer NG3	--------AGGTGGGCATGATTGTGTGTTTGTGTCGGT-----
	Aptamer NG4	-ACCTGCTGTGTTGG-ATGT----GTTGGTGGC AGC--------
	Aptamer NG5	-ATGTGGTGTGTTAGTGTGT----GTTCGTGGTT----------
	Aptamer NG6	-------ATAGTTGGTGTGTT--- GGTACTGCATT---------
	Aptamer NG7	--AATGTAGTATGGATGGATT---GTTAGTTCGGTC---------
	Aptamer NG8	-ATGTTGGTAGTTGGCTTGT----GTTCGTGTG TT---------
	Aptamer NG9	-------TGTGTAGGATTG-------TAGTGTGTTTGTGTGACT
	Aptamer NG10	------GAGGGTAGGACTGG----TCTAGTGTGTT-GTGTT---
	Aptamer NG11	-----GATGTGTGGACTGTA----CTCAGCTGGTTGGTT-----
	Aptamer NG12	GCTCTGCTGTGTTGGATTG-----TATGCTGTGTT---------
	Aptamer NG13	-------ACGCTTACTTTATGTTGTTTGCCGGAT---------
	Aptamer NG14	---------AGCGAGATTGTCACACGTGCTGAATAC ATC-----
	Aptamer G1	--AACTTGGG-GTGGGTGTGTTGTGTACGGGC----
	Aptamer G2	--GGCTTGTG-GGTGGTATCTGTGGTGTGTGCT---
	Aptamer G3	----CGTGGGTGGTTGTGTCGGGGCGATGGGTT---
	Aptamer G4	ATGGCATGTGTTTGGCG-TGGATCGTGTGGC-----
	Aptamer G5	--AGCGGGCATATGGTGGTGGGTGGTATGGTC----
	Aptamer G6	-AAGGGTGGA-AAGTTGGGGGGTGTAGTGTTC----
	Aptamer G7	-----ATGCC-GCGTTGGGGAGTGGTGTTGGCTGGT
	Aptamer G8	------AATGGAGTATGGATGGATTGCTAGTTCGGC
	Aptamer G9	--GCTGGCCGGGATATGGTATGTTTGGCAGTT----

<table>
<tr><td>Tanner et al. (2005) [56]</td><td colspan="2">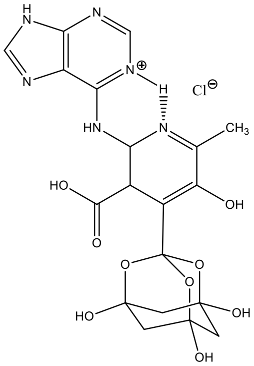
Adeninonananin
858956-99-7
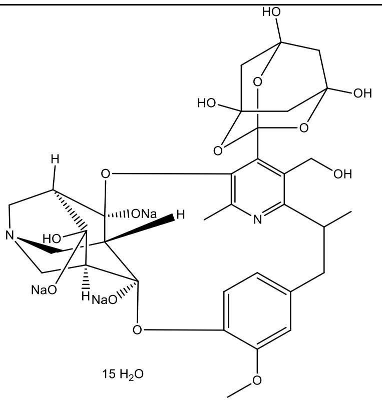
Ansabananin
858956-97-5P
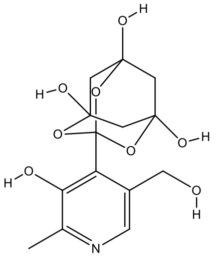
Bananin
665026-57-3
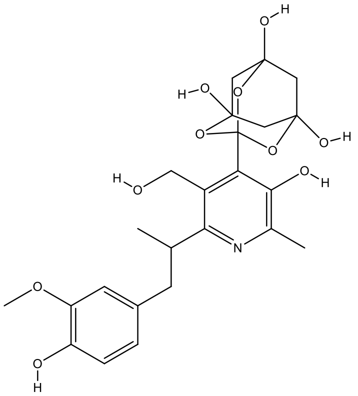
Eubananin
858956-98-6P
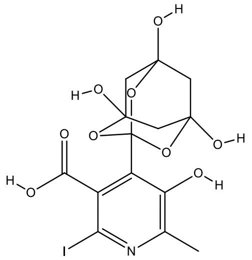
Iodobananin
858956-95-3
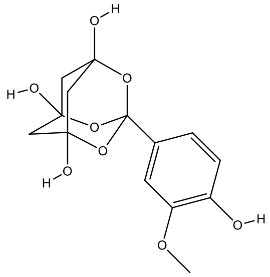
Vanillinbananin
858956-96-4P</td></tr>
<tr><td>White et al. (2020) [33]</td><td>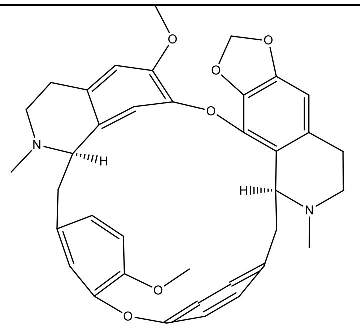
Cepharanthine
481-49-2</td><td>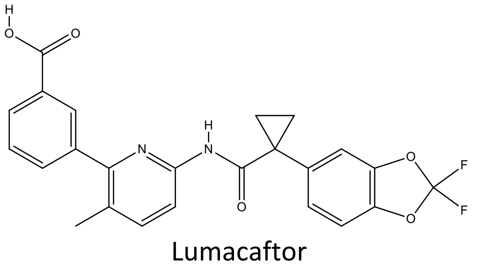
Lumacaftor
936727-05-8</td></tr>
</table>

	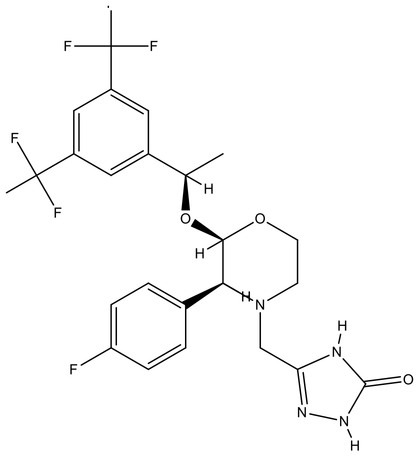<br>Emend<br>170729-80-3	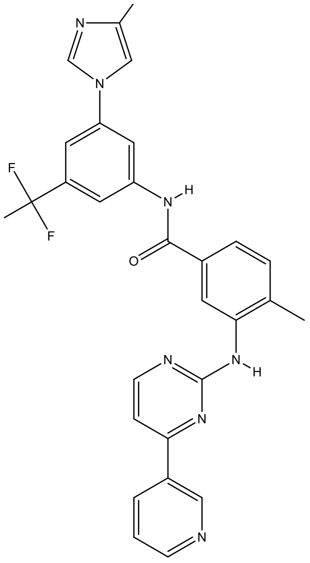<br>Nilotinib<br>641571-10-0
	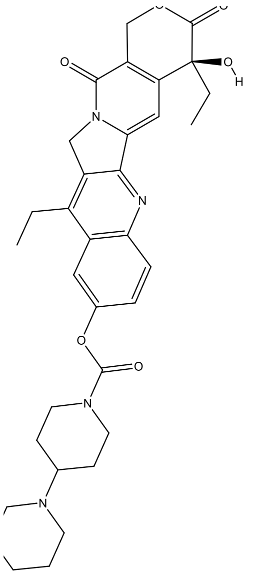<br>Irinotecan<br>97682-44-5	Enjuvia<br>Structure and CAS not available

	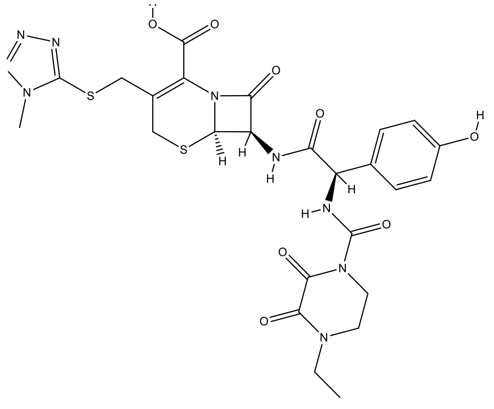<br>Cefoperazone<br>62-893-19-0<br>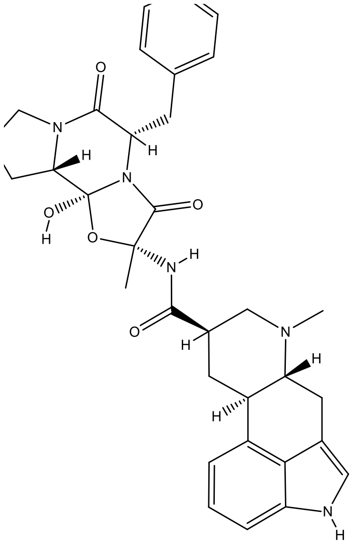<br>Dihydroergotamine<br>511-12-6<br>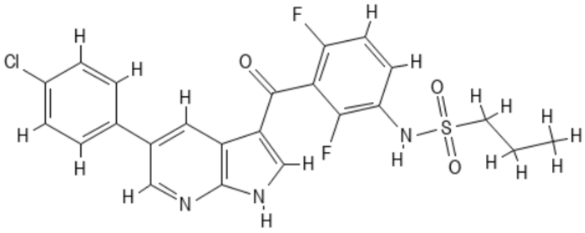<br>Zelboraf<br>918504-65-1
Yang et al. (2007a) [57]	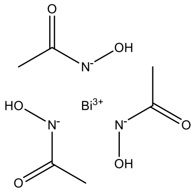<br>Bismuth acetohydroxamate<br>(CAS number not available)<br>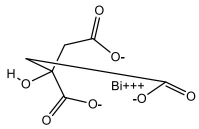<br>Bismuth citrate<br>813-93-4<br>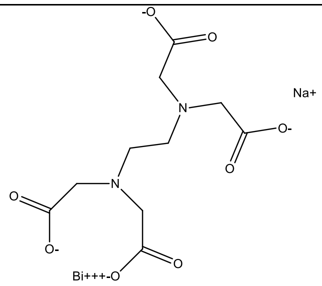<br>Bismuth ethylenediaminetetraacetate<br>12558-49-5<br>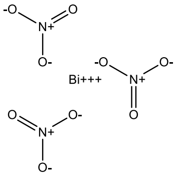<br>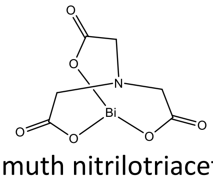<br>Bismuth nitrilotriacetate

	Bismuth nitrate<br>10361-44-1	16073-78-2<br><br>Ranitidine bismuth citrate<br>see Yuan et al. (2020)	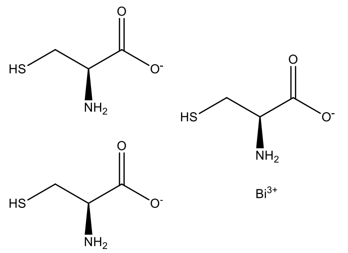<br><br>Bismuth tricysteine complex<br>90901-09-0
Yang et al. (2007b) [58]	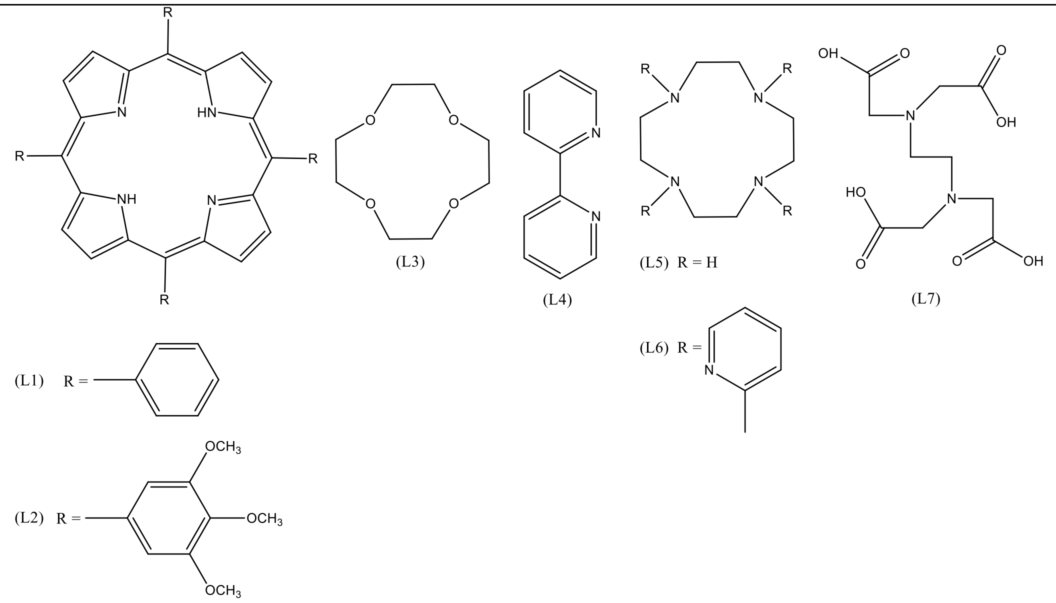 		
	Complex 1	960068-51-3	([Bi(**L1**) (NO3)]· H2O<br>**L1** = 5,10,15,20-tetraphenyl-21H,23H-porphine
	Complex 2	960068-57-9	[Bi(**L2**) - (NO3)]· H2O<br>**L2** = 5,10,15,20-tetra(1,2,3-trimethoxyphenyl)-21H,23H-porphine
	Complex 3	139943-85-4	([Bi (NO3)3 (**L3**)]<br>**L3** = 12-crown-4
	Complex 4	207846-38-6	([BiCl3-(**L4**)1.5]<br>**L4** = 2,2'-bipyridine
	Complex 5a	188713-30-6	([Bi(**L5**) (H2O) (ClO4)3]<br>**L5** = 1,4,7,10-tetraazacyclododecane
	Complex 5b	185130-32-9	([Zn(**L5**) (H2O)] (ClO4)2)<br>**L5** = 1,4,7,10-tetraazacyclododecane
	Complex 6	145773-30-4	([Bi (NO3)4(**L6**)]<br>**L6** = 1,4,7,10-tetrakis(2-pyridylmethyl)-1,4,7,10-tetraazacyclododecane)
	Complex 7	94919-10-5	([Bi(**L7**)]<br>**L7** = ethylenediamine-tetraacetic acid

Yazdi et. al. (2022) [80]	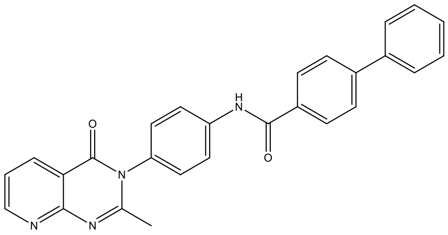<br>C1<br>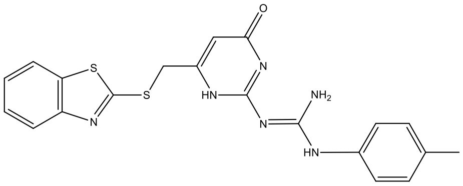<br>C2<br>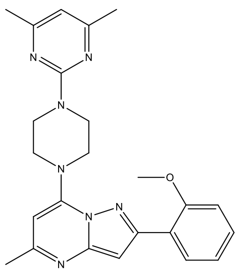<br>C3<br>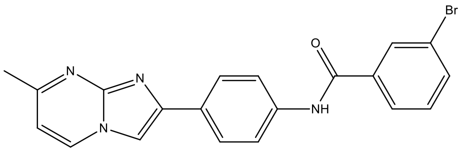<br>C4<br>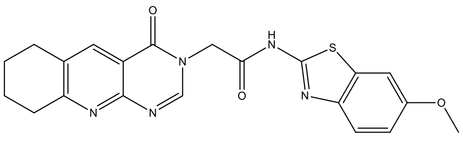<br>C5<br>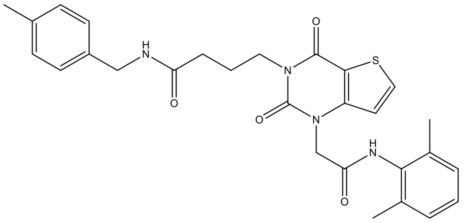<br>C6<br>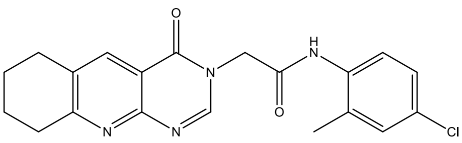<br>C7<br>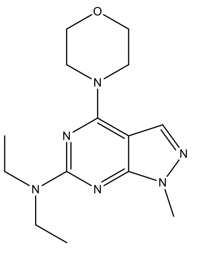<br>C8

Yu et. al. (2012) [66]	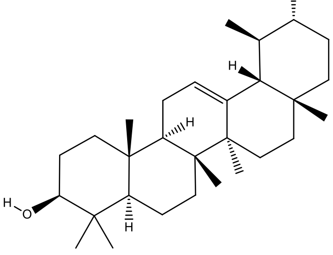<br>α-Amyrin<br>638-95-9<br>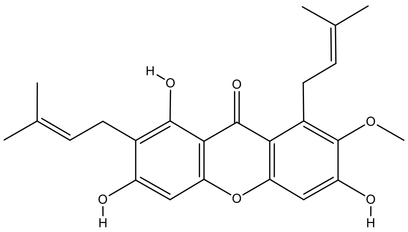<br>α-Mangostin<br>6147-11-1<br>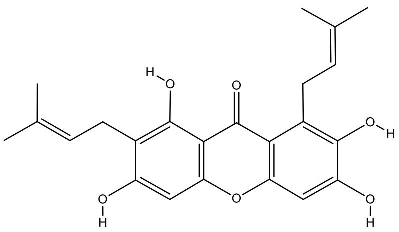<br>γ-Mangostin<br>31271-07-5<br>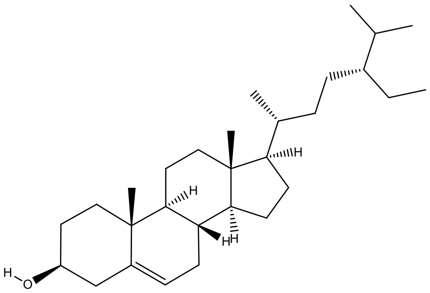<br>β-Sitosterol<br>83-46-5<br>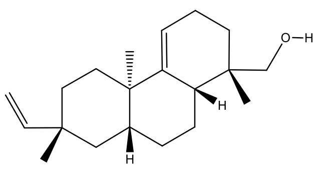<br>19-Hydroxy-1(10),15-rosadiene<br>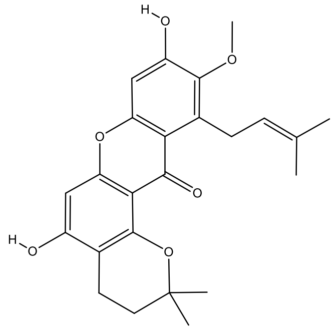<br>1-Isomangostin<br>19275-44-6<br>24-Methyl-S«-lanosta-9 (11),25-dien-3oi-one<br>(No CAS, no structure)<br>3-*O*-Angeloylh amaudol<br>(No CAS, no structure)<br>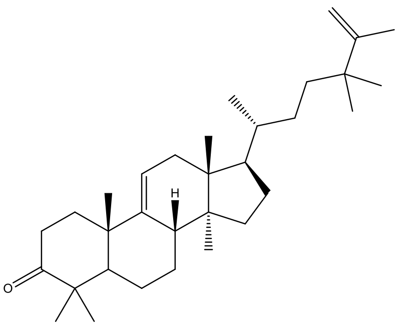<br>24,24-Dimethylla nosta-9(11),25-dien-3-one<br>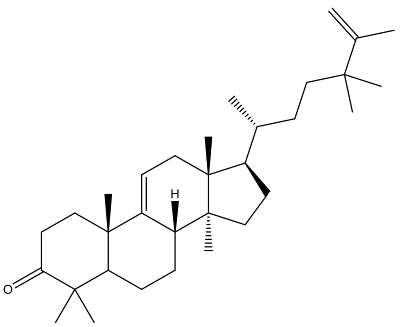<br>24-Methyllanosta-9(11),25-dien-3-one<br>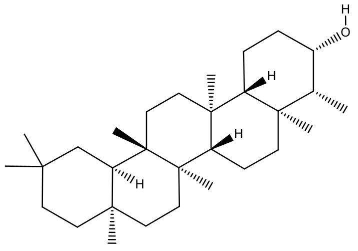<br>3β-Friedelanol<br>16844-71-6<br>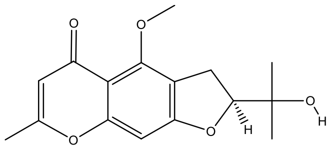<br>5-*O*-Methylvisamminol<br>80681-42-1

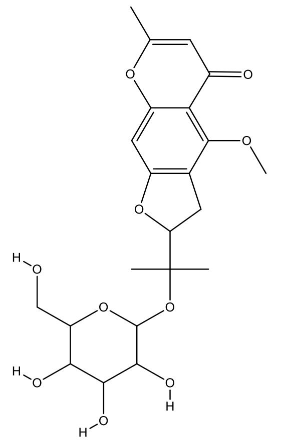

4’-*O*-β-D-glucosyl-5-O-
methylvisamminol

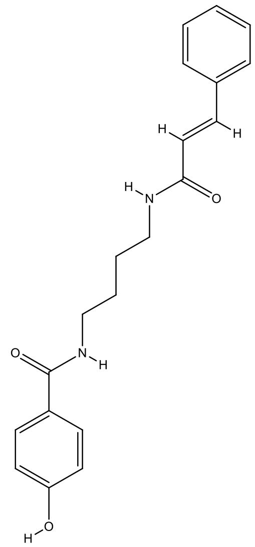

4-Hydroxypyramidatine

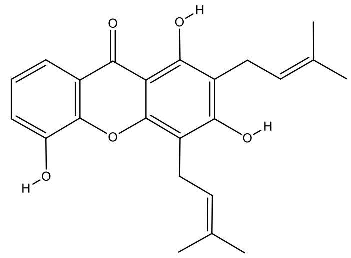

8-Deoxygartanin

33390-41-9

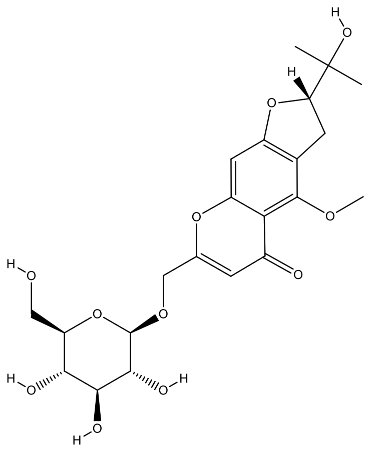

Prim-*O*-Glucosylcimifugin
80681-45-4

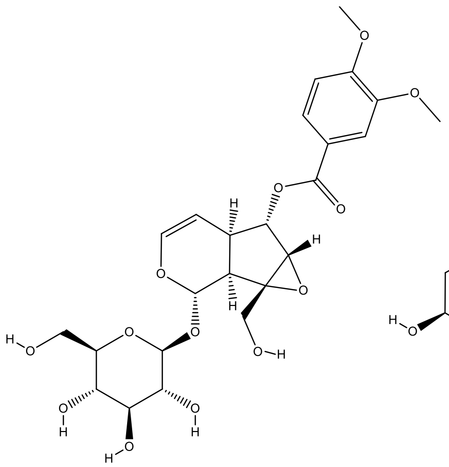

6-*O*-Veratroyl Catalpol
56973-43-4

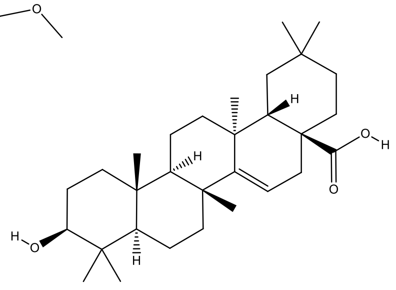

Aleuritolic acid
26549-17-7

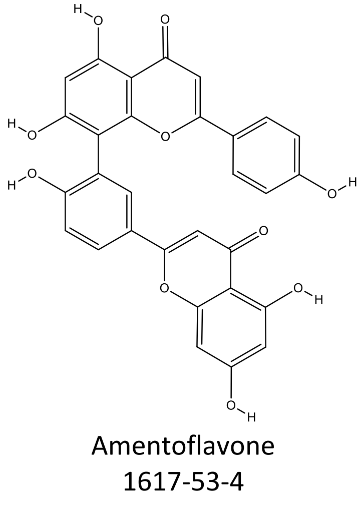

Amentoflavone
1617-53-4

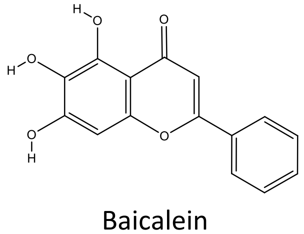

Baicalein
See Corona et. al. (2022)

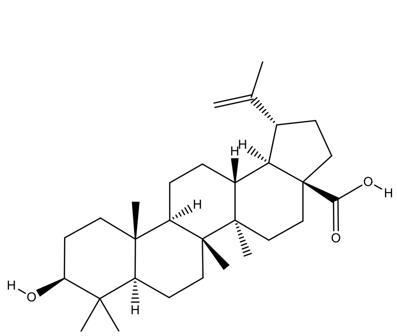

Betulinic acid
472-15-1

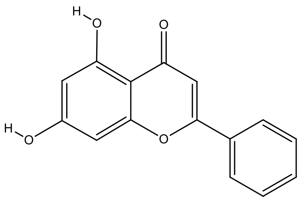

Chrysin
480-40-0

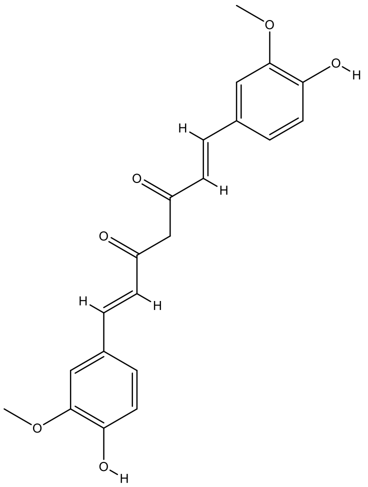

Curcumin
458-37-7

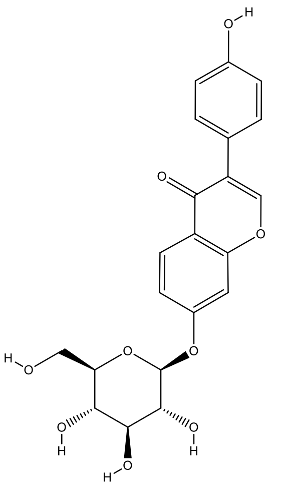

Daidzin
552-66-9

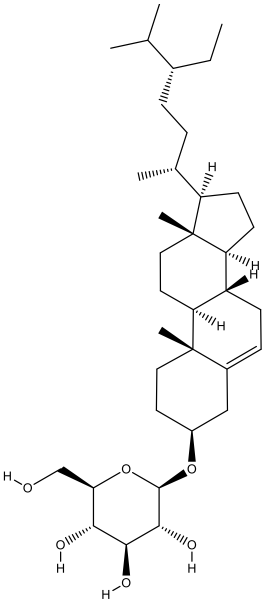

Daucosterol
474-58-8

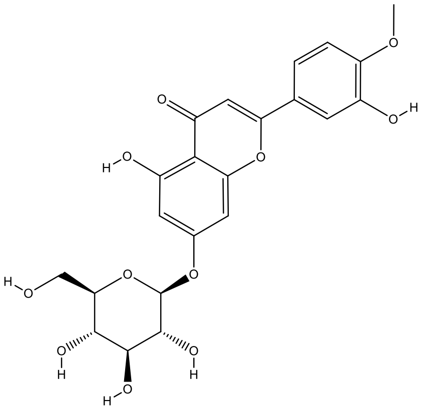

Diosmetin-7-*O*-Glc
20126-59-4

Diosmetin-7-O-Glc-Xy
(No CAS, no structure)

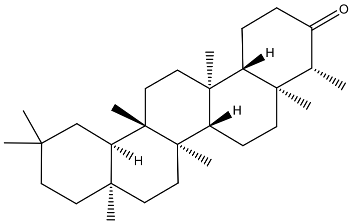

Friedelin
559-74-0

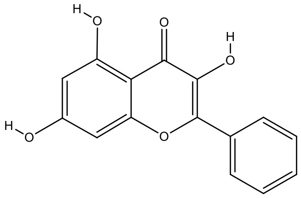

Galangin
548-83-4

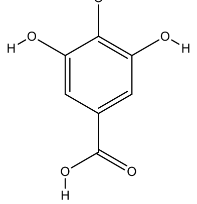

Gallic acid
(See Corona et al., 2022)

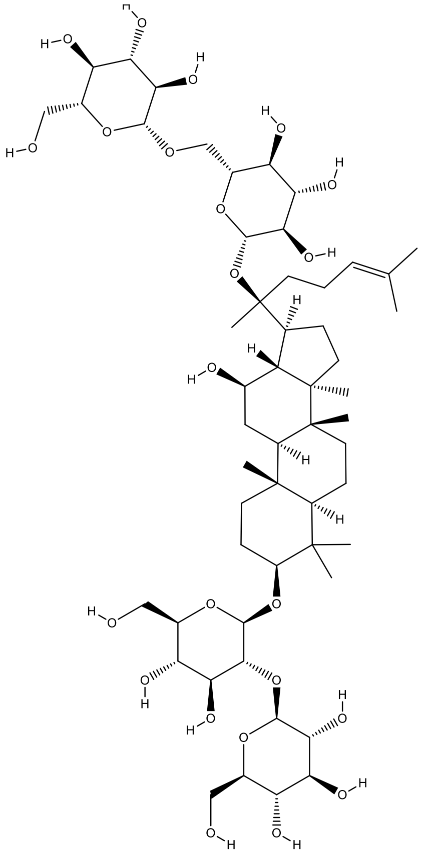

Ginsenoside Rb1
41753-43-9

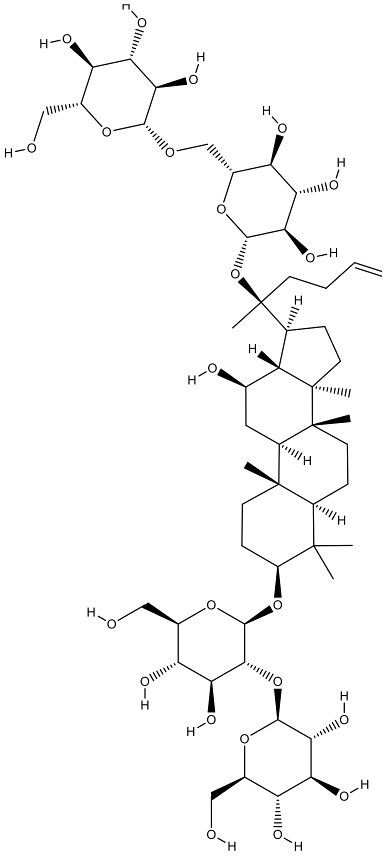

Gypenoside XVII
41753-43-9

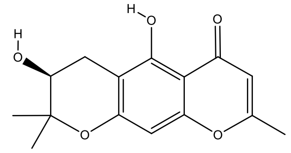

Hamaudol
735-46-6

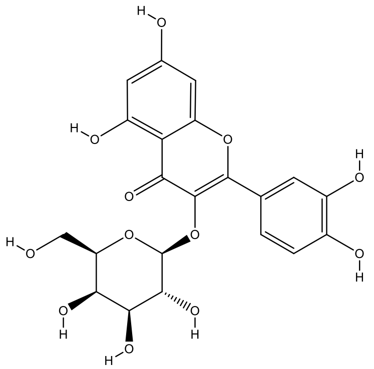

Hyperoside
482-36-0

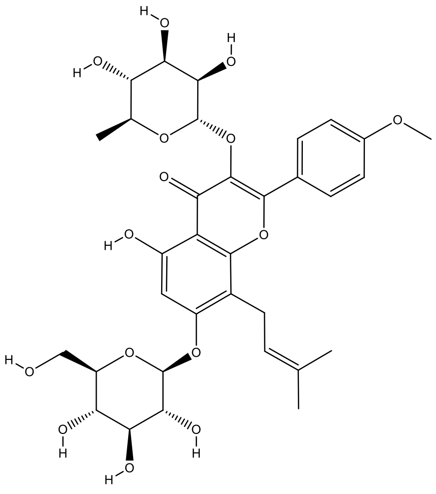

Icariin
489-32-7

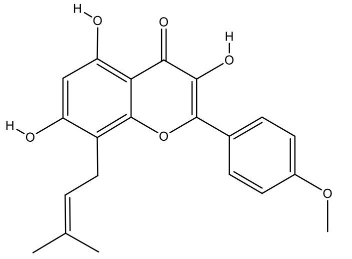

Icaritin
118525-40-9

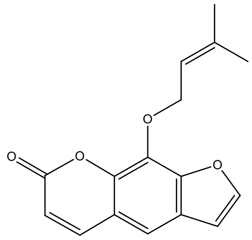

Imperatorin
482-44-0

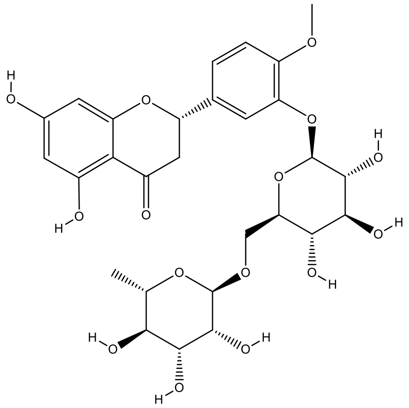

Isohesperidin
82350-96-7

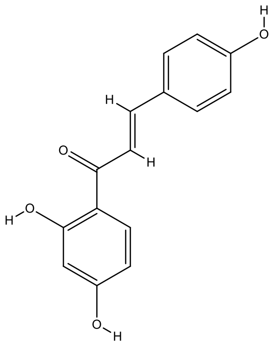

Isoliquiritigenin
961-29-5

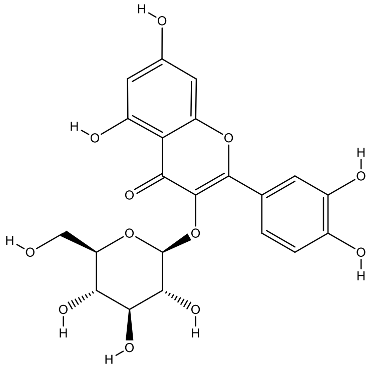

Isoquercetin
482-35-9

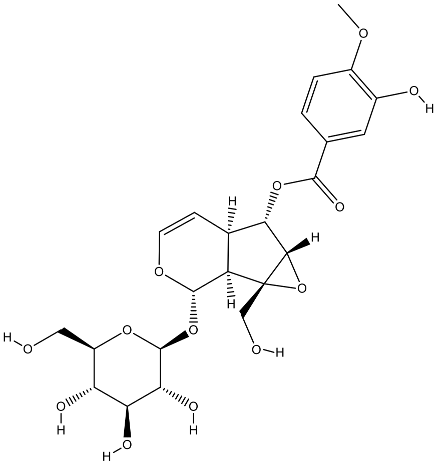

Isovanillyl Catapol

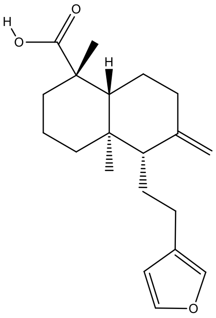

Lambertiainc acid

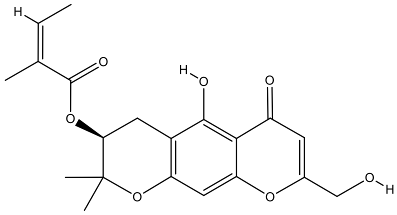

Ledebouriellol

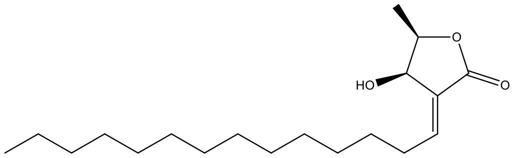

Marliolide

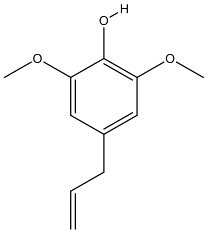

Methoxyeugenol
6627-88-9

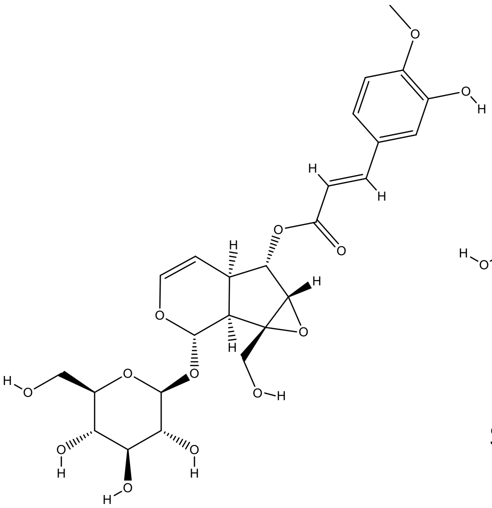

Minecoside
51005-44-8

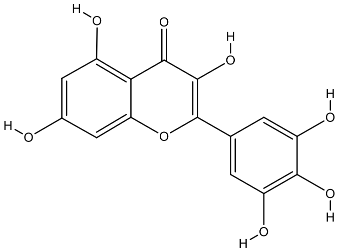

Myricetin
See Zeng et al. (2021)

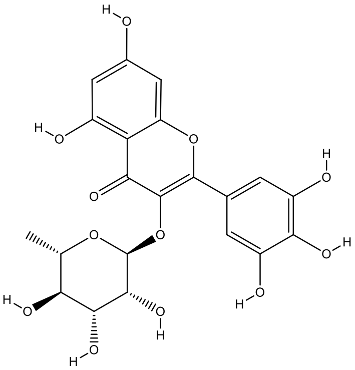

Myricitrin
17912-87-7

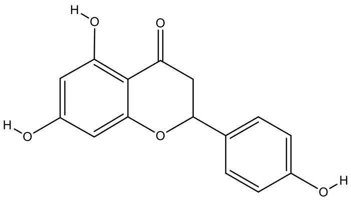

Naringenin
67604-48-2

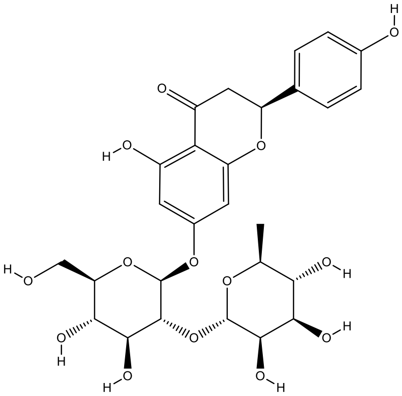

Naringin
10236-47-2

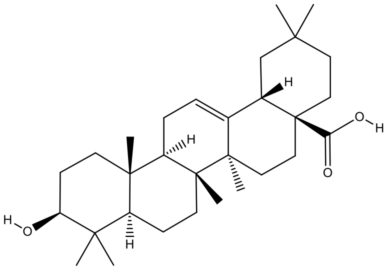

Oleanolic acid
508-02-1

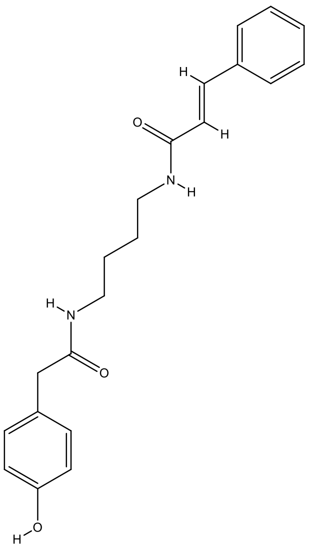

Perviridamide

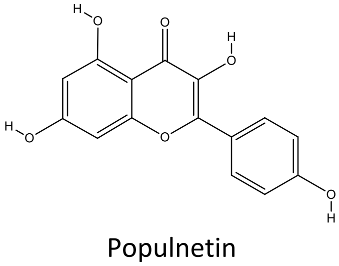

Populnetin

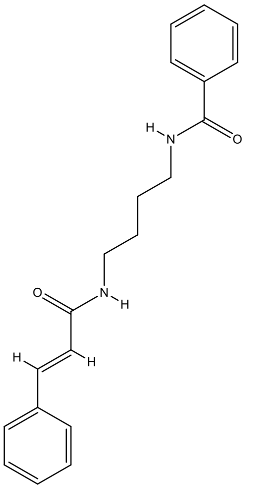

Pyramidatine
64223-54-7

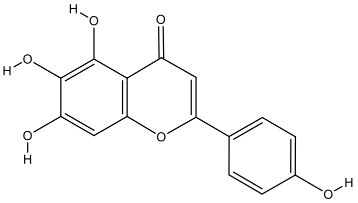

Scutellarein
529-53-3

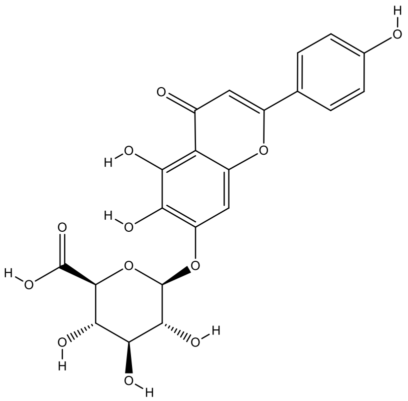

Scutellarin
27740-01-8

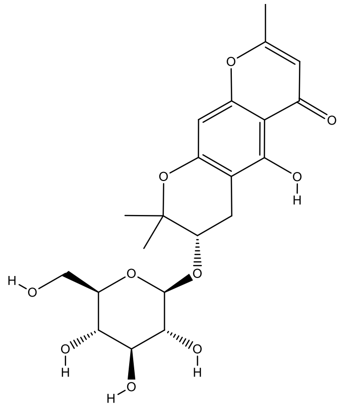

Sec-O-Glucosylha maudol
80681-44-3

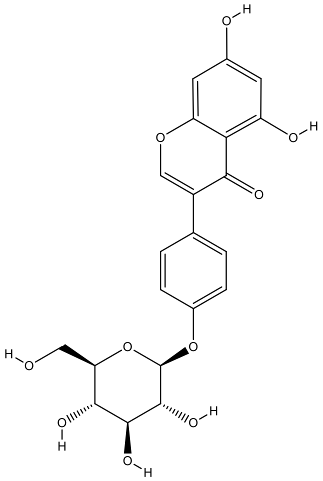

Sophoricoside
152-95-4

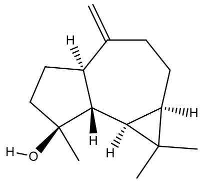

Spatulenol
6750-60-3

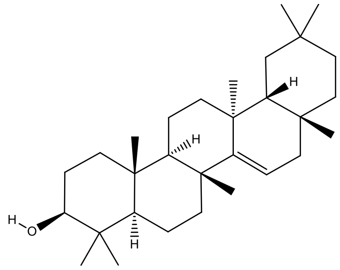

Taraxerol
127-22-0

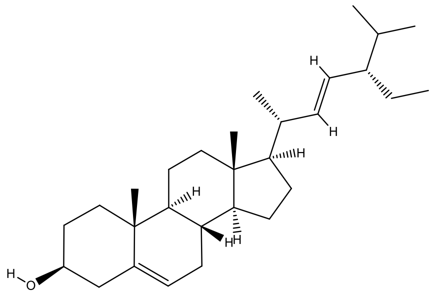

Stigmasterol
83-48-7

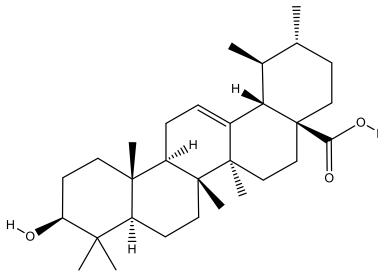

Ursolic acid
77-52-1

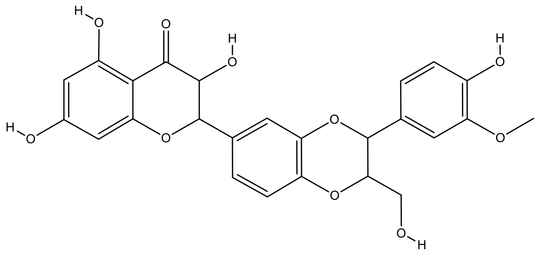

Silymarin
65666-07-1

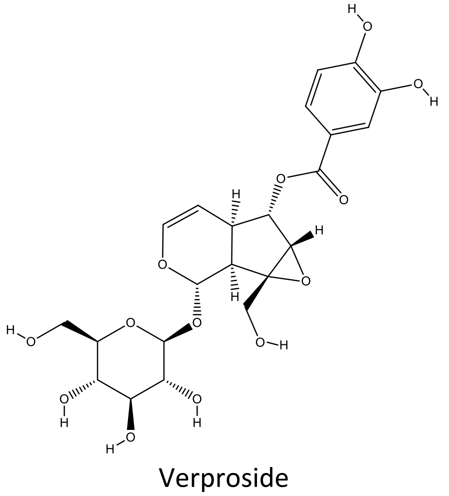

Verproside
50932-20-2

Yuan et al. (2020) [73]	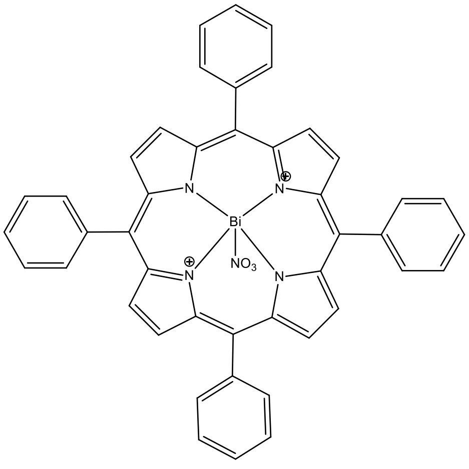<br>Bi(TPP)<br>Bi (TPP)<br>TPP = tetraphenylporphyrinate<br>268748-52-3<br>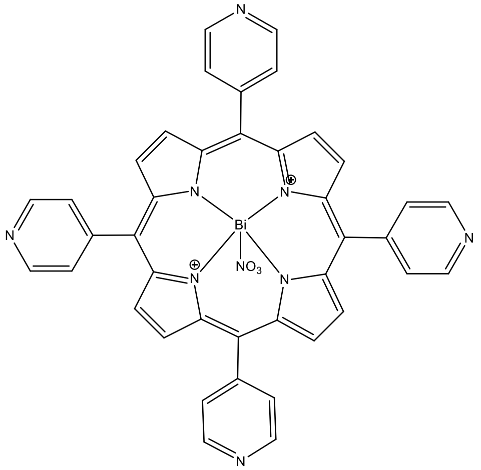<br>Bi(TPyP)<br>Bi (TPyP)<br>TPyP = tetra(4-pyridyl) porphyrin)<br>2493360-14-6
	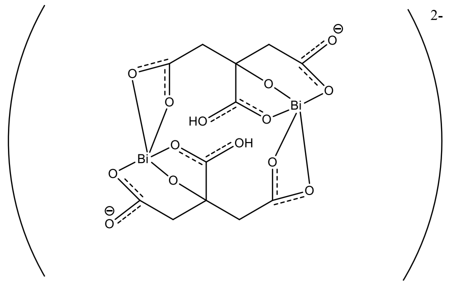<br>Colloidal bismuth subcitrate<br>57644-54-9<br>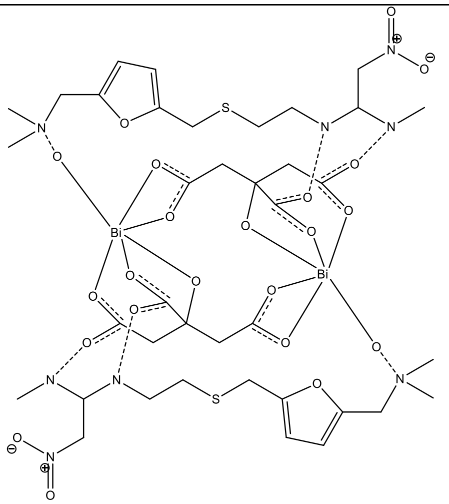<br>Ranitidine bismuth citrate<br>128345-62-0
Yuan et al. (2021) [74]	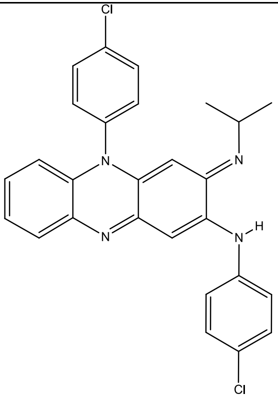<br>Clofazimine<br>2030-63-9

<table>
<tr><td rowspan="2">Zaher et al.
(2020) [71]</td><td rowspan="2">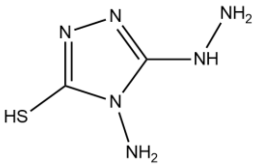
4-Amino-5-hydrazino-4H-1,2,4-triazole-3-thiol
1750-12-5</td><td>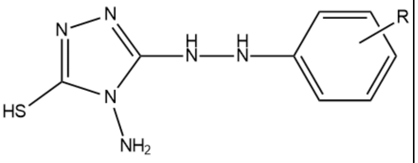
Derivatives 1-8</td><td>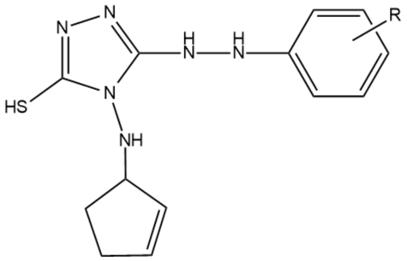
Derivatives 9-16</td></tr>
<tr><td>1 and 9 R = 2-F
2 and 10 R = 4-F
3 and 11 R = 2-Cl
4 and 12 R = 4-Cl</td><td>5 and 13 R = 2-Br
6 and 14 R = 4-Br
7 and 15 R = 2-I
8 and 16 R = 4-I</td></tr>
<tr><td>Zeng et al.
(2021) [77]</td><td colspan="3">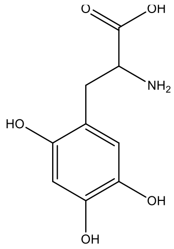
6-Hydroxy-DL-DOPA
21373-30-8
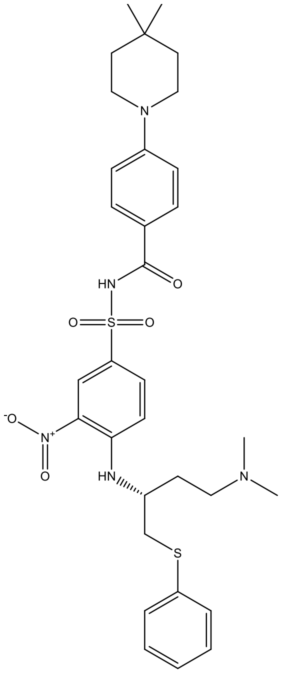
A-385358
406228-55-5
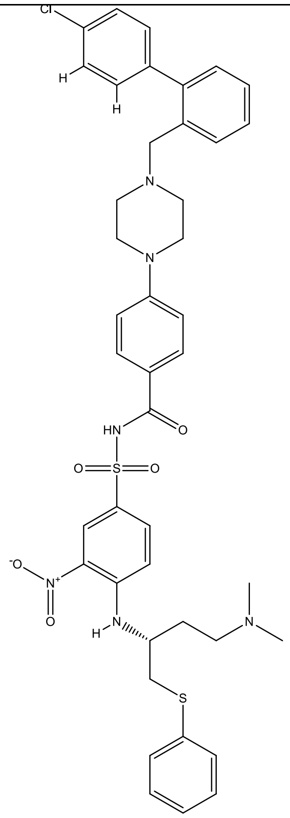
852808-04-9
ABT-737
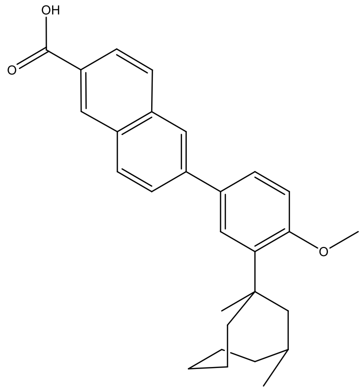
Adapalene
106685-40-9</td></tr>
</table>

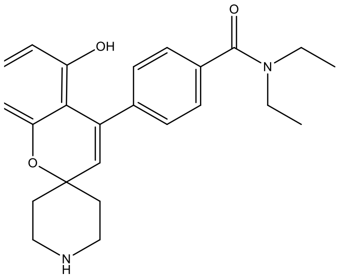

ADL5859
850305-06-5

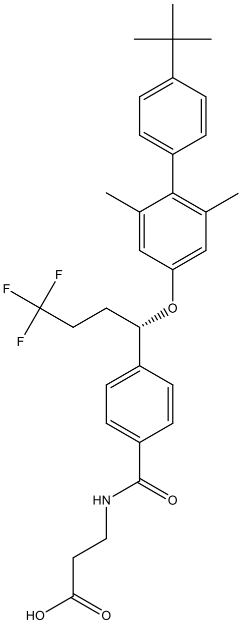

Adomeglivant
1488363-78-5

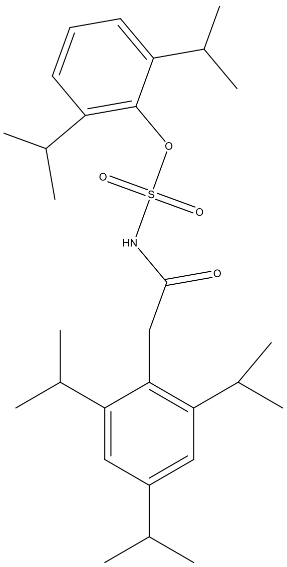

Avasimibe
166518-60-1

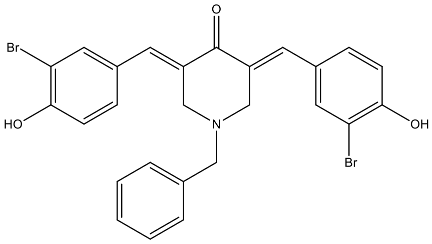

CARM1-IN-1
1020399-49-8

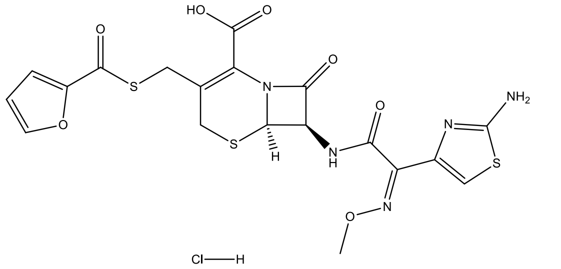

Ceftiofur HCl
103980-44-5

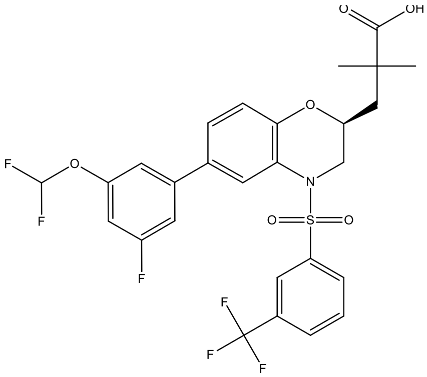

Cintirorgon
2055536-64-4

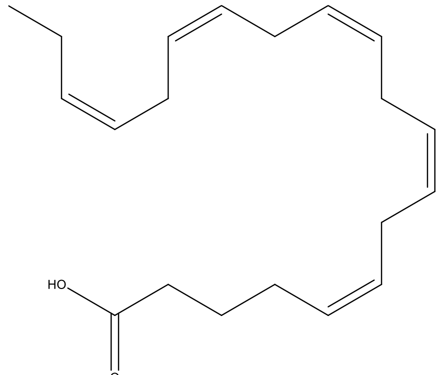

Eicosapentaenoic acid
10417-94-4

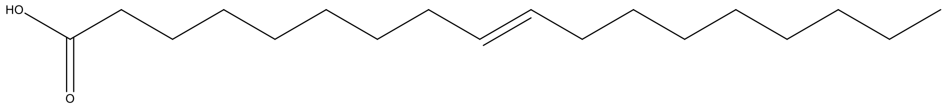

Elaidic acid
112-79-8

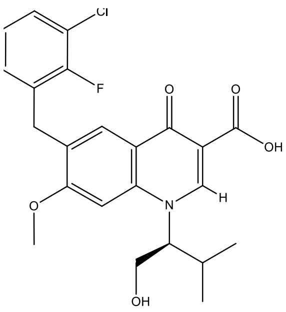

Elvitegravir
697761-98-1

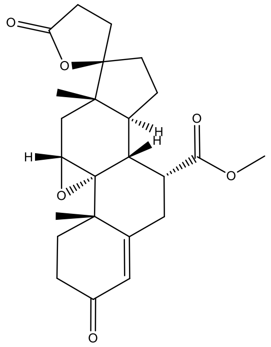

Eplerenone
107724-20-9

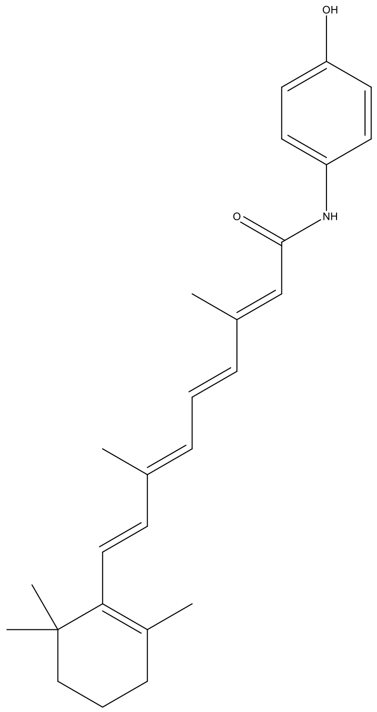

Fenretinide
65646-68-6

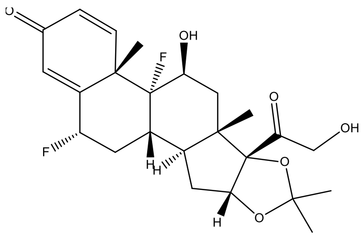

Fluocinolone Acetonide
67-73-2

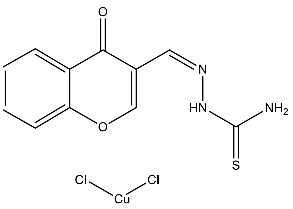

FPA-124
902779-59-3

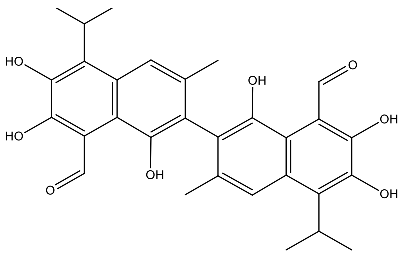

Gossypol
303-45-7

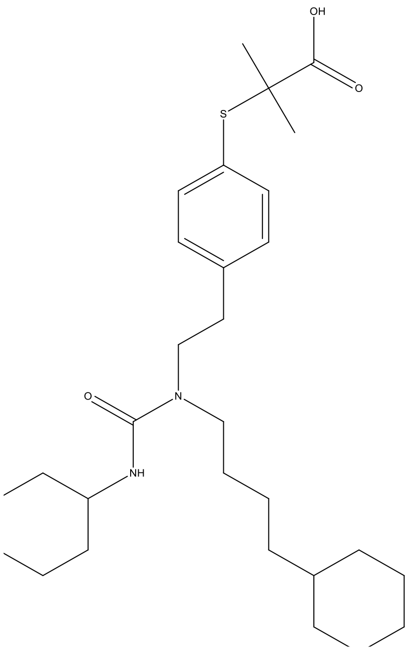

GW-7647
265129-71-3

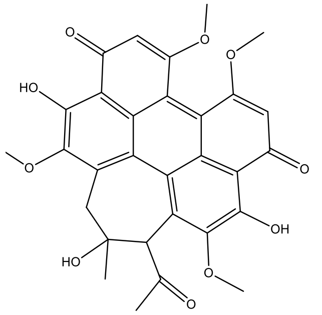

Hypocrellin A
77029-83-5

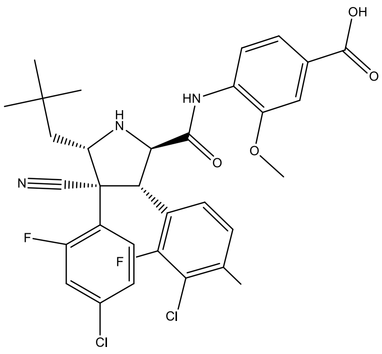

Idasanutlin

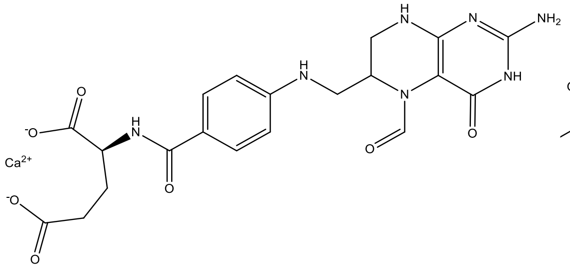

Leucovorin Calcium
1492-18-8

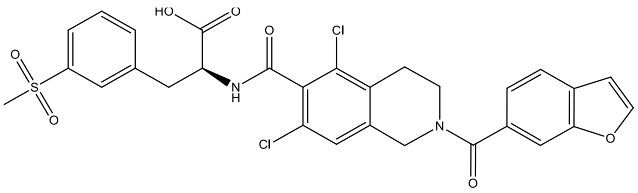

Lifitegrast
1025967-78-5

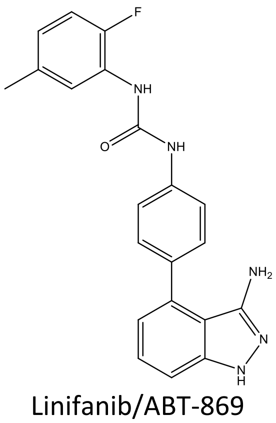

Linifanib/ABT-869
796967-16-3

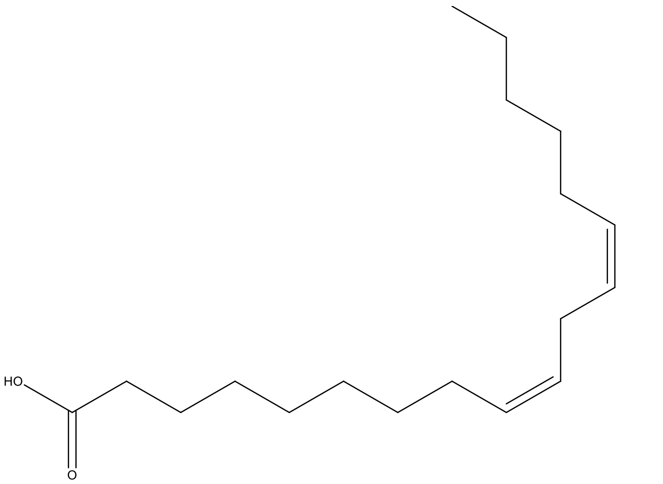

Linoleic acid
60-33-3

Mitoxantrone
See Mehyar et. al. (2021a)

Doxorubicin
See Mehyar et. al. (2021a)

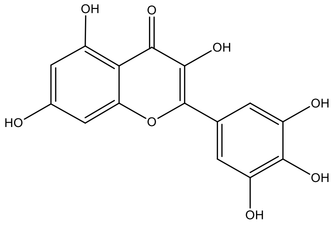

Myricetin
529-44-2

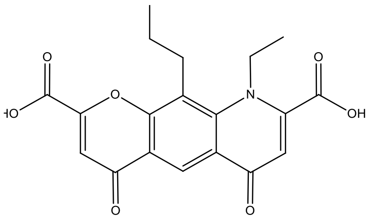

Nedocromil
69049-73-6

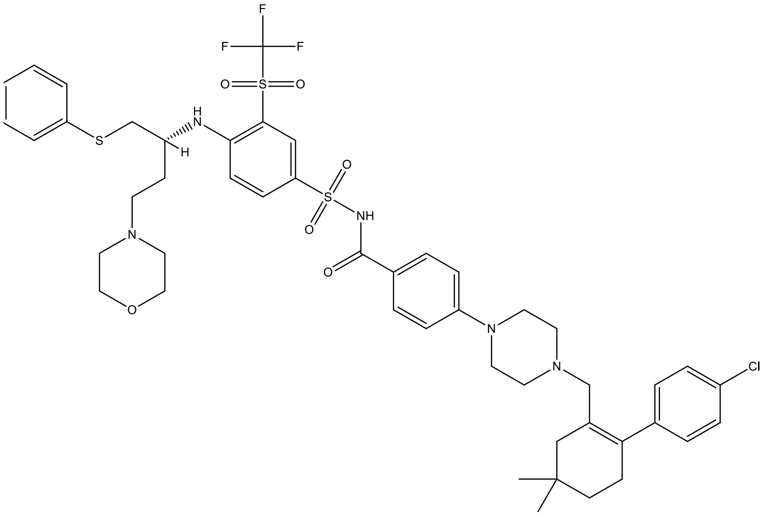

Navitoclax
923564-51-6

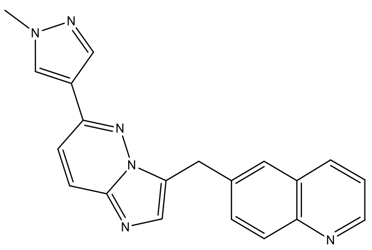

NVP-BVU-972
1185763-69-2

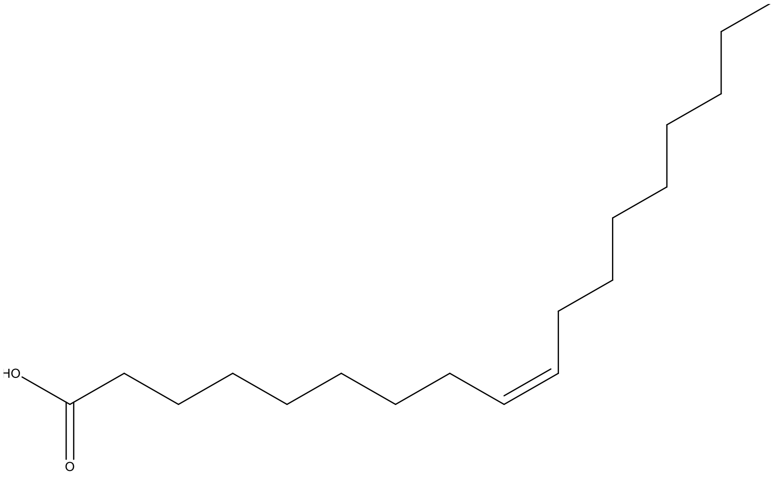

Oleic acid
112-80-1

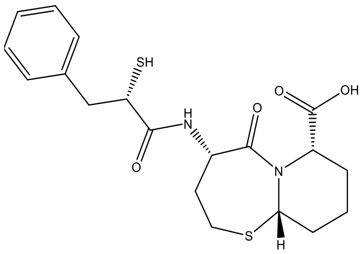

Omapatrilat
167305-00-2

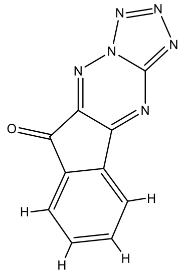

PDK1/Akt/Flt dual pathway inhibitor
331253-86-2

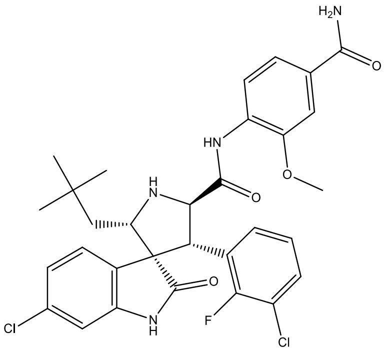

RO8994
1309684-94-3

SSYA10-001
See Adedeji et al. (2012a)

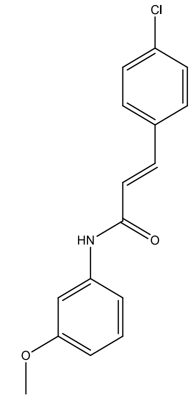

SB-366791
472981-92-3

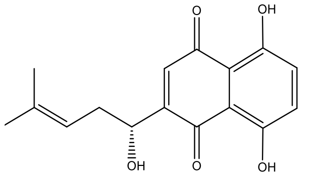

Shikonin
517-89-5

Zafirlukast
See Mehyar et al. (2021b)

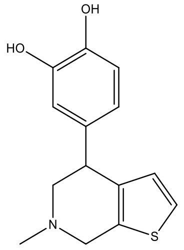

SKF 89145
79599-97-6

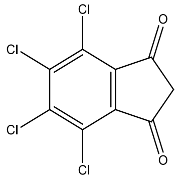

TCID
30675-13-9

	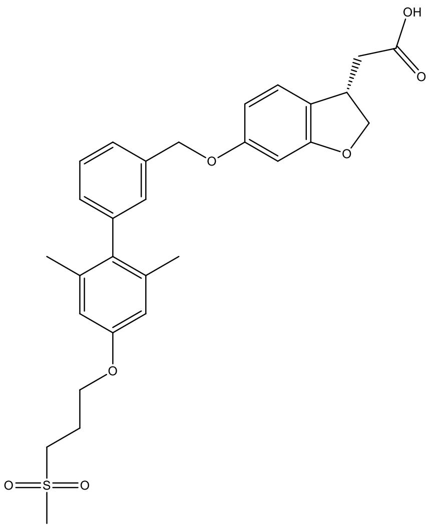<br>TAK875<br>1000413-72-8<br>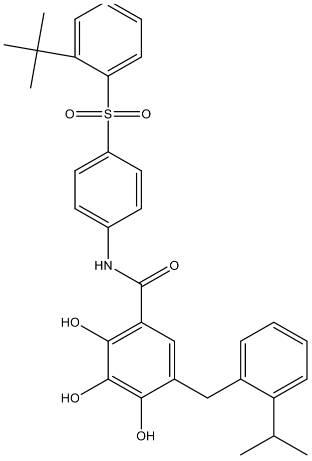<br>TW-37<br>877877-35-5<br>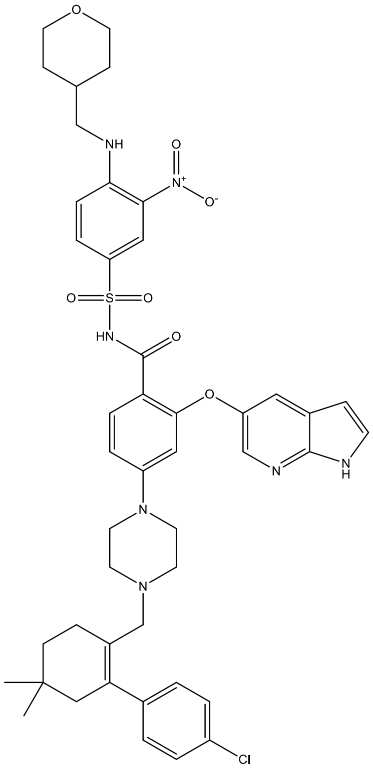<br>Venetoclax/ABT-199<br>1257044-40-8

Studies and molecules are arranged in alphabetical order
